# Supplementary material for: United States National Postdoc Survey results and the interaction of gender, career choice and mentor impact
Source: eLife. 2018 Dec 18;7:e40189. doi: 10.7554/eLife.40189 (PMC6298783; doi:10.7554/eLife.40189)
Supplement: Source data 2. [file elife-40189-data2.pdf]

2016 National Postdoc Survey

Section 1. Introduction

Thank you for participating in the 2016 National Postdoctoral Survey. This survey was conceived and developed by postdocs to benefit all members of the postdoctoral community. Specifically, 'postdoctoral' encompasses classical postdoc fellows and scholars as well as others beyond their doctoral work, including research associate/assistant professors and research professionals (and any others working in scientific training or temporary positions, post-PhD).

The information collected in this survey will facilitate identification of important issues within the postdoctoral community, informing and equipping those who advocate policy. Unless otherwise indicated, please answer with respect to your most recent (current) postdoctoral position. All of your answers and comments are completely anonymous, so please be as honest, candid, and critical as you see fit.

We estimate that completion of this survey will take 20 minutes on average. Upon completion of the survey, you will be redirected to a site where you may choose to enter your contact information into a drawing for a chance to win a $200 travel award to a scientific meeting of your choice.

Again, we thank you for your time, as well as your helpful comments and suggestions.

This is an anonymous survey. Answers will not be linked to any personal information.

IRB Protocol # IRB15-1724 (University of Chicago)

Institution or University

- ◯ Other - not listed
- ◯ A. T. Still University Of Health Sciences
- ◯ Aaron Diamond Aids Research Center
- ◯ Abilene Christian University
- ◯ Abraham Baldwin Agricultural College
- ◯ Adams State College
- ◯ Adelphi University
- ◯ Adrian College
- ◯ Aerospace Federally Funded Research And Development Center
- ◯ Agnes Scott College
- ◯ Aib College Of Business
- ◯ Aiken Technical College
- ◯ Aims Community College
- ◯ Air Force Institute Of Technology
- ◯ Alabama A&M University
- ◯ Alabama Southern Community College
- ◯ Alabama State University
- ◯ Alamo Community College
- ◯ Alaska Pacific University
- ◯ Albany College Of Pharmacy And Health Sciences
- ◯ Albany Medical College
- ◯ Albany Molecular Research
- ◯ Albany State University
- ◯ Albert Einstein Healthcare Network
- ◯ Albion College
- ◯ Albright College
- ◯ Alcorn State University
- ◯ Alderson-Broaddus College
- ◯ Alfred I. Du Pont Hospital For Children
- ◯ Alfred University
- ◯ Allan Hancock College
- ◯ Allegany College Of Maryland
- ◯ Allegheny College
- ◯ Allegheny University Of The Health Sciences
- ◯ Allegheny-Singer Research Institute
- ◯ Allen College- Waterloo
- ◯ Allen Institute
- ◯ Allen University
- ◯ Alliant International University
- ◯ Alma College
- ◯ Alvernia University
- ◯ Alverno College
- ◯ Amarillo College
- ◯ American Academy of Child and Adolescent Psychiatry
- ◯ American Association For Cancer Research
- ◯ American College Of Medical Genetics
- ◯ American College Of Radiology
- ◯ American College - Fayetteville
- ◯ American International College
- ◯ American Samoa Community College
- ◯ American Type Culture Collection
- ◯ American University Of Puerto Rico - Bayamon
- ◯ American University
- ◯ Ames Laboratory
- ◯ Amherst College
- ◯ Amridge University
- ◯ Ana G. Mendez University
- ◯ Andrew College
- ◯ Andrew Jackson University
- ◯ Andrews University
- ◯ Angelo State University
- ◯ Anna Maria College
- ◯ Anne Arundel Community College
- ◯ Anoka-Ramsey Community College
- ◯ Antaya Science And Technology
- ◯ Antelope Valley College
- ◯ Antioch University
- ◯ Appalachian State University
- ◯ Arapahoe Community College

◯ Arbor Research Collaborative For Health
◯ Arcadia University
◯ Argonne National Laboratory
◯ Argosy University
◯ Arizona State University
◯ Arizona Western College
◯ Arkansas Baptist College
◯ Arkansas Children's Hospital Research Institute
◯ Arkansas State University
◯ Arkansas Tech University
◯ Armstrong Atlantic State University
◯ Arrowhead Community Colleges
◯ Arroyo Center
◯ Art Center College Of Design
◯ Art Institute Of Seattle
◯ Asbury Theological Seminary
◯ Asheville-Buncombe Technical Community College
◯ Ashland University
◯ Assumption College
◯ Atenas College
◯ Atlanta University Center
◯ Atlantic Cape Community College
◯ Auburn University - Auburn
◯ Auburn University - Montgomery
◯ Augsburg College
◯ Augusta State University
◯ Augustana College - Rock Island
◯ Augustana College - Sioux Falls
◯ Aurora University
◯ Austin College
◯ Austin Community College - Northridge Campus
◯ Austin Peay State University
◯ Avila University
◯ Azusa Pacific University
◯ Babson College
◯ Baker College - Flint
◯ Baker University
◯ Baldwin-Wallace College
◯ Ball State University
◯ Baltimore City Community College
◯ Bank Street College Of Education
◯ Banner Alzheimer's Institute
◯ Banner Health
◯ Baptist Memorial College Of Health Sciences
◯ Bard College
◯ Bard College At Simon's Rock
◯ Barnard College
◯ Barnes-Jewish College Goldfarb School Of Nursing
◯ Barry University
◯ Barton County Community College
◯ Baruch S. Blumberg Institute
◯ Bastyr University
◯ Bates College
◯ Baton Rouge Community College
◯ Battelle Centers for Public Health Research and Evaluation
◯ Battelle Pacific Northwest Laboratories
◯ Bay Area Tumor Institute
◯ Bay De Noc Community College
◯ Bay Mills Community College
◯ Baylor College Of Medicine
◯ Baylor Research Institute
◯ Baylor University
◯ Baystate Medical Center
◯ Beaufort County Community College
◯ Bellarmine University
◯ Bellevue College
◯ Bellin College
◯ Belmont University
◯ Beloit College
◯ Bemidji State University

◯ Benaroya Research Institute At Virginia Mason
◯ Benedict College
◯ Benedictine College
◯ Benedictine University
◯ Benjamin Franklin Institute Of Technology
◯ Bennett College For Women
◯ Bennington College
◯ Bentley University
◯ Berea College
◯ Bergen Community College
◯ Berkeley College - New York City
◯ Berry College
◯ Bessemer State Technical College
◯ Beth Israel Deaconess Medical Center
◯ Beth Israel Medical Center (New York)
◯ Bethany College - Bethany
◯ Bethel College - Mishawaka - IN
◯ Bethel College - North Newton
◯ Bethel University
◯ Bethune-Cookman University
◯ Bevill State Community College Walker Campus
◯ Biola University
◯ Biomedical Research Institute
◯ Birmingham Southern College
◯ Bishop State Community College
◯ Bismarck State College
◯ Black Hawk College
◯ Black Hills State University
◯ Blackfeet Community College
◯ Blackhawk Technical College
◯ Blood Systems Research Institute
◯ Bloodcenter Of Wisconsin
◯ Bloomfield College
◯ Bloomsburg University Of Pennsylvania
◯ Bluefield State College
◯ Boise State University
◯ Boston Architectural Center
◯ Boston College
◯ Boston Medical Center
◯ Boston University
◯ Boston University Medical Campus
◯ Bowdoin College
◯ Bowie State University
◯ Bowling Green State University
◯ Bradley University
◯ Brandeis University
◯ Brazosport College
◯ Brenau University
◯ Brentwood Biomedical Research Institute
◯ Brescia University
◯ Brevard Community College
◯ Bridgewater College
◯ Bridgewater State College
◯ Brigham And Women's Hospital
◯ Brigham Young University
◯ Brigham Young University - Provo
◯ Bristol Community College
◯ Brite Divinity School
◯ Broad Institute
◯ Brookdale Community College
◯ Brookhaven National Laboratory
◯ Brookhaven Science Association - Brookhaven Lab
◯ Brooklyn Law School
◯ Broward College
◯ Brown Mackie College
◯ Brown University
◯ Brunswick Community College
◯ Bryant University
◯ Bryn Athyn College
◯ Bryn Mawr College
◯ Buck Institute For Research On Aging

◯ Bucknell University
◯ Bucks County Community College
◯ Buena Vista University
◯ Bunker Hill Community College
◯ Burlington County College
◯ Butler County Community College (Butler PA)
◯ Butler Hospital (Providence RI)
◯ Butler University
◯ Butte College
◯ Cabrillo College
◯ Cabrini College
◯ Calhoun Community College
◯ California Baptist University
◯ California College Of Arts And Crafts
◯ California Community College
◯ California Institute Of Integral Studies
◯ California Institute Of Technology
◯ California Institute Of The Arts
◯ California Lutheran University
◯ California Maritime Academy
◯ California Pacific Medical Center Research Institute
◯ California Polytechnic State University - San Luis Obispo
◯ California School Of Professional Psychology - Berkeley-Alameda
◯ California School Of Professional Psychology - Fresno
◯ California School Of Professional Psychology - Los Angeles
◯ California State Polytechnic University - Pomona
◯ California State University Unallocated
◯ California State University - Bakersfield
◯ California State University - Channel Islands
◯ California State University - Chico
◯ California State University - Dominguez Hills
◯ California State University - East Bay
◯ California State University - Fresno
◯ California State University - Fullerton
◯ California State University - Long Beach
◯ California State University - Los Angeles
◯ California State University - Monterey Bay
◯ California State University - Northridge
◯ California State University - Sacramento
◯ California State University - San Bernardino
◯ California State University - San Marcos
◯ California State University - Stanislaus
◯ California University Of Pennsylvania
◯ Calvin College
◯ Calvin Theological Seminary
◯ Cambridge Health Alliance
◯ Camden County College
◯ Cameron University
◯ Campbell University
◯ Cancer Prevention Institute Of California
◯ Canisius College
◯ Cankdeska Cikana Community College
◯ Cape Cod Community College
◯ Cape Fear Community College
◯ Capella University
◯ Capital University
◯ Capitol College
◯ Cardinal Stritch University
◯ Caribbean University
◯ Carl Albert State College
◯ Carl Sandburg College
◯ Carleton College
◯ Carlos Albizu University
◯ Carlow University
◯ Carnegie Mellon University
◯ Carolinas Medical Center

◯ Carroll College
◯ Carroll University
◯ Carson-Newman College
◯ Carthage College
◯ Case Western Reserve University
◯ Casper College
◯ Castleton State College
◯ Catawba College
◯ Catawba Valley Community College
◯ Catholic University Of America
◯ Cayuga Community College
◯ Cedar Crest College
◯ Cedars-Sinai Medical Center
◯ Centenary College
◯ Centenary College Of Louisiana
◯ Center For Advanced Aviation System Development
◯ Center For Communications And Computing
◯ Center For Construction Research And Training
◯ Center For Enterprise Modernization
◯ Center For Expermental Software Engr Md
◯ Center For Innovative Public Health Res
◯ Center For Naval Analyses
◯ Center For Nuclear Waste Regulatory Analyses
◯ Center For Psychological Consultation
◯ Center For Social Innovation
◯ Central Alabama Community College
◯ Central Arizona College
◯ Central College
◯ Central Community College
◯ Central Connecticut State University
◯ Central Florida Community College
◯ Central Georgia Technical College
◯ Central Lakes College - Brainerd
◯ Central Louisiana Technical College
◯ Central Maine Community College
◯ Central Michigan University
◯ Central New Mexico Community College
◯ Central Oregon Community College
◯ Central Piedmont Community College
◯ Central State University
◯ Central Washington University
◯ Central Wyoming College
◯ Centralia College
◯ Centre College
◯ Centro De Estudios Multidisciplinarios
◯ Century College
◯ Cerritos College
◯ Chadron State College
◯ Chaminade University Of Honolulu
◯ Chancellor University
◯ Chapman University
◯ Charles R. Drew University Of Medical & Sci
◯ Charles R. Drew University Of Medicine And Science
◯ Charles River Laboratories
◯ Charles Stark Draper Laboratory
◯ Charleston Southern University
◯ Chatham University - Pittsburgh
◯ Chattanooga State Community College
◯ Chemeketa Community College
◯ Chesapeake College
◯ Chestnut Health Systems
◯ Chestnut Hill College
◯ Cheyenne River Community College
◯ Cheyney University Of Pennsylvania
◯ CHI Institute For Research & Innovation
◯ Chicago School Of Professional Psychology
◯ Chicago State University
◯ Chicago Theological Seminary
◯ Chief Dull Knife College
◯ Children's Hospital Of Philadelphia
◯ Children's Hospital & Research Center At Oakland

◯ Children's Hospital Corporation
◯ Children's Hospital Of Los Angeles
◯ Children's Mercy Hospital (Kansas City MO)
◯ Children's Research Institute
◯ Chippewa Valley Technical College
◯ Chowan University
◯ Christian Brothers University
◯ Christopher Newport University
◯ Cincinnati Childrens Hospital Medical Center
◯ Cincinnati State Technical And Community College
◯ Citadel Military College Of South Carolina
◯ City College Of San Francisco
◯ City Colleges Of Chicago
◯ City Of Hope
◯ City Of Hope / Beckman Research Institute
◯ City University Of New York Unallocated
◯ City University Of New York - School of Law
◯ City University Of New York - Graduate Center
◯ City University Of New York - Baruch College
◯ City University Of New York - Borough Of Manhattan Community College
◯ City University Of New York - Bronx Community College
◯ City University Of New York - Brooklyn College
◯ City University Of New York - College Of Staten Island
◯ City University Of New York - Graduate Center
◯ City University Of New York - Hostos Community College
◯ City University Of New York - Hunter College
◯ City University Of New York - John Jay College Criminal Justice
◯ City University Of New York - Kingsborough Community College
◯ City University Of New York - Laguardia Community College
◯ City University Of New York - Lehman College
◯ City University Of New York - Medgar Evers College
◯ City University Of New York - New York City College Of Technology
◯ City University Of New York - Queens College
◯ City University Of New York - Queensborough Community College
◯ City University Of New York
◯ City University Of New York - The City University
◯ City University Of New York - York College
◯ Clackamas Community College
◯ Claflin University
◯ Claremont Graduate University
◯ Claremont Mckenna College
◯ Claremont School Of Theology
◯ Clarion University Of Pennsylvania
◯ Clark Atlanta University
◯ Clark College
◯ Clark State Community College
◯ Clark University
◯ Clarke University
◯ Clarkson College
◯ Clarkson University
◯ Clatsop Community College
◯ Clayton State University
◯ Cleary University
◯ Clemson University
◯ Cleveland Clinic Lerner College of Medicine of CWRU
◯ Cleveland Community College
◯ Cleveland Institute Of Music
◯ Cleveland State Community College
◯ Cleveland State University
◯ Clinton Junior College
◯ Cloud County Community College
◯ Cms Alliance To Modernize Healthcare

◯ Coastal Bend College
◯ Coastal Carolina Community College
◯ Coastal Carolina University
◯ Coastline Community College
◯ Cochise College
◯ Coe College
◯ Coker College
◯ Colby College
◯ Colby Community College
◯ Colby-Sawyer College
◯ Cold Spring Harbor Laboratory
◯ Colgate University
◯ College Of Charleston
◯ College Of Dupage
◯ College Of Idaho
◯ College Of Lake County
◯ College Of Menominee Nation
◯ College Of Micronesia - FSM
◯ College Of Mount Saint Vincent
◯ College Of Mount St. Joseph
◯ College Of Nanoscale Science And Engineering Of The University At Albany Suny
◯ College Of New Jersey
◯ College Of New Rochelle
◯ College Of Saint Benedict
◯ College Of Saint Rose
◯ College Of Southern Idaho
◯ College Of Southern Maryland
◯ College Of Southern Nevada
◯ College Of St. Elizabeth
◯ College Of St. Mary
◯ College Of St. Scholastica - Duluth
◯ College Of The Atlantic
◯ College Of The Canyons
◯ College Of The Holy Cross
◯ College Of The Mainland
◯ College Of The Sequoias
◯ College Of William And Mary
◯ College Of Wooster
◯ Collin County Community College
◯ Colorado Cancer Research Program
◯ Colorado College
◯ Colorado Mountain College
◯ Colorado School Of Mines
◯ Colorado State University
◯ Colorado State University - Pueblo
◯ Colorado State University
◯ Colorado Theological Seminary
◯ Columbia Basin College
◯ Columbia College Chicago
◯ Columbia College - Columbia MO
◯ Columbia College - Columbia SC
◯ Columbia University New York Morningside
◯ Columbia University Health Sciences
◯ Columbia University In The City Of New York
◯ Columbia University Teachers College
◯ Columbus Community Clinical Oncology Program
◯ Columbus State Community College
◯ Columbus State University
◯ Comanche Nation College
◯ Commonwealth Medical College
◯ Community College Of Allegheny County
◯ Community College Of Aurora
◯ Community College Of Baltimore County Catonsville
◯ Community College Of Philadelphia
◯ Community College Of Rhode Island
◯ Community Colleges Of Spokane
◯ Concord University
◯ Concordia College
◯ Concordia Seminary
◯ Concordia Theological Seminary

◯ Concordia University Chicago
◯ Concordia University Wisconsin
◯ Connecticut Children's Medical Center
◯ Connecticut College
◯ Connecticut State Dept Of Public Health
◯ Connecticut State University
◯ Connors State College
◯ Contra Costa College
◯ Contra Costa Community College
◯ Contra Costa Community College - San Ramon - Diablo Valley College
◯ Converse College
◯ Cooper Institute
◯ Cooper Union For The Advancement Of Science And Art
◯ Coppin State University
◯ Coriell Institute For Medical Research
◯ Cornell College
◯ Cornell University
◯ Corning Community College
◯ County College Of Morris
◯ Covenant College
◯ Cox College Of Nursing And Health Sciences
◯ Craven Community College
◯ Creighton University
◯ Crowder College
◯ Crown College
◯ Cuyahoga Community College
◯ Daemen College
◯ Dakota County Technical College
◯ Dakota State University
◯ Dakota Wesleyan University
◯ Dallas Community College - Eastfield College
◯ Dallas Community College - Richland College
◯ Dallas Theological Seminary
◯ Dana-Farber Cancer Institute
◯ Dartmouth College
◯ Darton College
◯ Data Numerica Institute
◯ Davidson College
◯ Davidson County Community College
◯ Davis & Elkins College
◯ Dayton Clinical Oncology Program
◯ Daytona State College
◯ De Anza College
◯ Decatur Memorial Hospital
◯ Defense Acquisition University
◯ Del Mar College
◯ Delaware State University
◯ Delaware Technical Community College
◯ Delaware Valley College
◯ Delgado Community College
◯ Delta State University
◯ Denison University
◯ Denmark Technical College
◯ Denver Health And Hospital Authority
◯ Depaul University
◯ Depauw University
◯ Des Moines Area Community College
◯ Des Moines University
◯ Desales University
◯ Desert Research Institute
◯ Dickinson College
◯ Dickinson State University
◯ Dillard University
◯ Dine College
◯ Dixie State College Of Utah
◯ Doane College
◯ Doheny Eye Institute
◯ Dominican College Of Blauvelt
◯ Dominican University
◯ Dominican University Of California

◯ Donnelly College
◯ Dordt College
◯ Dowling College
◯ D-Q University
◯ Drake University
◯ Drew University
◯ Drexel University
◯ Drury University
◯ Duke University
◯ Duquesne University
◯ Durham Technical Community College
◯ Dyersburg State Community College
◯ D'Youville College
◯ Earlham College
◯ East Arkansas Community College
◯ East Carolina University
◯ East Central University
◯ East Los Angeles College
◯ East Mississippi Community College
◯ East Stroudsburg University Of Pennsylvania
◯ East Tennessee State University
◯ Eastern Arizona College
◯ Eastern Connecticut State University
◯ Eastern Idaho Technical College
◯ Eastern Illinois University
◯ Eastern Iowa Community College
◯ Eastern Kentucky University
◯ Eastern Mennonite University
◯ Eastern Michigan University
◯ Eastern New Mexico University
◯ Eastern Oklahoma State College
◯ Eastern Oregon University
◯ Eastern Virginia Medical School
◯ Eastern Washington University
◯ East-West University
◯ Eckerd College
◯ ECOG-ACRIN Medical Research Foundation
◯ Ecpi College Of Technology
◯ Edinboro University Of Pennsylvania
◯ Edison State College
◯ Edison State Community College
◯ Edmonds Community College
◯ Edward Via College Of Osteopathic Medicine
◯ Edward Waters College
◯ El Camino College
◯ El Camino College Compton Center
◯ El Paso Community College
◯ Elgin Community College
◯ Elizabeth City State University
◯ Elizabethtown College
◯ Elmhurst College
◯ Elms College
◯ Elon University
◯ Emanuel Hospital And Health Center
◯ Embry-Riddle Aeronautical University
◯ Emergent Product Development Gaithersbur
◯ Emerson College
◯ Emma Pendleton Bradley Hospital
◯ Emmanuel College
◯ Emory & Henry College
◯ Emory University
◯ Emporia State University
◯ Endicott College
◯ Erie Community College North Campus
◯ Erikson Institute
◯ Erskine College
◯ Essentia Institute Of Rural Health
◯ Essex County College
◯ Everett Community College
◯ Evergreen State College - Olympia
◯ Evergreen Valley College

◯ Experimental Pathology Laboratories
◯ Fairfield University
◯ Fairleigh Dickinson University
◯ Fairmont State University
◯ Family Health International
◯ Fayetteville State University
◯ Fayetteville Technical Community College
◯ Feinstein Institute For Medical Research
◯ Felician College
◯ Fermi National Accelerator Laboratory
◯ Ferris State University
◯ Ferrum College
◯ Fielding Graduate University
◯ Finger Lakes Community College
◯ Finlandia University
◯ Fisk University
◯ Fitchburg State University
◯ Flathead Valley Community College
◯ Florence-Darlington Technical College
◯ Florida Agricultural And Mechanical University
◯ Florida Atlantic University
◯ Florida Gulf Coast University
◯ Florida Hospital College Of Health Sciences
◯ Florida Institute Of Technology
◯ Florida International University
◯ Florida Keys Community College
◯ Florida Memorial University
◯ Florida Metropolitan University Tampa Campus
◯ Florida Southern College
◯ Florida State College At Jacksonville
◯ Florida State University
◯ Fond Du Lac Tribal And Community College
◯ Foothill-De Anza Community College
◯ Fordham University
◯ Forsyth Institute
◯ Forsyth Technical Community College
◯ Fort Belknap College
◯ Fort Berthold Community College
◯ Fort Hays State University
◯ Fort Lewis College
◯ Fort Peck Community College
◯ Fort Valley State University
◯ Foundation For Aids Research
◯ Fox Chase Chemical Diversity Center
◯ Fox Valley Technical College
◯ Framingham State College
◯ Francis Marion University
◯ Franciscan University Of Steubenville
◯ Frank Phillips College
◯ Franklin & Marshall College
◯ Franklin Pierce Law Center
◯ Franklin Pierce University - Rindge
◯ Franklin W. Olin College Of Engineering
◯ Fred Hutchinson Cancer Research Center
◯ Frederick National Laboratory For Cancer Research
◯ Fresno City College
◯ Friends Research Institute
◯ Frontier School Of Midwifery And Family Nursing
◯ Frostburg State University
◯ Fuller Theological Seminary - Pasadena
◯ Fulton-Montgomery Community College
◯ Furman University
◯ Future Generations Graduate School
◯ Gadsden State Community College
◯ Gallaudet University
◯ Gannon University
◯ Garrett College
◯ Garrett-Evangelical Theological Seminary
◯ Gaston College
◯ Gateway Community And Technical College
◯ Gateway Technical College

◯ Geisinger Clinic
◯ General Electric Global Research Center
◯ Genesee Community College
◯ Geophysical Institute - University of Alaska Fairbanks
◯ George Fox University
◯ George Mason University
◯ George Washington University
◯ Georgetown College
◯ Georgetown University
◯ Georgia College And State University
◯ Georgia Health Sciences University
◯ Georgia Institute Of Technology
◯ Georgia Perimeter College
◯ Georgia Regents University
◯ Georgia Southern University
◯ Georgia Southwestern State University
◯ Georgia State University
◯ Georgian Court University
◯ Gettysburg College
◯ Glen Oaks Community College
◯ Glendale Community College - Glendale
◯ Glenville State College
◯ Globe University - Minnesota School Of Business
◯ Goddard College
◯ Gogebic Community College
◯ Golden Gate University
◯ Gonzaga University
◯ Goodwin College
◯ Gordon College
◯ Goshen College
◯ Goucher College
◯ Governors State University
◯ Graceland University
◯ Graduate School Usa
◯ Graduate Theological Union
◯ Grambling State University
◯ Grand Rapids Community College
◯ Grand Valley State University
◯ Grand View University
◯ Grayson County College
◯ Green Mountain College
◯ Green River Community College
◯ Greenfield Community College
◯ Greenville College
◯ Greenville Health System
◯ Greenville Technical College
◯ Grinnell College
◯ Grossmont College
◯ Grossmont-Cuyamaca Community College
◯ Group Health Cooperative
◯ Guam Community College
◯ Guilford College
◯ Gulf Coast Community College
◯ Gustavus Adolphus College
◯ Gwynedd-Mercy College
◯ H. Lee Moffitt Cancer Center & Research Institute
◯ Hackensack University Medical Center
◯ Hagerstown Community College
◯ Hamilton College
◯ Hamline University
◯ Hampden-Sydney College
◯ Hampshire College
◯ Hampton University
◯ Harcum College
◯ Harding University - Searcy
◯ Harford Community College
◯ Harper College
◯ Harrisburg Area Community College
◯ Harrisburg University Of Science And Technology
◯ Harris-Stowe State University

◯ Hartford Hospital
◯ Hartnell College
◯ Hartwick College
◯ Harvard Medical School
◯ Harvard Pilgrim Health Care
◯ Harvard School Of Public Health
◯ Harvard University
◯ Harvey Mudd College
◯ Haskell Indian Nations University
◯ Hauptman-Woodward Medical Research Institute
◯ Haverford College
◯ Hawaii Pacific University
◯ Haywood Community College
◯ Healthpartners Institute
◯ Heartland Community College
◯ Hebrew Union College-Jewish Institute Of Religion
◯ Heidelberg University
◯ Hektoen Institute For Medical Research
◯ Helene Fuld College Of Nursing
◯ Henderson State University
◯ Hendrix College
◯ Henry Ford Community College
◯ Henry Ford Health System
◯ Henry M. Jackson Foundation for the Advancement of Military Medicine
◯ Heritage University
◯ High Point University
◯ Highland Community College - Freeport - Il
◯ Highland Community College - Highland
◯ Highline Community College
◯ Hillsborough Community College
◯ Hinds Community College
◯ Hiram College
◯ Hobart And William Smith Colleges
◯ Hocking College - Nelsonville
◯ Hofstra University
◯ Hollins University
◯ Holy Family University
◯ Holyoke Community College
◯ Homeland Security Studies And Analysis Institute
◯ Homeland Security Systems Engineering And Development Institute
◯ Hood College
◯ Hope College
◯ Horry-Georgetown Technical College
◯ Hospital For Special Surgery
◯ Houston Baptist University
◯ Houston Community College
◯ Howard Community College
◯ Howard University
◯ Hudson Valley Community College
◯ Hudson-Alpha Institute For Biotechnology
◯ Hugo W. Moser Research Institute Kennedy Krieger
◯ Humboldt State University
◯ Huntington Medical Research Institutes
◯ Husson University
◯ Huston-Tillotson University
◯ Hutchinson Community College And Area Vocational School
◯ Icahn School Of Medicine At Mount Sinai
◯ Idaho National Laboratory
◯ Idaho State University
◯ IIT Research Institute
◯ Ilisagvik College
◯ Illinois College Of Optometry
◯ Illinois Institute Of Technology
◯ Illinois State University
◯ Illinois Valley Community College
◯ Illinois Wesleyan University
◯ Immaculata University
◯ Imperial Valley College

◯ Indian Hills Community College
◯ Indian River State College
◯ Indiana Institute Of Technology
◯ Indiana State University
◯ Indiana University
◯ Indiana University Of Pennsylvania
◯ Indiana University - Bloomington
◯ Indiana University - Gary
◯ Indiana University - New Albany
◯ Indiana University - Richmond
◯ Indiana University - South Bend
◯ Indiana University - Purdue University - Fort Wayne
◯ Indiana University -Purdue University - Indianapolis- IUPUI
◯ Indiana Wesleyan University
◯ Infectious Disease Research Institute
◯ Inland Northwest Research Alliance
◯ Innovation Research And Training
◯ Institute For Clinical Social Work - Chicago
◯ Institute For Community Research
◯ Institute For Molecular Medicine
◯ Institute For Systems Biology
◯ Institute Of American Indian Arts
◯ Institute Of Transpersonal Psychology
◯ Inter American University Of Puerto Rico
◯ Inver Hills Community College
◯ Iona College
◯ Iowa Central Community College
◯ Iowa Lakes Community College
◯ Iowa State University
◯ Iowa Valley Community College
◯ Iowa Western Community College
◯ Irvine Valley College
◯ Itawamba Community College
◯ Ithaca College
◯ ITT Technical Institute - Austin TX
◯ ITT Technical Institute - Evansville IN
◯ Ivy Tech Community College
◯ J. Craig Venter Institute
◯ J. David Gladstone Institutes
◯ J.F. Drake State Technical College
◯ Jackson State Community College
◯ Jackson State University
◯ Jacksonville State University
◯ Jacksonville University
◯ Jaeb Center For Health Research
◯ James Madison University
◯ Jamestown Community College
◯ Jarvis Christian College
◯ Jefferson College Of Health Sciences
◯ Jefferson Davis Community College
◯ Jefferson State Community College
◯ Jet Propulsion Laboratory
◯ Jewish Theological Seminary Of America
◯ John A. Logan College
◯ John B. Pierce Laboratory
◯ John Bastyr College Of Naturopathic Medicine
◯ John Brown University
◯ John Carroll University
◯ John F. Kennedy University- Pleasant Hill
◯ John Wayne Cancer Institute
◯ Johns Hopkins University
◯ Johnson C. Smith University
◯ Johnson County Community College
◯ Johnson State College
◯ Joliet Junior College
◯ Jones County Junior College
◯ Joslin Diabetes Center
◯ Judiciary Engineering And Modernization Center
◯ Judson University- Elgin
◯ Juilliard School

◯ Juniata College
◯ Kalamazoo College
◯ Kankakee Community College
◯ Kansas City Kansas Community College
◯ Kansas City University Of Medicine And Biosciences
◯ Kansas State University
◯ Kaplan College - Las Vegas
◯ Kaskaskia College
◯ Kean University
◯ Keck Graduate Institute
◯ Keene State College
◯ Kennebec Valley Community College
◯ Kennesaw State University
◯ Kent State University
◯ Kentucky Community And Technical College
◯ Kentucky State University
◯ Kenyon College
◯ Kettering University
◯ Keuka College
◯ Keweenaw Bay Ojibwa Community College
◯ Keystone College
◯ King College
◯ King's College - Wilkes Barre
◯ Kirkwood Community College
◯ Kishwaukee College
◯ Knox College
◯ Kutztown University Of Pennsylvania
◯ La BioMedical Research Institute / Harbor UCLA Medical Center
◯ La Jolla Bioengineering Institute
◯ La Jolla Infectious Disease Institute
◯ La Jolla Institute For Allergy & Immunolgy
◯ La Roche College
◯ La Salle University
◯ La Sierra University
◯ Lac Courte Oreilles Ojibwa Community College
◯ Lafayette College
◯ Lake City Community College
◯ Lake Erie College Of Osteopathic Medicine
◯ Lake Forest College
◯ Lake Land College
◯ Lake Michigan College
◯ Lake Superior State University
◯ Lakeshore Technical College
◯ Lamar State College - Orange
◯ Lamar University
◯ Lancaster General College Of Nursing And Health Sciences
◯ Lander University
◯ Landmark College
◯ Lane College
◯ Lane Community College
◯ Langston University
◯ Lankenau Institute For Medical Research
◯ Lansing Community College
◯ Laramie County Community College
◯ Laredo Community College
◯ Lasell College
◯ Laureate Institute For Brain Research
◯ Lawrence Berkeley National Laboratory
◯ Lawrence Livermore National Laboratory
◯ Lawrence Technological University
◯ Lawrence University
◯ Lawson State Community College
◯ Le Moyne College
◯ Lebanon Valley College
◯ Lee College
◯ Lee University
◯ Leech Lake Tribal College
◯ Lehigh Carbon Community College
◯ Lehigh University

◯ Leidos Biomedical Research
◯ Lemoyne-Owen College
◯ Lenoir Community College
◯ Lesley University
◯ Letourneau University
◯ Lewis & Clark College
◯ Lewis And Clark Community College
◯ Lewis University
◯ Lewis-Clark State College
◯ Liberty University
◯ Lieber Institute
◯ Lincoln Laboratory
◯ Lincoln Memorial University
◯ Lincoln University Of The Commonwealth Of Pennsylvania
◯ Lincoln University - Jefferson City
◯ Linfield College
◯ Linn Benton Community College
◯ Lipscomb University
◯ Little Big Horn College
◯ Little Priest Tribal College
◯ Livingstone College
◯ Lock Haven University - Lock Haven
◯ Logan College Of Chiropractic
◯ Loma Linda University
◯ Loma Linda Veterans Association Research & Education
◯ Long Beach City College
◯ Long Island University - Brooklyn
◯ Long Island University - Brookville
◯ Longwood University
◯ Lorain County Community College
◯ Loras College
◯ Los Alamos National Lab - National Security Science
◯ Los Alamos National Laboratory
◯ Los Angeles City College
◯ Los Angeles College Of Chiropractic
◯ Los Angeles Community College
◯ Los Angeles County College Of Nursing And Allied Health
◯ Los Angeles Southwest College
◯ Los Angeles Valley College
◯ Los Rios Community College
◯ Louis V. Gerstner Jr. Graduate School Of Biomedical Sciences At Memorial
◯ Louisburg College
◯ Louisiana State University Health Science Center - Shreveport
◯ Louisiana State University
◯ Louisiana State University Medical Center Shreveport
◯ Louisiana State University - Baton Rouge
◯ Louisiana State University - New Orleans - Health Sciences Center
◯ Louisiana State University - Shreveport
◯ Louisiana Tech University
◯ Lourdes College
◯ Lovelace Biomedical & Environmental Res
◯ Loyola Marymount University
◯ Loyola University Chicago
◯ Loyola University Maryland
◯ Loyola University New Orleans
◯ Lsu Health Sciences Center
◯ Lsu Pennington Biomedical Research Center
◯ Ludwig Institute For Cancer Research Ltd
◯ Lurleen B. Wallace Community College
◯ Luther College
◯ Luther Seminary
◯ Lutheran School Of Theology At Chicago
◯ Lutheran Theological Seminary At Philadelphia
◯ Lycoming College

◯ Lynchburg College
◯ Lyndon State College
◯ Lynn University
◯ Lyon College
◯ Macalester College
◯ Macomb Community College
◯ Madison Area Technical College
◯ Madonna University
◯ Magee-Women's Research Institute And Foundation
◯ Maharishi University Of Management
◯ Maine Maritime Academy
◯ Maine Medical Center
◯ Malone University
◯ Manchester Community College
◯ Manhattan Area Technical College
◯ Manhattan College
◯ Manhattan School Of Music
◯ Mansfield University Of Pennsylvania
◯ Marian University - Fond Du Lac
◯ Marian University - Indianapolis
◯ Maricopa Community College
◯ Marietta College
◯ Marine Biological Laboratory
◯ Marine Corps University
◯ Marion Military Institute
◯ Marist College
◯ Marlboro College
◯ Marquette University
◯ Marshall University
◯ Martin University
◯ Mary Baldwin College
◯ Mary Holmes College
◯ Marygrove College
◯ Maryland Institute College Of Art
◯ Marymount University
◯ Maryville College
◯ Maryville University Of St. Louis
◯ Marywood University
◯ Massachusetts Bay Community College
◯ Massachusetts Board Of Higher Education
◯ Massachusetts College Of Liberal Arts
◯ Massachusetts College Of Pharmacy And Health Sciences
◯ Massachusetts Eye And Ear Infirmary
◯ Massachusetts General Hospital
◯ Massachusetts Institute Of Technology
◯ Massachusetts Maritime Academy
◯ Massachusetts State College
◯ Massasoit Community College
◯ Maui Community College
◯ Max Planck Florida Corporation
◯ Mayland Community College
◯ Mayo Clinic Arizona
◯ Mayo Clinic Jacksonville
◯ Mayo Clinic Rochester
◯ Mayo Graduate School
◯ Mayville State University
◯ Mccormick Theological Seminary
◯ Mcdaniel College
◯ Mckendree University
◯ Mclean Hospital
◯ Mcneese State University
◯ Medaille College
◯ Medical College Of Wisconsin
◯ Medical University Of South Carolina
◯ Medstar Health Research Institute
◯ Meharry Medical College
◯ Merced Community College
◯ Mercer University
◯ Mercy College Of Ohio
◯ Mercy College

◯ Mercyhurst College
◯ Meredith College
◯ Merrimack College
◯ Merritt College
◯ Mesa State College
◯ Messiah College
◯ Methodist Hospital Research Institute
◯ Methodist University
◯ Metropolitan College Of New York
◯ Metropolitan Community College - Fort Omaha Campus
◯ Metropolitan Community College - Kansas City
◯ Metropolitan Community College - Penn Valley
◯ Metropolitan State College Of Denver
◯ Metropolitan State University
◯ Mgh Institute Of Health Professions
◯ Miami Dade College
◯ Miami University
◯ Michigan Public Health Institute
◯ Michigan State University
◯ Michigan Technological University
◯ Mid Michigan Community College
◯ Mid-America Baptist Theological Seminary
◯ Middle Tennessee School Of Anesthesia
◯ Middle Tennessee State University
◯ Middlebury College
◯ Middlesex Community College
◯ Middlesex County College
◯ Midland College
◯ Midlands Technical College
◯ Mid-South Community College
◯ Midwest Research Institute
◯ Midwestern Baptist Theological Seminary
◯ Midwestern State University
◯ Midwestern University
◯ Miles College
◯ Millersville University Of Pennsylvania
◯ Millikin University
◯ Mills College
◯ Millsaps College
◯ Milwaukee Area Technical College
◯ Milwaukee Institute Of Art & Design
◯ Milwaukee School Of Engineering
◯ Mineral Area College
◯ Minneapolis Community And Technical College
◯ Minneapolis Medical Research Fdn
◯ Minnesota State Colleges And Universities
◯ Minnesota State Community And Technical College
◯ Minnesota State University - Mankato
◯ Minnesota State University - Moorhead
◯ Minot State University
◯ Miriam Hospital
◯ Misericordia University
◯ Mississippi College
◯ Mississippi Delta Community College
◯ Mississippi Gulf Coast Community College
◯ Mississippi State University
◯ Mississippi University For Women
◯ Mississippi Valley State University
◯ Missouri Southern State University
◯ Missouri State University
◯ Missouri University Of Science And Technology
◯ Missouri Western State University
◯ Moberly Area Community College
◯ Mohave Community College
◯ Mohawk Valley Community College
◯ Molloy College
◯ Monell Chemical Senses Center
◯ Monmouth College
◯ Monmouth University
◯ Monroe Community College
◯ Montana State University

◯ Montana State University - Billings
◯ Montana State University - Bozeman
◯ Montana State University - Havre
◯ Montana Tech Of University Of Montana
◯ Montana University
◯ Montclair State University
◯ Montefiore Medical Center
◯ Monterey College Of Law
◯ Monterey Institute Of International Studies
◯ Monterey Peninsula College
◯ Montgomery College
◯ Montgomery Community College
◯ Montgomery County Community College
◯ Moore College Of Art And Design
◯ Moorpark College
◯ Moraine Valley Community College
◯ Moravian College
◯ Morehead State University
◯ Morehouse College
◯ Morehouse School Of Medicine
◯ Morgan State University
◯ Morgridge Institute For Research
◯ Morris Brown College
◯ Morris College
◯ Mott Community College
◯ Mount Aloysius College
◯ Mount Carmel College Of Nursing
◯ Mount Desert Island Biological Lab
◯ Mount Holyoke College
◯ Mount Hood Community College
◯ Mount Ida College
◯ Mount Mercy University
◯ Mount Sacred Heart College
◯ Mount Saint Mary College - Newburgh
◯ Mount San Jacinto College
◯ Mount Sinai School Of Medicine
◯ Mount St. Mary's College
◯ Mount St. Mary's University
◯ Mountain State University
◯ Mt. San Antonio College
◯ Mt. Wachusett Community College
◯ Muhlenberg College
◯ Murray State College
◯ Murray State University
◯ Muskegon Community College
◯ Muskingum University
◯ Naes College Chicago
◯ Naropa University
◯ Nash Community College
◯ Nashville State Community College
◯ Nassau Community College
◯ Nathan S. Kline Institute For Psych Res
◯ National Biodefense Analysis And Countermeasures Center
◯ National Bureau Of Economic Research
◯ National Center For Atmospheric Research
◯ The National Center on Addiction and Substance Abuse
◯ National College (Bayomon PR)
◯ National College Of Natural Medicine
◯ National Cybersecurity Center Of Excellence
◯ National Defense Research Institute
◯ National Defense University
◯ National Development & Research Institutes
◯ National Disease Research Interchange
◯ National Flight Test Institute
◯ National Jewish Health
◯ National Optical Astronomy Observatory
◯ National Partnership for Environmental Technology Education
◯ National Radio Astronomy Observatory

◯ National Renewable Energy Laboratory
◯ National Security Engineering Center
◯ National Solar Observatory
◯ National Technological University
◯ National University
◯ National University Of Health Sciences
◯ National-Louis University
◯ Navajo Technical College
◯ Naval Postgraduate School
◯ Naval War College
◯ Nazareth College
◯ Nebraska Indian Community College
◯ Nebraska Methodist College
◯ Nebraska Wesleyan University
◯ Nemours Children's Clinic
◯ Neumann University
◯ Neuropsychiatric Research Institute
◯ New College Of Florida
◯ New England College Of Optometry
◯ New England Conservatory
◯ New England Research Institutes
◯ New England School Of Acupuncture
◯ New Jersey City University
◯ New Jersey Institute Of Technology
◯ New Jersey Institute Of Technology
◯ New Mexico Highlands University
◯ New Mexico Institute Of Mining And Technology
◯ New Mexico Junior College
◯ New Mexico Military Institute
◯ New Mexico State University
◯ New Orleans Baptist Theological Seminary
◯ The New School
◯ New York Blood Center
◯ New York Chiropractic College
◯ New York College Of Podiatric Medicine
◯ New York Genome Center
◯ New York Institute Of Technology
◯ New York Institute Of Technology
◯ New York Law School
◯ New York Medical College
◯ New York Structural Biology Center
◯ New York University
◯ New York University School Of Medicine
◯ Newberry College
◯ Newman University
◯ NHTI - Concord's Community College
◯ Niagara County Community College
◯ Niagara University
◯ Nicholls State University
◯ Norfolk State University
◯ Normandale Community College
◯ North American Association of Central Cancer Registries
◯ North Carolina Agricultural and Technical State University
◯ North Carolina Agricultural And Technical State University
◯ North Carolina Central University
◯ North Carolina Community College
◯ North Carolina State University
◯ North Central College
◯ North Central Institute
◯ North Dakota State College Of Science
◯ North Dakota State University
◯ North Florida Community College
◯ North Georgia College & State University
◯ North Harris Montgomery Community College
◯ North Hennepin Community College
◯ North Iowa Area Community College
◯ North Orange County Community College
◯ North Park University

◯ North Shore Community College
◯ Northampton Community College
◯ Northeast Community College
◯ Northeast Iowa Community College
◯ Northeast Louisiana Technical College
◯ Northeast Ohio Medical University
◯ Northeast State Technical Community College
◯ Northeast Texas Community College
◯ Northeast Wisconsin Technical College
◯ Northeastern Illinois University
◯ Northeastern Oklahoma Agricultural & Mechanical College
◯ Northeastern State University
◯ Northeastern University
◯ Northern Arizona University
◯ Northern California Institute for Research and Education
◯ Northern Essex Community College
◯ Northern Illinois University
◯ Northern Kentucky University
◯ Northern Marianas College
◯ Northern Michigan University
◯ Northern New Mexico College
◯ Northern State University
◯ Northern Wyoming Community College
◯ Northland College
◯ Northshore Technical Community College - Greensburg
◯ Northshore University Health System
◯ Northside Hospital Atlanta
◯ Northwest Indian College
◯ Northwest Missouri State University
◯ Northwest Nazarene University
◯ Northwestern College - Orange City
◯ Northwestern Health Sciences University
◯ Northwestern Michigan College
◯ Northwestern State University Of Louisiana
◯ Northwestern University
◯ Norwich University
◯ Notre Dame De Namur University
◯ Notre Dame Of Maryland University
◯ Nova Southeastern University
◯ Novelmed Therapeutics
◯ NYSDOH - New York State Department of Health and Health Research
◯ Oak Crest Institute Of Science
◯ Oak Ridge National Laboratory
◯ Oakland University
◯ Oakton Community College
◯ Oakwood University
◯ Oberlin College
◯ Occidental College
◯ Ocean County College
◯ Ocean State Research Institute
◯ Oglala Lakota College
◯ Ohio Christian University
◯ Ohio College Of Podiatric Medicine
◯ Ohio Dominican University
◯ Ohio Northern University
◯ Ohio State University
◯ Ohio University
◯ Ohio Valley University
◯ Ohio Wesleyan University
◯ Ohlone College
◯ Oklahoma City Community College
◯ Oklahoma City University
◯ Oklahoma Medical Research Foundation
◯ Oklahoma Panhandle State University
◯ Oklahoma State University
◯ Old Dominion University
◯ Olivet Nazarene University
◯ Olivet University

◯ Oral Roberts University
◯ Orange Coast College
◯ Orangeburg-Calhoun Technical College
◯ Oregon Center For Applied Science
◯ Oregon College Of Oriental Medicine
◯ Oregon Graduate Institute Of Science And Engineering
◯ Oregon Health & Science University
◯ Oregon Institute Of Technology
◯ Oregon Research Institute
◯ Oregon State University
◯ Oregon University
◯ Otero Junior College
◯ Otterbein University
◯ Ouachita Baptist University
◯ Ouachita Technical College
◯ Our Lady Of Holy Cross College
◯ Our Lady Of The Lake College
◯ Our Lady Of The Lake University
◯ Pace University
◯ Pacific Institute For Research And Evaluation
◯ Pacific Lutheran University
◯ Pacific Northwest National Laboratory
◯ Pacific Northwest Research Institute
◯ Pacific Northwest University Of Health Sciences
◯ Pacific States University
◯ Pacific Union College
◯ Pacific University
◯ Paine College
◯ Palau Community College
◯ Palm Beach Community College
◯ Palmer College Of Chiropractic - Davenport
◯ Palmer College Of Chiropractic - Florida Campus
◯ Palo Alto University
◯ Palo Alto Veterans Instit For Research
◯ Palomar College
◯ Pamlico Community College
◯ Park Nicollet Institute
◯ Park University
◯ Parkland College
◯ Pasadena City College
◯ Passaic County Community College
◯ Paul Smith's College
◯ Pearl River Community College
◯ Peirce College
◯ Pellissippi State Community College
◯ Peninsula College
◯ Pennsylvania College Of Technology
◯ Pennsylvania Institute Of Technology
◯ Pennsylvania State University
◯ Pennsylvania State University - Altoona
◯ Pennsylvania State University - Beaver
◯ Pennsylvania State University - Berks
◯ Pennsylvania State University - Dunmore
◯ Pennsylvania State University - Erie
◯ Pennsylvania State University - Harrisburg
◯ Pennsylvania State University - Malvern - Great Valley School Of Graduate Professional Studies
◯ Pennsylvania State University - Mckeesport
◯ Pennsylvania State University - University Park And Hershey Medical Center
◯ Pepperdine University
◯ Peralta Community College
◯ Peru State College
◯ Pfeiffer University
◯ Philadelphia College Of Osteopathic Medicine
◯ Philadelphia University
◯ Philander Smith College
◯ Phillips Community College Of The University Of Arkansas
◯ Phoenix College

◯ Piedmont Community College
◯ Piedmont Technical College
◯ Pikes Peak Community College
◯ Pikeville College
◯ Pima Community College
◯ Pine Manor College
◯ Pine Technical College
◯ Pitt Community College
◯ Pittsburg State University
◯ Pitzer College
◯ Plymouth State University
◯ Point Loma Nazarene University
◯ Polk State College
◯ Polytechnic Institute Of New York University
◯ Polytechnic University Of Puerto Rico
◯ Pomona College
◯ Ponce School Of Medicine
◯ Pontifical Catholic University Of Puerto Rico
◯ Portland Community College
◯ Portland State University
◯ Prairie View A&M University
◯ Pratt Institute
◯ Presbyterian College
◯ Prescott College
◯ Presentation College
◯ Prince George's Community College
◯ Princeton Plasma Physics Laboratory
◯ Princeton Theological Seminary
◯ Princeton University
◯ Project Air Force
◯ Proteogenomics Research Institute for Systems Medicine
◯ Providence College
◯ Providence Portland Medical Center
◯ Public Health Institute
◯ Public Health Solutions
◯ Puget Sound Blood Center
◯ Pulaski Technical College
◯ Purdue University
◯ Purdue University - Calumet Campus
◯ Purdue University - North Central
◯ Purdue University - West Lafayette
◯ Queens University Of Charlotte
◯ Quinnipiac University
◯ Quinsigamond Community College
◯ Radford University
◯ Ramapo College Of New Jersey
◯ Rancho Santiago Community College
◯ Rand Corporation
◯ Randolph-Macon College
◯ Raritan Valley Community College
◯ Reading Area Community College
◯ Red Rocks Community College
◯ Reed College
◯ Regenerative Medical Solutions
◯ Regent University
◯ Regis College
◯ Regis University
◯ Rehabilitation Institute Of Chicago
◯ Reid State Technical College
◯ Rend Lake College
◯ Rensselaer Polytechnic Institute
◯ Rensselaer Polytechnic Institute - Troy
◯ Rensselaer Polytechnic University - Hartford
◯ Renton Technical College
◯ Research Institute Nationwide Children's Hosp
◯ Research Institute Of Fox Chase Cancer Center
◯ Rhode Island College
◯ Rhode Island Hospital
◯ Rhode Island School Of Design
◯ Rhodes College

◯ Rice University
◯ Rider University - Lawrenceville
◯ Ridgewater College
◯ Rio Hondo College
◯ Ripon College
◯ River Valley Community College
◯ Riverside Community College - Riverside
◯ Riverside Research Institute
◯ Rivier College
◯ Roane State Community College - Harrisman
◯ Roanoke College
◯ Robert Morris University
◯ Roberts Wesleyan College
◯ Robeson Community College
◯ Rochester College
◯ Rochester General Hospital
◯ Rochester Institute Of Technology
◯ Rock Valley College
◯ Rockefeller University
◯ Rockhurst University
◯ Rockland Community College
◯ Rocky Mountain College
◯ Roger Williams Medical Center
◯ Roger Williams University
◯ Roger Williams University
◯ Rogers State University
◯ Rogue Community College
◯ Rollins College
◯ Roosevelt University
◯ Rosalind Franklin University Of Medicine And Science
◯ Rose State College
◯ Rose-Hulman Institute Of Technology
◯ Roseman University Of Health Sciences
◯ Rosemont College
◯ Roskamp Institute
◯ Roswell Park Cancer Institute Corp
◯ Rowan University
◯ Roxbury Community College
◯ Rush University
◯ Russell Sage College
◯ Rust College
◯ Rutgers Biomedical Health Sciences - Robert Wood Johnson Medical School
◯ Rutgers Biomedical Health Sciences - School Of Public Health
◯ Rutgers Biomedical Health Sciences - Cancer Institute of New Jersey
◯ Rutgers Biomedical Health Sciences - New Jersey Medical School
◯ Rutgers The State University Of New Jersey - Camden
◯ Rutgers The State University Of New Jersey - New Brunswick
◯ Rutgers The State University Of New Jersey - Newark
◯ Sacred Heart University
◯ Saddleback College
◯ Saginaw Chippewa Tribal College
◯ Saginaw Valley State University
◯ Saint Anselm College
◯ Saint Augustine's College
◯ Saint Francis University
◯ Saint John's University - Collegeville
◯ Saint Joseph College - West Hartford
◯ Saint Joseph Mercy Health System
◯ Saint Joseph's College Of Maine
◯ Saint Joseph's University
◯ Saint Leo University
◯ Saint Louis University - Saint Louis
◯ Saint Luke's Hospital
◯ Saint Martin's University
◯ Saint Mary's College Of California

◯ Saint Mary's College - Notre Dame
◯ Saint Mary's University Of Minnesota
◯ Saint Michael's College
◯ Saint Paul's College
◯ Saint Peter's College - Jersey City
◯ Saint Vincent College
◯ Saint Xavier University - Chicago
◯ Salem International University
◯ Salem State University
◯ Salisbury University
◯ Salish Kootenai College
◯ Salk Institute For Biological Studies
◯ Salt Lake Community College
◯ Salus University
◯ Salve Regina University
◯ Sam Houston State University
◯ Samford University
◯ Samuel Merritt University - Oakland
◯ San Bernardino Valley College
◯ San Diego Biomedical Research Institute
◯ San Diego City College
◯ San Diego Mesa College
◯ San Diego State University
◯ San Francisco State University
◯ San Jacinto College
◯ San Joaquin Valley College Inc.
◯ San Jose State University
◯ San Jose - Evergreen Community College
◯ San Juan Bautista School Of Medicine
◯ San Juan College
◯ San Mateo County Community College
◯ Sandia Corp - Sandia National Laboratories
◯ Sandia National Laboratories
◯ Sanford Research / USD
◯ Santa Barbara City College
◯ Santa Clara University
◯ Santa Fe Community College - Gainesville
◯ Santa Fe Community College - Santa Fe
◯ Santa Monica College
◯ Santa Rosa Junior College
◯ Sarah Lawrence College
◯ Savannah River National Laboratory
◯ Savannah State University
◯ Savannah Technical College
◯ Saybrook University
◯ Schepens Eye Research Institute
◯ School Of Professional Psychology At Forest Institute
◯ The School Of The Art Institute Of Chicago
◯ Schoolcraft College
◯ Schreiner University
◯ Science And Technology Policy Institute
◯ Scintillon Institute For Photobiology
◯ Scripps College
◯ Scripps Health
◯ Scripps Research Institute - California
◯ Scripps Research Institute - Florida
◯ Seattle Biomedical Research Institute
◯ Seattle Central Community College
◯ Seattle Children's Hospital
◯ Seattle Institute For Biomedical and Clinical Research
◯ Seattle Pacific University
◯ Seattle University
◯ Seminole State College
◯ Seton Hall University
◯ Seton Hill University
◯ Sewanee The University Of The South
◯ Shasta College
◯ Shaw University
◯ Shawnee Community College

◯ Shawnee State University
◯ Shelby State Community College
◯ Sheldon Jackson College
◯ Shelton State Community College
◯ Shenandoah University
◯ Shepherd University
◯ Shippensburg University Of Pennsylvania
◯ Shoreline Community College
◯ Siena College
◯ Siena Heights University
◯ Sierra College
◯ Sierra Nevada College
◯ Simmons College
◯ Sinclair Community College
◯ Sinte Gleska University
◯ Sisseton Wahpeton College
◯ Sisters Of Charity Medical Center School Of Nursing
◯ Sitting Bull College
◯ Skagit Valley College
◯ Skidaway Institute Of Oceanography
◯ Skidmore College
◯ Slac National Accelerator Laboratory
◯ Slippery Rock University Of Pennsylvania
◯ Sloan-Kettering Institute Cancer Research
◯ Smith College
◯ Smith-Kettlewell Eye Research Institute
◯ Smithsonian Institution
◯ Snead State Community College
◯ Snow College
◯ Software Engineering Institute
◯ Sojourner-Douglass College
◯ Sonoma State University
◯ South Carolina State University
◯ South Central College
◯ South Dakota School Of Mines & Technology
◯ South Dakota State University
◯ South Florida Community College
◯ South Mountain Community College
◯ South Orange County Community College
◯ South Plains College
◯ South Puget Sound Community College
◯ South Suburban College Of Cook County
◯ South Texas College
◯ South Texas College Of Law
◯ Southeast Community College Area
◯ Southeast Missouri State University
◯ Southeastern Baptist Theological Seminary
◯ Southeastern Louisiana University
◯ Southeastern Oklahoma State University
◯ Southeastern University
◯ Southern Arkansas University
◯ Southern Baptist Theological Seminary
◯ Southern California College Of Optometry
◯ Southern California Institute For Research and Education
◯ Southern College Of Optometry
◯ Southern Connecticut State University
◯ Southern Illinois University Edwardsville
◯ Southern Illinois University Sch Of Med
◯ Southern Illinois University
◯ Southern Illinois University - Carbondale
◯ Southern Methodist University
◯ Southern Nazarene University
◯ Southern Nevada Cancer Research Fdn
◯ Southern New Hampshire University
◯ Southern Oregon University
◯ Southern Polytechnic State University
◯ Southern Research Institute
◯ Southern State Community College
◯ Southern University Agricultural Research And Extension Center

◯ Southern University And A&M College
◯ Southern University And A&M College - Baton Rouge
◯ Southern University And A&M College - New Orleans
◯ Southern Utah University
◯ Southern Vermont College
◯ Southwest Florida College
◯ Southwest Research Institute
◯ Southwest Texas Junior College
◯ Southwest Wisconsin Technical College
◯ Southwestern Baptist Theological Seminary
◯ Southwestern College - Chula Vista
◯ Southwestern Community College - Creston LA
◯ Southwestern Indian Polytechnic Institute
◯ Southwestern Michigan College
◯ Southwestern Oklahoma State University
◯ Southwestern Oregon Community College
◯ Southwestern University
◯ Spalding University
◯ Spartanburg Technical College
◯ Spaulding Rehabilitation Hospital
◯ Spectrum Health Hospitals
◯ Spelman College
◯ Spring Hill College
◯ Springfield College
◯ Springfield Technical Community College
◯ Sra International
◯ St. Alphonsus College
◯ St. Ambrose University
◯ St. Augustine Community College
◯ St. Barnabas Medical Center
◯ St. Bonaventure University
◯ St. Catharine College
◯ St. Catherine University
◯ St. Charles County Community College
◯ St. Cloud State University
◯ St. Edward's University
◯ St. Elizabeth College Of Nursing
◯ St. Francis College
◯ St. John Fisher College
◯ St. John's College
◯ St. John's University - New York City
◯ St. Joseph's College
◯ St. Joseph's Hospital And Medical Center
◯ St. Lawrence University
◯ St. Louis Community College
◯ St. Louis University
◯ St. Luke's - Roosevelt Institute For Health Sciences
◯ St. Mary's College Of Maryland
◯ St. Mary's University - San Antonio
◯ St. Norbert College
◯ St. Olaf College
◯ St. Petersburg College
◯ St. Thomas University - Miami Gardens
◯ St. Vincent Catholic Medical Centers Of New York
◯ St. Vladimir's Orthodox Theological Seminary
◯ Stanford University
◯ Stark State College
◯ State Center Community College
◯ State College Of Optometry
◯ State Fair Community College
◯ State University Of New York At Buffalo
◯ State University Of New York At New Paltz
◯ State University Of New York At Stony Brook - Stony Brook
◯ State University Of New York College At Brockport
◯ State University Of New York College At Buffalo
◯ State University Of New York College At Cortland
◯ State University Of New York College At Fredonia
◯ State University Of New York College At Geneseo
◯ State University Of New York College At Old

Westbury
- ◯ State University Of New York College At Oneonta
- ◯ State University Of New York College At Oswego
- ◯ State University Of New York College At Plattsburgh
- ◯ State University Of New York College At Potsdam
- ◯ State University Of New York College Of Agriculture And Technology At Cobleskill
- ◯ State University Of New York College Of Agriculture And Technology At Morrisville
- ◯ State University Of New York College Of Environmental Science And Forestry
- ◯ State University Of New York College Of Optometry
- ◯ State University Of New York College Of Technology - Alfred
- ◯ State University Of New York Empire State College
- ◯ State University Of New York Farmingdale State College
- ◯ State University Of New York Fashion Institute Of Technology
- ◯ State University Of New York Health Science Center At Brooklyn
- ◯ State University Of New York Maritime College
- ◯ State University Of New York Purchase College
- ◯ State University Of New York University
- ◯ State University Of New York University At Albany
- ◯ State University Of New York Upstate Medical University
- ◯ State University Of New York - Binghamton University
- ◯ State University Of New York - Polytechnic Institute
- ◯ Stephen F. Austin State University
- ◯ Stephens College
- ◯ Sterling College (Sterling KS)
- ◯ Stetson University
- ◯ Stevens Institute Of Technology
- ◯ Stevenson University
- ◯ Stillman College
- ◯ Stone Child College
- ◯ Stonehill College
- ◯ Stowers Institute For Medical Research
- ◯ Strayer University
- ◯ Suffolk County Community College Ammerman Campus
- ◯ Suffolk University
- ◯ Sul Ross State University
- ◯ Sullivan Alliance to Transform the Health Professions
- ◯ Suny Downstate Medical Center
- ◯ Susquehanna University
- ◯ Swarthmore College
- ◯ Swedish Medical Center - First Hill
- ◯ Sweet Briar College
- ◯ Syracuse University
- ◯ Systems And Analyses Center
- ◯ Tacoma Community College
- ◯ Tacoma General Hospital
- ◯ Talladega College
- ◯ Tallahassee Community College
- ◯ Tarleton State University
- ◯ Tarleton University - Central Texas
- ◯ Tarrant County College
- ◯ Taylor University
- ◯ Teachers College - Columbia University
- ◯ Technical College Of The Lowcountry
- ◯ Temple College
- ◯ Temple University
- ◯ Tennessee State University
- ◯ Tennessee Technological University
- ◯ Texas A&M Agrilife Research
- ◯ Texas A&M Health Science Center
- ◯ Texas A&M Health Science Center - Baylor College

Of Dentistry
◯ Texas A&M International University
◯ Texas A&M University Health Science Center
◯ Texas A&M University
◯ Texas A&M University - College Station
◯ Texas A&M University - Galveston
◯ Texas A&M University - Commerce
◯ Texas A&M University - Corpus Christi
◯ Texas A&M University - Kingsville
◯ Texas A&M University - Texarkana
◯ Texas Biomedical Research Institute
◯ Texas Christian University
◯ Texas College
◯ Texas Engineering Experiment Station
◯ Texas Heart Institute
◯ Texas Medical Center Library
◯ Texas Southern University
◯ Texas State Technical College
◯ Texas State University Unallocated
◯ Texas State University - San Marcos
◯ Texas Tech University Health Sciences Center - El Paso
◯ Texas Tech University Health Sciences Center - Lubbock
◯ Texas Tech University
◯ Texas Wesleyan University
◯ Texas Woman's University
◯ Thaddeus Stevens College Of Technology
◯ The Frederick S. Pardee Rand Graduate School
◯ The Mind Research Network
◯ The Richard Stockton College Of New Jersey
◯ Thiel College
◯ Thomas Edison State College
◯ Thomas Jefferson National Accelerator Facility
◯ Thomas Jefferson University
◯ Thomas More College
◯ Three Rivers Community College
◯ Thunderbird School Of Global Management
◯ Tohono O'Odham Community College
◯ Tompkins Cortland Community College
◯ Torrey Pines Institute For Molecular Studies
◯ Tougaloo College
◯ Touro College
◯ Touro University - Vallejo
◯ Towson University
◯ Toyota Technological Institute At Chicago
◯ Translational Genomics Research Inst
◯ Transylvania University
◯ Treasure Valley Community College
◯ Treatment Research Institute
◯ Tri-College University
◯ Tri-County Community College
◯ Tri-County Technical College
◯ Trident Technical College
◯ Trine University
◯ Trinidad State Junior College
◯ Trinity College - Hartford
◯ Trinity University
◯ Trinity Washington University
◯ Troy University
◯ Truckee Meadows Community College
◯ Truman State University
◯ Tufts Medical Center
◯ Tufts University
◯ Tufts University Medford
◯ Tulane University
◯ Tulsa Community College
◯ Turtle Mountain Community College
◯ Tusculum College
◯ Tuskegee University
◯ Tyler Junior College

◯ U.S. Walter Reed Army Institute Of Research
◯ Umpqua Community College
◯ Uniformed Services University Of The Health Sciences
◯ Union College - Lincoln
◯ Union County College
◯ Union Graduate College
◯ Union Institute & University
◯ Union Presbyterian Seminary - Richmond
◯ Union Theological Seminary
◯ Union University - Jackson
◯ United States Air Force Academy
◯ United States Coast Guard Academy
◯ United States Merchant Marine Academy
◯ United States Military Academy
◯ United States Naval Academy
◯ United States University
◯ United Tribes Technical College
◯ Unity College
◯ Universidad Adventista De Las Antillas
◯ Universidad Central Del Caribe
◯ Universidad Del Este
◯ Universidad Del Turabo
◯ Universidad Metropolitana
◯ Universities And State Colleges Of Arizona
◯ University College Of San Juan
◯ University Corporation For Atmospheric Research
◯ University Of Akron
◯ University Of Alabama At Birmingham
◯ University Of Alabama In Huntsville
◯ University Of Alabama - Tuscaloosa
◯ University Of Alabama
◯ University Of Alaska Anchorage
◯ University Of Alaska Fairbanks
◯ University Of Alaska Southeast
◯ University Of Alaska
◯ University Of Arizona
◯ University Of Arkansas At Little Rock
◯ University Of Arkansas At Monticello
◯ University Of Arkansas For Medical Sciences
◯ University Of Arkansas Pine Bluff
◯ University Of Arkansas
◯ University Of Arkansas - Fayetteville
◯ University Of Arkansas - Ft. Smith
◯ University Of Baltimore
◯ University Of Bridgeport
◯ University Of California Hastings College Of Law
◯ University Of California - Berkeley
◯ University Of California - Cooperative Extension
◯ University Of California - Davis
◯ University Of California - Irvine
◯ University Of California - Los Angeles
◯ University Of California - Merced
◯ University Of California - Riverside
◯ University Of California - San Diego
◯ University Of California - San Francisco
◯ University Of California - Santa Barbara
◯ University Of California - Santa Cruz
◯ University Of Central Arkansas
◯ University Of Central Florida
◯ University Of Central Missouri
◯ University Of Central Oklahoma
◯ University Of Charleston
◯ University Of Chicago
◯ University Of Cincinnati
◯ University Of Colorado
◯ University Of Colorado Boulder
◯ University Of Colorado Colorado Springs
◯ University Of Colorado Denver And Anschutz Medical Campus
◯ University Of Connecticut

◯ University Of Dallas
◯ University Of Dayton
◯ University Of Delaware
◯ University Of Denver
◯ University Of Detroit Mercy
◯ University Of Dubuque
◯ University Of Evansville
◯ University Of Findlay
◯ University Of Florida
◯ University Of Georgia
◯ University Of Great Falls
◯ University Of Guam
◯ University Of Hartford
◯ University Of Hawaii At Hilo
◯ University Of Hawaii At Manoa
◯ University Of Hawaii At West Oahu
◯ University Of Hawaii
◯ University Of Houston
◯ University Of Houston - Clear Lake
◯ University Of Houston - Downtown
◯ University Of Houston - Victoria
◯ University Of Idaho
◯ University Of Illinois At Chicago
◯ University Of Illinois At Springfield
◯ University Of Illinois At Urbana-Champaign
◯ University Of Illinois
◯ University Of Indianapolis
◯ University Of Iowa
◯ University Of Kansas
◯ University Of Kentucky
◯ University Of La Verne
◯ University Of Louisiana At Lafayette
◯ University Of Louisiana At Monroe
◯ University Of Louisiana
◯ University Of Louisville
◯ University Of Maine
◯ University Of Maine At Augusta
◯ University Of Maine At Machias
◯ University Of Maine At Presque Isle
◯ University Of Mary
◯ University Of Mary Washington
◯ University Of Maryland Baltimore
◯ University Of Maryland Biotechnology Institute
◯ University Of Maryland Center For Environmental Science
◯ University Of Maryland Eastern Shore
◯ University Of Maryland
◯ University Of Maryland University College
◯ University Of Maryland - Baltimore County
◯ University Of Maryland - College Park
◯ University Of Massachusetts Amherst
◯ University Of Massachusetts Boston
◯ University Of Massachusetts Dartmouth
◯ University Of Massachusetts Lowell
◯ University Of Massachusetts Medical School
◯ University Of Massachusetts
◯ University Of Memphis
◯ University Of Miami
◯ University Of Miami School Of Medicine
◯ University Of Michigan
◯ University Of Michigan - Ann Arbor
◯ University Of Michigan - Dearborn
◯ University Of Michigan - Flint
◯ University Of Minnesota
◯ University Of Minnesota - Crookston
◯ University Of Minnesota - Duluth
◯ University Of Minnesota - Minneapolis
◯ University Of Minnesota - Morris
◯ University Of Minnesota - Rochester
◯ University Of Mississippi Medical Center
◯ University Of Mississippi

◯ University Of Mississippi - Jackson - Medical Center
◯ University Of Missouri
◯ University Of Missouri - Columbia
◯ University Of Missouri - Kansas City
◯ University Of Missouri - Saint Louis
◯ University Of Montana Western
◯ University Of Montana - Missoula College
◯ University Of Montana
◯ University Of Montevallo
◯ University Of Nebraska At Kearney
◯ University Of Nebraska At Omaha
◯ University Of Nebraska Medical Center
◯ University Of Nebraska
◯ University Of Nebraska - Lincoln
◯ University Of Nevada - Las Vegas
◯ University Of Nevada - Reno
◯ University Of New England
◯ University Of New Hampshire
◯ University Of New Haven
◯ University Of New Mexico Health Sciences Center
◯ University Of New Mexico
◯ University Of New Orleans
◯ University Of North Alabama
◯ University Of North Carolina At Asheville
◯ University Of North Carolina At Chapel Hill
◯ University Of North Carolina At Charlotte
◯ University Of North Carolina At Greensboro
◯ University Of North Carolina At Pembroke
◯ University Of North Carolina At Wilmington
◯ University Of North Carolina
◯ University Of North Dakota
◯ University Of North Florida
◯ University Of North Texas - Denton
◯ University Of North Texas - Health Science Center
◯ University Of Northern Colorado
◯ University Of Northern Iowa
◯ University Of Notre Dame
◯ University Of Oklahoma Health Sciences Center
◯ University Of Oklahoma
◯ University Of Oregon
◯ University Of Pennsylvania
◯ University Of Phoenix
◯ University Of Pittsburgh
◯ University Of Pittsburgh - Bradford
◯ University Of Pittsburgh - Pittsburgh
◯ University Of Portland
◯ University Of Puerto Rico
◯ University Of Puerto Rico At Aguadilla
◯ University Of Puerto Rico At Arecibo
◯ University Of Puerto Rico At Bayamon
◯ University Of Puerto Rico At Carolina
◯ University Of Puerto Rico At Cayey
◯ University Of Puerto Rico At Humacao
◯ University Of Puerto Rico At Mayaguez
◯ University Of Puerto Rico At Ponce
◯ University Of Puerto Rico At Rio Piedras
◯ University Of Puerto Rico La Montana Regional Colleges
◯ University Of Puerto Rico Rio Piedras
◯ University Of Puerto Rico - Medical Sciences Campus
◯ University Of Puget Sound
◯ University Of Redlands
◯ University Of Rhode Island
◯ University Of Richmond
◯ University Of Rio Grande
◯ University Of Rochester
◯ University Of Sacred Heart
◯ University Of Saint Francis
◯ University Of Saint Mary - Leavenworth
◯ University Of San Diego

◯ University Of San Francisco
◯ University Of Scranton
◯ University Of Sioux Falls
◯ University Of South Alabama
◯ University Of South Carolina
◯ University Of South Carolina - Aiken
◯ University Of South Carolina - Beaufort
◯ University Of South Carolina - Columbia
◯ University Of South Carolina - Spartanburg
◯ University Of South Dakota
◯ University Of South Florida Polytechnic
◯ University Of South Florida Sarasota-Manatee
◯ University Of South Florida St. Petersburg
◯ University Of South Florida - Tampa
◯ University Of Southern California
◯ University Of Southern Indiana
◯ University Of Southern Maine
◯ University Of Southern Mississippi
◯ University Of St. Francis
◯ University Of St. Thomas - Minnesota - Saint Paul
◯ University Of St. Thomas - Houston
◯ University Of Tampa
◯ University Of Tennessee Health Sci Center
◯ University Of Tennessee
◯ University Of Tennessee - Agricultural Institute
◯ University Of Tennessee - Chattanooga
◯ University Of Tennessee - Health Science Center
◯ University Of Tennessee - Knoxville
◯ University Of Tennessee - Martin
◯ University Of Tennessee - Tullahoma - Space Institute
◯ University Of Texas At Arlington
◯ University Of Texas At Austin
◯ University Of Texas At Brownsville
◯ University Of Texas At Dallas
◯ University Of Texas At El Paso
◯ University Of Texas At Permian Basin
◯ University Of Texas At San Antonio
◯ University Of Texas At Tyler
◯ University Of Texas Health Science Center At Houston
◯ University Of Texas Health Science Center At San Antonio
◯ University Of Texas M.D. Anderson Cancer Center
◯ University Of Texas Medical Branch
◯ University Of Texas Southwestern Medical Center
◯ University Of Texas - Pan American
◯ University Of The Cumberlands
◯ University Of The District Of Columbia
◯ University Of The Incarnate Word
◯ University Of The Pacific
◯ University Of The Sciences Philadelphia
◯ University Of The Virgin Islands
◯ University Of The West
◯ University Of Toledo
◯ University Of Toledo - Health Science Campus
◯ University Of Tulsa
◯ University Of Utah
◯ University Of Vermont
◯ University Of Virginia
◯ University Of Virginia College At Wise
◯ University Of Washington - Bothell
◯ University Of Washington - Seattle
◯ University Of Washington - Tacoma
◯ University Of West Alabama
◯ University Of West Florida
◯ University Of West Georgia
◯ University Of Western States
◯ University Of Wisconsin Colleges
◯ University Of Wisconsin
◯ University Of Wisconsin - Eau Claire

◯ University Of Wisconsin - Green Bay
◯ University Of Wisconsin - La Crosse
◯ University Of Wisconsin - Madison
◯ University Of Wisconsin - Milwaukee
◯ University Of Wisconsin - Oshkosh
◯ University Of Wisconsin - Parkside
◯ University Of Wisconsin - Platteville
◯ University Of Wisconsin - River Falls
◯ University Of Wisconsin - Stevens Point
◯ University Of Wisconsin - Stout
◯ University Of Wisconsin - Superior
◯ University Of Wisconsin - Whitewater
◯ University Of Wyoming
◯ Urban College Of Boston
◯ Ursinus College
◯ Ursuline College
◯ Utah State University
◯ Utah State University - Price - College Of Eastern Utah
◯ Utah Valley University
◯ Ut-Battelle - Oak Ridge National Lab
◯ Utica College
◯ Valdosta State University
◯ Valencia Community College
◯ Valley City State University
◯ Valparaiso University
◯ Van Andel Research Institute
◯ Vanderbilt University
◯ Vanguard University
◯ Vassar College
◯ Ventura County Community College
◯ Vermilion Community College
◯ Vermont Technical College
◯ Via Christi Regional Medical Center
◯ Villanova University
◯ Vincennes University
◯ Virginia College - Lynchburg
◯ Virginia Commonwealth University
◯ Virginia Community College
◯ Virginia Institute Of Marine Science
◯ Virginia Military Institute
◯ Virginia Polytechnic Institute And State University
◯ Virginia State University
◯ Virginia Union University
◯ Virginia Wesleyan College
◯ Viterbo University
◯ Voorhees College
◯ Wabash College
◯ Wadsworth Center
◯ Wagner College
◯ Wake Forest University
◯ Wake Forest University Health Sciences
◯ Wake Technical Community College
◯ Walden University
◯ Waldorf College
◯ Walla Walla Community College
◯ Walla Walla University
◯ Wallace Community College - Dothan
◯ Wallace State Community College - Selma
◯ Wallace State Community College -Hanceville
◯ Walsh College Of Accountancy And Business Administration
◯ Walsh University
◯ Warren Wilson College
◯ Washburn University
◯ Washington & Jefferson College
◯ Washington And Lee University
◯ Washington College
◯ Washington State Community College
◯ Washington State University
◯ Washington University In St. Louis

◯ Waubonsee Community College
◯ Waukesha County Technical College
◯ Wayland Baptist University
◯ Wayne Community College
◯ Wayne State College
◯ Wayne State University
◯ Waynesburg University
◯ Weatherford College
◯ Webb Institute
◯ Weber State University
◯ Webster University - Saint Louis
◯ Weill Medical Coll Of Cornell Univ
◯ Wellesley College
◯ Wenatchee Valley College
◯ Wentworth Institute Of Technology
◯ Wesley College
◯ Wesleyan College
◯ Wesleyan University
◯ West Chester University Of Pennsylvania
◯ West Georgia Technical College
◯ West Hills Community College
◯ West Liberty University
◯ West Los Angeles College
◯ West Shore Community College
◯ West Suburban College Of Nursing
◯ West Texas A&M University
◯ West Virginia School Of Osteopathic Medicine
◯ West Virginia State University
◯ West Virginia University Institute Of Technology
◯ West Virginia University
◯ West Virginia Wesleyan College
◯ Western Carolina University
◯ Western Connecticut State University
◯ Western Illinois University
◯ Western Iowa Tech Community College
◯ Western Kentucky University
◯ Western Michigan University
◯ Western Nebraska Community College
◯ Western New England College
◯ Western New Mexico University
◯ Western Oklahoma State College
◯ Western Oregon University
◯ Western State College Of Colorado
◯ Western Texas College
◯ Western University Of Health Sciences
◯ Western Washington University
◯ Westfield State University
◯ Westminster College - Fulton
◯ Westminster College - New Wilmington
◯ Westminster College - Salt Lake City
◯ Westminster Theological Seminary
◯ Westmont College
◯ Wharton County Junior College
◯ Whatcom Community College
◯ Wheaton College - Norton
◯ Wheaton College - Wheaton
◯ Wheeling Jesuit University
◯ Wheelock College
◯ White Earth Tribal & Community College
◯ Whitehead Institute For Biomedical Res
◯ Whitman College
◯ Whittier College
◯ Whitworth University
◯ Wichita State University
◯ Widener University
◯ Wilberforce University
◯ Wiley College
◯ Wilkes Community College
◯ Wilkes University
◯ Willamette University
◯ William Beaumont Hospital Research Institute

◯ William Carey University - Hattiesburg
◯ William Jewell College
◯ William Mitchell College Of Law
◯ William Paterson University
◯ Williams College
◯ Wilmington College Of Ohio
◯ Wilmington University
◯ Wilson College
◯ Winifred Masterson Burke Med Research Institute
◯ Winona State University
◯ Winston-Salem State University
◯ Winthrop University
◯ Wisconsin Lutheran College
◯ Wisconsin Technical College
◯ Wistar Institute
◯ Wittenberg University
◯ Wofford College
◯ Wolford College
◯ Women And Infants Hospital - Rhode Island
◯ Woods Hole Oceanographic Institution
◯ Worcester Polytechnic Institute
◯ Worcester State College
◯ Wright Institute
◯ Wright State University
◯ Wyotech
◯ Xavier University
◯ Xavier University Of Louisiana
◯ Yakima Valley Community College
◯ Yale University
◯ Yavapai College
◯ Yeshiva University
◯ York College - Nebraska
◯ York College of Pennsylvania
◯ York Technical College
◯ Yosemite Community College
◯ Youngstown State University
(If your institution or university is not listed, please select "Other" and you will be able to write in your choice.)

If your institution or university is not listed, please write it here.

____

Please choose a primary field that best matches your current position.

◯ Environmental Sciences
◯ Engineering
◯ Humanities
◯ Life Sciences
◯ Medicine
◯ Professions (other)
◯ Physical Sciences
◯ Psychology
◯ Social Sciences
◯ Other (not listed)
(Please choose the best match)

If your field is not listed, please write it here.

____

Please choose a secondary field that best matches your current position.

- ◯ Aeronautics
- ◯ Agriculture
- ◯ Allergy
- ◯ Allied Health
- ◯ Analytical Chemistry
- ◯ Anatomy
- ◯ Anesthesiology
- ◯ Animal Behavior
- ◯ Anthropology
- ◯ Applied Mathematics
- ◯ Applied Physics
- ◯ Archeology
- ◯ Architecture
- ◯ Area Studies
- ◯ Astrobiology
- ◯ Astronautics
- ◯ Astrophysics
- ◯ Atmospheric Science
- ◯ Bacteriology
- ◯ Biochemistry
- ◯ Bioengineering
- ◯ Biogeography
- ◯ Biomechanics
- ◯ Biomedical Science
- ◯ Biomolecular Engineering
- ◯ Biophysics
- ◯ Bioprocess Engineering
- ◯ Biostatistics / Bioinformatics
- ◯ Biotechnology
- ◯ Business
- ◯ Cardiology
- ◯ Cellular Biology
- ◯ Chemical Engineering
- ◯ Chronobiology
- ◯ Civil Engineering
- ◯ Classics
- ◯ Clinical Laboratory Sciences
- ◯ Cognitive Biology
- ◯ Communications
- ◯ Computational Biology
- ◯ Computer Sciences
- ◯ Conservation Biology
- ◯ Construction Engineering
- ◯ Cosmology
- ◯ Criminology
- ◯ Cryobiology
- ◯ Cytometry
- ◯ Dental Surgery
- ◯ Dentistry
- ◯ Dermatology
- ◯ Design
- ◯ Development Studies
- ◯ Divinity
- ◯ Earth Sciences
- ◯ Ecology
- ◯ Economics
- ◯ Education
- ◯ Electrical Engineering
- ◯ Emergency Medicine
- ◯ Endocrinology
- ◯ Environmental Engineering
- ◯ Environmental Management
- ◯ Epidemiology
- ◯ Epigenetics
- ◯ Ethnic and Cultural Studies
- ◯ Evolutionary Biology
- ◯ Family and Consumer Science
- ◯ Film Studies
- ◯ Food Science
- ◯ Gastroenterology

◯ Gender Studies
◯ Genetics
◯ Genomics
◯ Geography
◯ Geoinformatics
◯ Geology
◯ Geotechnical Engineering
◯ Geriatrics
◯ Glycobiology
◯ Hematology
◯ Histology
◯ History
◯ Hospice and Palliative Medicine
◯ Hydraulic Engineering
◯ Immunology
◯ Industrial Engineering
◯ Industry Relations
◯ Infectious Disease
◯ Information Science
◯ Inorganic Chemistry
◯ Internal Medicine
◯ Journalism
◯ Law
◯ Library Studies
◯ Limnology
◯ Linguistics
◯ Literature
◯ Logic
◯ Marine Biology
◯ Materials Science
◯ Mechanical Engineering
◯ Media Studies
◯ Medical Physics
◯ Metallurgical
◯ Meterological
◯ Microbiology
◯ Military Sciences
◯ Molecular Biology
◯ Molecular Engineering
◯ Morphology
◯ Museum Studies
◯ Music
◯ Mycology
◯ Neonatology
◯ Nephrology
◯ Neurology
◯ Neuroscience
◯ Neurosurgery
◯ Nuclear Engineering
◯ Nursing
◯ Nutrition
◯ Obstetrics and Gynecology
◯ Observational Astronomy
◯ Oncology
◯ Ophthalmology
◯ Optics
◯ Optometry
◯ Organic Chemistry
◯ Orthopedics
◯ Other / Interdisciplinary (please specify)
◯ Otorhinolaryngology
◯ Paleobiology
◯ Paleontology
◯ Parasitology
◯ Pathology
◯ Pediatrics
◯ Performing Arts
◯ Petroleum Engineering
◯ Pharmaceuticals
◯ Pharmacology
◯ Pharmacy

◯ Philosophy
◯ Photobiology
◯ Physical Performance
◯ Physical Therapy
◯ Physiology
◯ Planetary Science
◯ Plant Biology
◯ Plastic Surgery
◯ Podiatry
◯ Political Science
◯ Politics
◯ Polymer Engineering
◯ Population Biology
◯ Preventative Medicine
◯ Process Engineering
◯ Programming
◯ Proteomics
◯ Psychiatry
◯ Psychology
◯ Public Administration
◯ Public Health
◯ Public Policy
◯ Pulmonary
◯ Pure Mathematics
◯ Quantum Biology
◯ Quantum Mechanics
◯ Radiology
◯ Rehabilitation Medicine
◯ Religion
◯ Reproductive Biology
◯ Respiratory and Sleep Medicine
◯ Rheumatology
◯ Safety
◯ Social Work
◯ Sociology
◯ Software
◯ Soil Sciences
◯ Space Sciences
◯ Speech and Language Pathology
◯ Statistics
◯ Structural Biology
◯ Surgery
◯ Synthetic Biology
◯ Systematics
◯ Systems Science
◯ Textile Engineering
◯ Theater
◯ Theoretical Astronomy
◯ Theoretical Physics
◯ Toxicology
◯ Translational Science
◯ Transportation
◯ Urology
◯ Vascular Surgery
◯ Veterinary Medicine
◯ Virology
◯ Visual Arts
◯ Xenobiology
◯ Zoology

If your sub-field is not listed, please write it here. ________________________________

Section 2. Professional Development and Structured Supervision (Mentoring)

	Very satisfied	Satisfied	Somewhat satisfied	Not very satisfied	Not at all satisfied	No comment
How satisfied are you with the mentoring that you receive in your laboratory?	◯	◯	◯	◯	◯	◯

	Daily	Weekly	Monthly	Quarterly	Less than quarterly	Other (please specify)
On average, how often do you meet with your mentor to discuss your project? Pick the time frame that most closely fits.	◯	◯	◯	◯	◯	◯

Please specify how often you meet with your mentor ________________________________

	Less than 25	25-35	35-45	45-55	55-65	65-75	Over 75
On average, how many hours per week do you work?	◯	◯	◯	◯	◯	◯	◯

What is the academic rank of your mentor?
- ◯ Assistant Professor
- ◯ Associate Professor
- ◯ Full Professor
- ◯ Emeritus Professor
- ◯ Other (please specify)

Please specify the rank of your supervisor ________________________________

How many people does your PI supervise (select 0, 1, 2, 3, 4, 5, 6, 7+)?

	0	1	2	3	4	5	6	7+
Postdocs or other Ph.D. level scientists	◯	◯	◯	◯	◯	◯	◯	◯
Medical residents or medical students	◯	◯	◯	◯	◯	◯	◯	◯
Graduate students	◯	◯	◯	◯	◯	◯	◯	◯
Technicians	◯	◯	◯	◯	◯	◯	◯	◯
Undergraduates	◯	◯	◯	◯	◯	◯	◯	◯

What is the gender of your supervisor?
- ☐ Male
- ☐ Female

Have you ever received a performance evaluation while working as a postdoc?
- ◯ Yes
- ◯ No

Do you wish to receive an annual performance evaluation?
- ◯ Yes
- ◯ No

Section 3. Grants and Presentations

What is the source of funding that supports your salary (please check all that apply)?

- ☐ Training grant
- ☐ Mentor's grant
- ☐ University or Department grant
- ☐ Have written a grant while a postdoc, but it was not funded or has not yet been reviewed
- ☐ Not sure
- ☐ Individual fellowship

Please specify the type of grant (NIH F32, Beekeeper's Foundation, etc.)

____

How many total publications have you had while working as a postdoc at [institution] (published or in press manuscripts only)?

- ◯ 0
- ◯ 1
- ◯ 2
- ◯ 3
- ◯ 4 or more (please specify)

Please specify the number of publications.

____

How many of these publications are based on work done while at [institution]?

- ◯ 0
- ◯ 1
- ◯ 2
- ◯ 3
- ◯ 4 or more (please specify)

Please specify the number of publications based on your work at your current institution.

____

How many total publications do you have where you are listed as either first author, co-first author, last author or corresponding author (published or in press manuscripts only)?

- ◯ 0
- ◯ 1
- ◯ 2
- ◯ 3
- ◯ 4 or more (please specify)

Please specify the number of publications.

____

Have you presented your work (either poster or oral presentation) at any of the following while at [institution] (please check all that apply)?

- ☐ Lab meetings
- ☐ Departmental seminars
- ☐ Local conferences
- ☐ National Conferences
- ☐ International Conferences
- ☐ As an invited speaker at another institution
- ☐ Other (please specify)

Please specify other venues where you have presented your work.

____

As a postdoc, how many professional conferences have you attended in the last year?

- ◯ 0
- ◯ 1
- ◯ 2
- ◯ 3
- ◯ 4 or more (please specify)

Please specify the number of conferences that you have attended.

____

Section 4. Finances, Cost of Living and Family Structure

What is your current individual gross (pre tax) income?
- ◯ $39001 - $40000
- ◯ $40001 - $42500
- ◯ $42501 - $45000
- ◯ $45001 - $47500
- ◯ $47501 - $50000
- ◯ $50001 - $52500
- ◯ $52501 - $55000
- ◯ Other (please specify)

Please specify your current individual gross (pre tax) income.
____

If you have been a postdoc for more than 1 year, have you received a raise in the past year?
- ◯ Yes
- ◯ No
- ◯ I have been a postdoc for less than 1 year

Are you married or partnered?
- ◯ Yes
- ◯ No

Do your currently live with your spouse or partner?
- ◯ Yes
- ◯ No, my spouse / partner and I are currently separated or in the process of obtaining a divorce
- ◯ No, my spouse / partner is currently living / working in another city / country
- ◯ Other (please specify)

Please specify the living arrangement of your spouse / partner.
____

Please specify the primary reason for this living arrangement with your spouse / partner.
- ◯ High priority on dual careers
- ◯ Restrictions of visa / immigration
- ◯ Short length of postdoc appointment
- ◯ Other (please specify)

Please clarify why your spouse / partner does not presently live with you.
____

Do you rent or own your primary residence?
- ◯ Rent
- ◯ Own
- ◯ Other (please specify)

Please specify your housing arrangement.
____

Approximately how far do you live from campus?
- ◯ Less than 2 miles
- ◯ 2 to 5 miles
- ◯ 5 to 10 miles
- ◯ More than 10 miles (please specify)

Please specify approximately how far you live from campus.
____

How do you regularly commute to campus (please check all that apply)?
- ☐ Walk
- ☐ Public transportation (e.g. commuter rail, bus)
- ☐ Car
- ☐ Bicycle
- ☐ Other motorized transport (e.g. motorcycle, scooter)
- ☐ Other non-motorized transport (e.g. skateboard)

Do you have children?
- ◯ Yes
- ◯ No

Do your children live with you?
- ◯ Yes (year-round)
- ◯ Yes (part-time)
- ◯ No
- ◯ Other (please specify)

Please specify the living arrangement of your children. ______________________________

Do you utilize the childcare facility offered by [institution]?
- ◯ Yes
- ◯ No
- ◯ Not applicable

If not, why don't you utilize the on campus child care?
- ◯ Cost too high
- ◯ Didn't sign up in time
- ◯ Application turned down
- ◯ Happy with current care elsewhere
- ◯ I plan to enroll in the future
- ◯ Other (please specify)

Please specify the reason here. ______________________________

Section 5. Career Information

What are your primary long term career plans?
- ◯ Academia (primarily research-based)
- ◯ Academia (primarily teaching-based)
- ◯ Industrial Research
- ◯ Patent law / Tech transfer
- ◯ Science policy
- ◯ Government / Non-profit
- ◯ Consulting
- ◯ Science writing / Publishing
- ◯ Research Administration
- ◯ Other (please specify)

Please specify your long term career plan. ______________________________

	Very confident	Confident	Somewhat confident	Not very confident	Not at all confident	Not sure
How confident are you that you will attain your specified career plan?	◯	◯	◯	◯	◯	◯

Have your career plans changed since starting your postdoctoral position?
- ◯ Yes
- ◯ No
- ◯ Somewhat
- ◯ No definite plans

(If yes or somewhat, please specify)

If your career plans have changed, what is the primary reason for this change?
- ◯ Salary
- ◯ Difficulty in obtaining desired position(s)
- ◯ Geographic constraints
- ◯ Balancing family and career
- ◯ Insufficient job security
- ◯ Peer pressure
- ◯ Change in career focus
- ◯ Not applicable
- ◯ Other (please specify)

Please specify the primary reason for your career plan change. ______________________________

To this point, how well do you feel your postdoctoral training is preparing you for your specified career plan?

- ◯ Very well
- ◯ Well
- ◯ Indifferent
- ◯ Not very well
- ◯ Not well at all
- ◯ Not sure
- ◯ I have not been in my postdoc long enough to judge

How often do you meet with your advisor to discuss career goals and your progress towards meeting them? Pick the time frame that most closely fits.

- ◯ Quarterly
- ◯ Once a year
- ◯ Less than once a year
- ◯ More than quarterly
- ◯ I have not met with my advisor to discuss my career plans

How supportive of your career plan is your advisor?

- ◯ Very supportive
- ◯ Supportive
- ◯ Somewhat supportive
- ◯ Not supportive
- ◯ My advisor is not aware of my career goals

	Excellent	Good	Fair	Poor	Not sure
What is your perception of the job market in academia in your field?	◯	◯	◯	◯	◯
What is your perception of the job market outside of academia in your field?	◯	◯	◯	◯	◯

Are you currently looking for a permanent position?

- ◯ No
- ◯ Yes, but not very seriously
- ◯ Yes, and very seriously
- ◯ I prefer not to say

When did you decide upon a Ph.D. level career path?

- ◯ Elementary or middle (primary) school
- ◯ High (secondary) school
- ◯ University/College
- ◯ Post-Baccalaureate experience

Section 6. Benefits related Issues

Please indicate in which of the following institutional benefit plans you are enrolled (please check all that apply):

- ☐ Medical
- ☐ Dental
- ☐ Vision
- ☐ Life Insurance
- ☐ Voluntary supplemental life insurance
- ☐ Childcare
- ☐ Commuter
- ☐ FSA
- ☐ Retirement
- ☐ Tuition
- ☐ Housing
- ☐ Loan
- ☐ Other (please specify)

Please specify other benefits not listed that you are enrolled in.

____

What insurance arrangement do you and your family have?

- ◯ Individual coverage through institution
- ◯ Dependent coverage through institution on any of the plans
- ◯ Coverage through my spouse or partner's plan
- ◯ Coverage independent of the institution
- ◯ No insurance coverage
- ◯ Other, please specify

Please specify your and/or your family's insurance arrangement. __________________________________

	Excellent	Good	Satisfactory	Unsatisfactory	Not sure
How would you rate your benefits overall?	◯	◯	◯	◯	◯

How much vacation time have you taken in the last year?

- ◯ Less than 1 week
- ◯ 1 to 2 weeks
- ◯ 2 to 3 weeks
- ◯ 3 to 4 weeks
- ◯ More than 4 weeks (please specify)

Please specify the amount of vacation time over the past year. __________________________________

Section 7. Professional Development

What is the approximate number of professional development seminars and workshops that you have attended in the past year?

- ◯ 0
- ◯ 1 - 5
- ◯ 5 - 10
- ◯ 10 - 15
- ◯ More than 15
- ◯ Not sure

Are you satisfied with the professional development offerings from your institution?

- ◯ Yes
- ◯ No

If you are dissatisfied, please specify. __________________________________

Have you received training in grant writing?

- ◯ Yes
- ◯ No

Have you received mentor training?

- ◯ Yes
- ◯ No

Have you received training in pedagogy?

- ◯ Yes
- ◯ No

Are teaching opportunities offered at your institution?

- ◯ Yes, and my duties are primarily teaching
- ◯ Yes, my duties are primarily research, but I also have a significant teaching requirement as part of my position
- ◯ Yes, and I take advantage of occasional opportunities at my institution such as teaching in my mentor's courses
- ◯ Yes, but teaching is completely optional and I am not teaching during this position
- ◯ No, but I have taken advantage of teaching opportunities elsewhere during this position
- ◯ No, and I am not teaching during this position
- ◯ Other (please specify)

Please specify the teaching opportunities available. ________________________________

Section 8. Demographic Information

What is your gender?

- ◯ Male
- ◯ Female

What is your age?

- ◯ 24 or younger
- ◯ 25-29
- ◯ 30-34
- ◯ 35-39
- ◯ 40 or older

Please specify your age. ________________________________

Please select the best match for your postdoctoral position based primarily on the characteristics listed, rather than on your assigned institution-specific title.

- ◯ Postdoctoral Fellow (temporary non-employee position, stipend from fellowship, primarily research duties)
- ◯ Postdoctoral Research Fellow (temporary fellowship stipend with supplemental stipend from mentor, primarily research duties)
- ◯ Postdoctoral Research Scholar (temporary employee with salary derived from mentor's grants, primarily research duties)
- ◯ Postdoctoral Research Associate (temporary primarily research position with teaching requirement of 1-2 courses per year)
- ◯ Postdoctoral Student (temporary research position, title provides tax / loan deferment benefits)
- ◯ Research Associate / Assistant / Scholar (temporary non-faculty researcher, more advanced than initial postdoc)
- ◯ Research / Principal / Senior Scientist (research-only non-faculty track, significant supervisory role, no term limit)
- ◯ Research Staff / Director (technical oversight and management, no term limit)
- ◯ Visiting Scholar / External Researcher (short-term collaborative researcher funded by home institution)
- ◯ Lecturer / Instructor (temporary employee, full teaching load)
- ◯ Guest Scientist (temporary visitor from industry)
- ◯ Adjunct Professor (part-time teaching position, temporary employee)
- ◯ Other (please specify)

Please specify your position. ________________________________

Do you identify with one or more of these categories (please select all that apply)?

- ☐ Hispanic / Latino
- ☐ White / Caucasian
- ☐ Black / African American
- ☐ Asian / Asian American
- ☐ Native American / Alaska Native
- ☐ Pacific Islander / Hawaii Native
- ☐ Underrepresented
- ☐ Disabled
- ☐ LGBTQ
- ☐ Veteran / Active Duty Military
- ☐ Other (please specify)

Please specify the category(s). ________________________________

What is your residency status in the U.S.?

- ◯ U.S. citizen
- ◯ Permanent resident
- ◯ H1B visa
- ◯ J1 visa
- ◯ F1 visa
- ◯ Other (please specify)

Please specify your residency status. ________________________________

What is your country of citizenship?

◯ AD - Andorra
◯ AE - United Arab Emirates
◯ AF - Afghanistan
◯ AG - Antigua and Barbuda
◯ AI - Anguilla
◯ AL - Albania
◯ AM - Armenia
◯ AO - Angola
◯ AQ - Antarctica
◯ AR - Argentina
◯ AS - American Samoa
◯ AT - Austria
◯ AU - Australia
◯ AW - Aruba
◯ AZ - Azerbaijan
◯ BA - Bosnia and Herzegovina
◯ BB - Barbados
◯ BD - Bangladesh
◯ BE - Belgium
◯ BF - Burkina Faso
◯ BG - Bulgaria
◯ BH - Bahrain
◯ BI - Burundi
◯ BJ - Benin
◯ BL - Saint Barthelemy
◯ BM - Bermuda
◯ BN - Brunei
◯ BO - Bolivia
◯ BR - Brazil
◯ BS - Bahamas, The
◯ BT - Bhutan
◯ BV - Bouvet Island
◯ BW - Botswana
◯ BY - Belarus
◯ BZ - Belize
◯ CA - Canada
◯ CC - Cocos (Keeling) Islands
◯ CD - Congo, Democratic Republic of the
◯ CF - Central African Republic
◯ CG - Congo, Republic of the
◯ CH - Switzerland
◯ CI - Cote d'Ivoire
◯ CK - Cook Islands
◯ CL - Chile
◯ CM - Cameroon
◯ CN - China
◯ CO - Colombia
◯ CR - Costa Rica
◯ CU - Cuba
◯ CV - Cape Verde
◯ CW - Curacao
◯ CX - Christmas Island
◯ CY - Cyprus
◯ CZ - Czech Republic
◯ DE - Germany
◯ DJ - Djibouti
◯ DK - Denmark
◯ DM - Dominica
◯ DO - Dominican Republic
◯ DZ - Algeria
◯ EC - Ecuador
◯ EE - Estonia
◯ EG - Egypt
◯ EH - Western Sahara
◯ ER - Eritrea
◯ ES - Spain
◯ ET - Ethiopia
◯ FI - Finland
◯ FJ - Fiji
◯ FK - Falkland Islands (Islas Malvinas)

◯ FM - Micronesia, Federated States of
◯ FO - Faroe Islands
◯ FR - France
◯ FX - France, Metropolitan
◯ GA - Gabon
◯ GB - United Kingdom
◯ GD - Grenada
◯ GE - Georgia
◯ GF - French Guiana
◯ GG - Guernsey
◯ GH - Ghana
◯ GI - Gibraltar
◯ GL - Greenland
◯ GM - Gambia, The
◯ GN - Guinea
◯ GP - Guadeloupe
◯ GQ - Equatorial Guinea
◯ GR - Greece
◯ GS - South Georgia and the Islands
◯ GT - Guatemala
◯ GU - Guam
◯ GW - Guinea-Bissau
◯ GY - Guyana
◯ HK - Hong Kong
◯ HM - Heard Island and McDonald Islands
◯ HN - Honduras
◯ HR - Croatia
◯ HT - Haiti
◯ HU - Hungary
◯ ID - Indonesia
◯ IE - Ireland
◯ IL - Israel
◯ IM - Isle of Man
◯ IN - India
◯ IO - British Indian Ocean Territory
◯ IQ - Iraq
◯ IR - Iran
◯ IS - Iceland
◯ IT - Italy
◯ JE - Jersey
◯ JM - Jamaica
◯ JO - Jordan
◯ JP - Japan
◯ KE - Kenya
◯ KG - Kyrgyzstan
◯ KH - Cambodia
◯ KI - Kiribati
◯ KM - Comoros
◯ KN - Saint Kitts and Nevis
◯ KP - Korea, North
◯ KR - Korea, South
◯ KW - Kuwait
◯ KY - Cayman Islands
◯ KZ - Kazakhstan
◯ LA - Laos
◯ LB - Lebanon
◯ LC - Saint Lucia
◯ LI - Liechtenstein
◯ LK - Sri Lanka
◯ LR - Liberia
◯ LS - Lesotho
◯ LT - Lithuania
◯ LU - Luxembourg
◯ LV - Latvia
◯ LY - Libya
◯ MA - Morocco
◯ MC - Monaco
◯ MD - Moldova
◯ ME - Montenegro
◯ MF - Saint Martin
◯ MG - Madagascar

◯ MH - Marshall Islands
◯ MK - Macedonia
◯ ML - Mali
◯ MM - Burma
◯ MN - Mongolia
◯ MO - Macau
◯ MP - Northern Mariana Islands
◯ MQ - Martinique
◯ MR - Mauritania
◯ MS - Montserrat
◯ MT - Malta
◯ MU - Mauritius
◯ MV - Maldives
◯ MW - Malawi
◯ MX - Mexico
◯ MY - Malaysia
◯ MZ - Mozambique
◯ NA - Namibia
◯ NC - New Caledonia
◯ NE - Niger
◯ NF - Norfolk Island
◯ NG - Nigeria
◯ NI - Nicaragua
◯ NL - Netherlands
◯ NO - Norway
◯ NP - Nepal
◯ NR - Nauru
◯ NU - Niue
◯ NZ - New Zealand
◯ OM - Oman
◯ PA - Panama
◯ PE - Peru
◯ PF - French Polynesia
◯ PG - Papua New Guinea
◯ PH - Philippines
◯ PK - Pakistan
◯ PL - Poland
◯ PM - Saint Pierre and Miquelon
◯ PN - Pitcairn Islands
◯ PR - Puerto Rico
◯ PS - Gaza Strip
◯ PS - West Bank
◯ PT - Portugal
◯ PW - Palau
◯ PY - Paraguay
◯ QA - Qatar
◯ RE - Reunion
◯ RO - Romania
◯ RS - Serbia
◯ RU - Russia
◯ RW - Rwanda
◯ SA - Saudi Arabia
◯ SB - Solomon Islands
◯ SC - Seychelles
◯ SD - Sudan
◯ SE - Sweden
◯ SG - Singapore
◯ SH - Saint Helena, Ascension, and Tristan da Cunha
◯ SI - Slovenia
◯ SJ - Svalbard
◯ SK - Slovakia
◯ SL - Sierra Leone
◯ SM - San Marino
◯ SN - Senegal
◯ SO - Somalia
◯ SR - Suriname
◯ SS - South Sudan
◯ ST - Sao Tome and Principe
◯ SV - El Salvador
◯ SX - Sint Maarten
◯ SY - Syria

◯ SZ - Swaziland
◯ TC - Turks and Caicos Islands
◯ TD - Chad
◯ TF - French Southern and Antarctic Lands
◯ TG - Togo
◯ TH - Thailand
◯ TJ - Tajikistan
◯ TK - Tokelau
◯ TL - Timor-Leste
◯ TM - Turkmenistan
◯ TN - Tunisia
◯ TO - Tonga
◯ TR - Turkey
◯ TT - Trinidad and Tobago
◯ TV - Tuvalu
◯ TW - Taiwan
◯ TZ - Tanzania
◯ UA - Ukraine
◯ UG - Uganda
◯ UM - United States Minor Outlying Islands
◯ US - United States
◯ UY - Uruguay
◯ UZ - Uzbekistan
◯ VA - Holy See (Vatican City)
◯ VC - Saint Vincent and the Grenadines
◯ VE - Venezuela
◯ VG - British Virgin Islands
◯ VI - Virgin Islands
◯ VN - Vietnam
◯ VU - Vanuatu
◯ WF - Wallis and Futuna
◯ WS - Samoa
◯ XK - Kosovo
◯ YE - Yemen
◯ YT - Mayotte
◯ ZA - South Africa
◯ ZM - Zambia
◯ ZW - Zimbabwe

In what year did you earn your Ph.D. or other doctoral degree?

◯ 2016
◯ 2015
◯ 2014
◯ 2013
◯ 2012
◯ 2011
◯ 2010
◯ 2009 or earlier

Please specify the year you earned your Ph.D.

____

How many years have you been in a postdoctoral position at your institution?

◯ Less than 1 year
◯ 1 to 2 years
◯ 2 to 3 years
◯ 3 to 4 years
◯ 4 to 5 years
◯ More than 5 years

Please specify how many years you have been at your present institution.

____

How many postdoctoral positions have you held prior to your current position (outside of changes to your postdoctoral status)?

◯ 0
◯ 1
◯ 2
◯ 3 or more please specify

Please specify the number of different postdoctoral positions you have had. ________________________________

Please rate the primary reasons why you initially chose to join your mentor's laboratory:

- ◯ Renown of PI (academic reputation)
- ◯ Success of previous trainees
- ◯ Prospects of working with others in the lab
- ◯ Location requirements or access to specific resources
- ◯ Expertise in specific field / topic
- ◯ Other (please specify)

Please specify the primary reason why you choose your mentor's laboratory. ________________________________

Please rate the primary reasons why you initially chose to join your mentor's laboratory: (Please rate in order from most important = 1 to least important = 6)

	1	2	3	4	5	6
Renown of PI (academic reputation)	◯	◯	◯	◯	◯	◯
Success of previous trainees	◯	◯	◯	◯	◯	◯
Prospects of working with others in the lab	◯	◯	◯	◯	◯	◯
Location requirements or access to specific resources	◯	◯	◯	◯	◯	◯
Expertise in specific field / topic	◯	◯	◯	◯	◯	◯
Other (please specify)	◯	◯	◯	◯	◯	◯

Please specify why you chose to join your mentor's laboratory. ________________________________

Where do you get most of your information regarding professional development events?

- ◯ E-mail
- ◯ Website
- ◯ Flyers
- ◯ Word of mouth
- ◯ Other (please specify)

Please specify where you get information regarding professional development events. ________________________________

Do you have a non-peer scientific mentor whom you trust, other than your advisor?

- ◯ Yes
- ◯ No

(If yes, please specify)

If yes, please specify what is the role or title of your non-peer scientific mentor who is not your advisor ________________________________

Did you obtain your Ph.D. in the U.S.?

- ◯ Yes
- ◯ No

(If no, please specify)

In which country did you receive your Ph.D.?

◯ AD - Andorra
◯ AE - United Arab Emirates
◯ AF - Afghanistan
◯ AG - Antigua and Barbuda
◯ AI - Anguilla
◯ AL - Albania
◯ AM - Armenia
◯ AO - Angola
◯ AQ - Antarctica
◯ AR - Argentina
◯ AS - American Samoa
◯ AT - Austria
◯ AU - Australia
◯ AW - Aruba
◯ AZ - Azerbaijan
◯ BA - Bosnia and Herzegovina
◯ BB - Barbados
◯ BD - Bangladesh
◯ BE - Belgium
◯ BF - Burkina Faso
◯ BG - Bulgaria
◯ BH - Bahrain
◯ BI - Burundi
◯ BJ - Benin
◯ BL - Saint Barthelemy
◯ BM - Bermuda
◯ BN - Brunei
◯ BO - Bolivia
◯ BR - Brazil
◯ BS - Bahamas, The
◯ BT - Bhutan
◯ BV - Bouvet Island
◯ BW - Botswana
◯ BY - Belarus
◯ BZ - Belize
◯ CA - Canada
◯ CC - Cocos (Keeling) Islands
◯ CD - Congo, Democratic Republic of the
◯ CF - Central African Republic
◯ CG - Congo, Republic of the
◯ CH - Switzerland
◯ CI - Cote d'Ivoire
◯ CK - Cook Islands
◯ CL - Chile
◯ CM - Cameroon
◯ CN - China
◯ CO - Colombia
◯ CR - Costa Rica
◯ CU - Cuba
◯ CV - Cape Verde
◯ CW - Curacao
◯ CX - Christmas Island
◯ CY - Cyprus
◯ CZ - Czech Republic
◯ DE - Germany
◯ DJ - Djibouti
◯ DK - Denmark
◯ DM - Dominica
◯ DO - Dominican Republic
◯ DZ - Algeria
◯ EC - Ecuador
◯ EE - Estonia
◯ EG - Egypt
◯ EH - Western Sahara
◯ ER - Eritrea
◯ ES - Spain
◯ ET - Ethiopia
◯ FI - Finland
◯ FJ - Fiji
◯ FK - Falkland Islands (Islas Malvinas)

◯ FM - Micronesia, Federated States of
◯ FO - Faroe Islands
◯ FR - France
◯ FX - France, Metropolitan
◯ GA - Gabon
◯ GB - United Kingdom
◯ GD - Grenada
◯ GE - Georgia
◯ GF - French Guiana
◯ GG - Guernsey
◯ GH - Ghana
◯ GI - Gibraltar
◯ GL - Greenland
◯ GM - Gambia, The
◯ GN - Guinea
◯ GP - Guadeloupe
◯ GQ - Equatorial Guinea
◯ GR - Greece
◯ GS - South Georgia and the Islands
◯ GT - Guatemala
◯ GU - Guam
◯ GW - Guinea-Bissau
◯ GY - Guyana
◯ HK - Hong Kong
◯ HM - Heard Island and McDonald Islands
◯ HN - Honduras
◯ HR - Croatia
◯ HT - Haiti
◯ HU - Hungary
◯ ID - Indonesia
◯ IE - Ireland
◯ IL - Israel
◯ IM - Isle of Man
◯ IN - India
◯ IO - British Indian Ocean Territory
◯ IQ - Iraq
◯ IR - Iran
◯ IS - Iceland
◯ IT - Italy
◯ JE - Jersey
◯ JM - Jamaica
◯ JO - Jordan
◯ JP - Japan
◯ KE - Kenya
◯ KG - Kyrgyzstan
◯ KH - Cambodia
◯ KI - Kiribati
◯ KM - Comoros
◯ KN - Saint Kitts and Nevis
◯ KP - Korea, North
◯ KR - Korea, South
◯ KW - Kuwait
◯ KY - Cayman Islands
◯ KZ - Kazakhstan
◯ LA - Laos
◯ LB - Lebanon
◯ LC - Saint Lucia
◯ LI - Liechtenstein
◯ LK - Sri Lanka
◯ LR - Liberia
◯ LS - Lesotho
◯ LT - Lithuania
◯ LU - Luxembourg
◯ LV - Latvia
◯ LY - Libya
◯ MA - Morocco
◯ MC - Monaco
◯ MD - Moldova
◯ ME - Montenegro
◯ MF - Saint Martin
◯ MG - Madagascar

◯ MH - Marshall Islands
◯ MK - Macedonia
◯ ML - Mali
◯ MM - Burma
◯ MN - Mongolia
◯ MO - Macau
◯ MP - Northern Mariana Islands
◯ MQ - Martinique
◯ MR - Mauritania
◯ MS - Montserrat
◯ MT - Malta
◯ MU - Mauritius
◯ MV - Maldives
◯ MW - Malawi
◯ MX - Mexico
◯ MY - Malaysia
◯ MZ - Mozambique
◯ NA - Namibia
◯ NC - New Caledonia
◯ NE - Niger
◯ NF - Norfolk Island
◯ NG - Nigeria
◯ NI - Nicaragua
◯ NL - Netherlands
◯ NO - Norway
◯ NP - Nepal
◯ NR - Nauru
◯ NU - Niue
◯ NZ - New Zealand
◯ OM - Oman
◯ PA - Panama
◯ PE - Peru
◯ PF - French Polynesia
◯ PG - Papua New Guinea
◯ PH - Philippines
◯ PK - Pakistan
◯ PL - Poland
◯ PM - Saint Pierre and Miquelon
◯ PN - Pitcairn Islands
◯ PR - Puerto Rico
◯ PS - Gaza Strip
◯ PS - West Bank
◯ PT - Portugal
◯ PW - Palau
◯ PY - Paraguay
◯ QA - Qatar
◯ RE - Reunion
◯ RO - Romania
◯ RS - Serbia
◯ RU - Russia
◯ RW - Rwanda
◯ SA - Saudi Arabia
◯ SB - Solomon Islands
◯ SC - Seychelles
◯ SD - Sudan
◯ SE - Sweden
◯ SG - Singapore
◯ SH - Saint Helena, Ascension, and Tristan da Cunha
◯ SI - Slovenia
◯ SJ - Svalbard
◯ SK - Slovakia
◯ SL - Sierra Leone
◯ SM - San Marino
◯ SN - Senegal
◯ SO - Somalia
◯ SR - Suriname
◯ SS - South Sudan
◯ ST - Sao Tome and Principe
◯ SV - El Salvador
◯ SX - Sint Maarten
◯ SY - Syria

◯ SZ - Swaziland
◯ TC - Turks and Caicos Islands
◯ TD - Chad
◯ TF - French Southern and Antarctic Lands
◯ TG - Togo
◯ TH - Thailand
◯ TJ - Tajikistan
◯ TK - Tokelau
◯ TL - Timor-Leste
◯ TM - Turkmenistan
◯ TN - Tunisia
◯ TO - Tonga
◯ TR - Turkey
◯ TT - Trinidad and Tobago
◯ TV - Tuvalu
◯ TW - Taiwan
◯ TZ - Tanzania
◯ UA - Ukraine
◯ UG - Uganda
◯ UM - United States Minor Outlying Islands
◯ UY - Uruguay
◯ UZ - Uzbekistan
◯ VA - Holy See (Vatican City)
◯ VC - Saint Vincent and the Grenadines
◯ VE - Venezuela
◯ VG - British Virgin Islands
◯ VI - Virgin Islands
◯ VN - Vietnam
◯ VU - Vanuatu
◯ WF - Wallis and Futuna
◯ WS - Samoa
◯ XK - Kosovo
◯ YE - Yemen
◯ YT - Mayotte
◯ ZA - South Africa
◯ ZM - Zambia
◯ ZW - Zimbabwe

Is your spouse or partner employed?

◯ Yes
◯ No
◯ Not applicable
(If yes, please specify position)

Please specify what your spouse or partner's position is.

____

What is the primary reason you chose to take a postdoctoral position?

◯ To gain additional training in your area
◯ To gain training in a different area
◯ You feel it is a necessary step to obtain a desired permanent position
◯ You were unable to find a different position
◯ Other (please specify)

Please specify the primary reason you chose to take a postdoc position.

____

What do you believe are the most important aspects of a successful postdoc-PI experience? (Please rank in order from most important = 1 to least important = 8)

	1	2	3	4	5	6	7	8
Plenty of time to discuss your work	○	○	○	○	○	○	○	○
Listening to and respecting one another	○	○	○	○	○	○	○	○
Close personal rapport	○	○	○	○	○	○	○	○
Relaxed and comfortable work atmosphere	○	○	○	○	○	○	○	○
Freedom to become an independent investigator	○	○	○	○	○	○	○	○
High profile publications	○	○	○	○	○	○	○	○
High quality research from the lab	○	○	○	○	○	○	○	○
PI well respected in his/her field	○	○	○	○	○	○	○	○

The majority of your training in each of the following areas comes from . . .

For each area below select one of the following options:

	Yourself	Other faculty	Collab-orators	Lab members	PI / Mentor	Other / Combination	NA / No training
Lab techniques	☐	☐	☐	☐	☐	☐	☐
Lab management	☐	☐	☐	☐	☐	☐	☐
Experimental design	☐	☐	☐	☐	☐	☐	☐
Writing / Publishing	☐	☐	☐	☐	☐	☐	☐
Intellectual / Scientific reasoning	☐	☐	☐	☐	☐	☐	☐
Professional skills	☐	☐	☐	☐	☐	☐	☐
Mentoring	☐	☐	☐	☐	☐	☐	☐
Grant writing	☐	☐	☐	☐	☐	☐	☐

Do you plan to pursue a career in the U.S., if possible?

○ Yes
○ No
○ Not sure
(If no, please specify country of choice)

Please specify the country where you plan to pursue your career.

◯ AD - Andorra
◯ AE - United Arab Emirates
◯ AF - Afghanistan
◯ AG - Antigua and Barbuda
◯ AI - Anguilla
◯ AL - Albania
◯ AM - Armenia
◯ AO - Angola
◯ AQ - Antarctica
◯ AR - Argentina
◯ AS - American Samoa
◯ AT - Austria
◯ AU - Australia
◯ AW - Aruba
◯ AZ - Azerbaijan
◯ BA - Bosnia and Herzegovina
◯ BB - Barbados
◯ BD - Bangladesh
◯ BE - Belgium
◯ BF - Burkina Faso
◯ BG - Bulgaria
◯ BH - Bahrain
◯ BI - Burundi
◯ BJ - Benin
◯ BL - Saint Barthelemy
◯ BM - Bermuda
◯ BN - Brunei
◯ BO - Bolivia
◯ BR - Brazil
◯ BS - Bahamas, The
◯ BT - Bhutan
◯ BV - Bouvet Island
◯ BW - Botswana
◯ BY - Belarus
◯ BZ - Belize
◯ CA - Canada
◯ CC - Cocos (Keeling) Islands
◯ CD - Congo, Democratic Republic of the
◯ CF - Central African Republic
◯ CG - Congo, Republic of the
◯ CH - Switzerland
◯ CI - Cote d'Ivoire
◯ CK - Cook Islands
◯ CL - Chile
◯ CM - Cameroon
◯ CN - China
◯ CO - Colombia
◯ CR - Costa Rica
◯ CU - Cuba
◯ CV - Cape Verde
◯ CW - Curacao
◯ CX - Christmas Island
◯ CY - Cyprus
◯ CZ - Czech Republic
◯ DE - Germany
◯ DJ - Djibouti
◯ DK - Denmark
◯ DM - Dominica
◯ DO - Dominican Republic
◯ DZ - Algeria
◯ EC - Ecuador
◯ EE - Estonia
◯ EG - Egypt
◯ EH - Western Sahara
◯ ER - Eritrea
◯ ES - Spain
◯ ET - Ethiopia
◯ FI - Finland
◯ FJ - Fiji
◯ FK - Falkland Islands (Islas Malvinas)

◯ FM - Micronesia, Federated States of
◯ FO - Faroe Islands
◯ FR - France
◯ FX - France, Metropolitan
◯ GA - Gabon
◯ GB - United Kingdom
◯ GD - Grenada
◯ GE - Georgia
◯ GF - French Guiana
◯ GG - Guernsey
◯ GH - Ghana
◯ GI - Gibraltar
◯ GL - Greenland
◯ GM - Gambia, The
◯ GN - Guinea
◯ GP - Guadeloupe
◯ GQ - Equatorial Guinea
◯ GR - Greece
◯ GS - South Georgia and the Islands
◯ GT - Guatemala
◯ GU - Guam
◯ GW - Guinea-Bissau
◯ GY - Guyana
◯ HK - Hong Kong
◯ HM - Heard Island and McDonald Islands
◯ HN - Honduras
◯ HR - Croatia
◯ HT - Haiti
◯ HU - Hungary
◯ ID - Indonesia
◯ IE - Ireland
◯ IL - Israel
◯ IM - Isle of Man
◯ IN - India
◯ IO - British Indian Ocean Territory
◯ IQ - Iraq
◯ IR - Iran
◯ IS - Iceland
◯ IT - Italy
◯ JE - Jersey
◯ JM - Jamaica
◯ JO - Jordan
◯ JP - Japan
◯ KE - Kenya
◯ KG - Kyrgyzstan
◯ KH - Cambodia
◯ KI - Kiribati
◯ KM - Comoros
◯ KN - Saint Kitts and Nevis
◯ KP - Korea, North
◯ KR - Korea, South
◯ KW - Kuwait
◯ KY - Cayman Islands
◯ KZ - Kazakhstan
◯ LA - Laos
◯ LB - Lebanon
◯ LC - Saint Lucia
◯ LI - Liechtenstein
◯ LK - Sri Lanka
◯ LR - Liberia
◯ LS - Lesotho
◯ LT - Lithuania
◯ LU - Luxembourg
◯ LV - Latvia
◯ LY - Libya
◯ MA - Morocco
◯ MC - Monaco
◯ MD - Moldova
◯ ME - Montenegro
◯ MF - Saint Martin
◯ MG - Madagascar

◯ MH - Marshall Islands
◯ MK - Macedonia
◯ ML - Mali
◯ MM - Burma
◯ MN - Mongolia
◯ MO - Macau
◯ MP - Northern Mariana Islands
◯ MQ - Martinique
◯ MR - Mauritania
◯ MS - Montserrat
◯ MT - Malta
◯ MU - Mauritius
◯ MV - Maldives
◯ MW - Malawi
◯ MX - Mexico
◯ MY - Malaysia
◯ MZ - Mozambique
◯ NA - Namibia
◯ NC - New Caledonia
◯ NE - Niger
◯ NF - Norfolk Island
◯ NG - Nigeria
◯ NI - Nicaragua
◯ NL - Netherlands
◯ NO - Norway
◯ NP - Nepal
◯ NR - Nauru
◯ NU - Niue
◯ NZ - New Zealand
◯ OM - Oman
◯ PA - Panama
◯ PE - Peru
◯ PF - French Polynesia
◯ PG - Papua New Guinea
◯ PH - Philippines
◯ PK - Pakistan
◯ PL - Poland
◯ PM - Saint Pierre and Miquelon
◯ PN - Pitcairn Islands
◯ PR - Puerto Rico
◯ PS - Gaza Strip
◯ PS - West Bank
◯ PT - Portugal
◯ PW - Palau
◯ PY - Paraguay
◯ QA - Qatar
◯ RE - Reunion
◯ RO - Romania
◯ RS - Serbia
◯ RU - Russia
◯ RW - Rwanda
◯ SA - Saudi Arabia
◯ SB - Solomon Islands
◯ SC - Seychelles
◯ SD - Sudan
◯ SE - Sweden
◯ SG - Singapore
◯ SH - Saint Helena, Ascension, and Tristan da Cunha
◯ SI - Slovenia
◯ SJ - Svalbard
◯ SK - Slovakia
◯ SL - Sierra Leone
◯ SM - San Marino
◯ SN - Senegal
◯ SO - Somalia
◯ SR - Suriname
◯ SS - South Sudan
◯ ST - Sao Tome and Principe
◯ SV - El Salvador
◯ SX - Sint Maarten
◯ SY - Syria

◯ SZ - Swaziland
◯ TC - Turks and Caicos Islands
◯ TD - Chad
◯ TF - French Southern and Antarctic Lands
◯ TG - Togo
◯ TH - Thailand
◯ TJ - Tajikistan
◯ TK - Tokelau
◯ TL - Timor-Leste
◯ TM - Turkmenistan
◯ TN - Tunisia
◯ TO - Tonga
◯ TR - Turkey
◯ TT - Trinidad and Tobago
◯ TV - Tuvalu
◯ TW - Taiwan
◯ TZ - Tanzania
◯ UA - Ukraine
◯ UG - Uganda
◯ UM - United States Minor Outlying Islands
◯ UY - Uruguay
◯ UZ - Uzbekistan
◯ VA - Holy See (Vatican City)
◯ VC - Saint Vincent and the Grenadines
◯ VE - Venezuela
◯ VG - British Virgin Islands
◯ VI - Virgin Islands
◯ VN - Vietnam
◯ VU - Vanuatu
◯ WF - Wallis and Futuna
◯ WS - Samoa
◯ XK - Kosovo
◯ YE - Yemen
◯ YT - Mayotte
◯ ZA - South Africa
◯ ZM - Zambia
◯ ZW - Zimbabwe

Has funding outlook changed your willingness to pursue an academic research career in the U.S.?

◯ Yes
◯ No
◯ Not sure

During your time as a postdoc, have you participated in any additional training independent from the lab aimed at better preparing you for your specified career plan (e.g. coursework, teaching, etc.)?

◯ Yes
◯ No
(If yes, please specify)

Please specify what additional training you participated in aimed at career preparation.

____

Please rate the importance of each in achieving your career goals:
(Please rate in order from most important = 1 to least important = 8)

	1	2	3	4	5	6	7	8
High-profile publications	◯	◯	◯	◯	◯	◯	◯	◯
High number of publications	◯	◯	◯	◯	◯	◯	◯	◯
Independent research plan	◯	◯	◯	◯	◯	◯	◯	◯
Targeted networking	◯	◯	◯	◯	◯	◯	◯	◯
Additional technical skills / experience	◯	◯	◯	◯	◯	◯	◯	◯
Teaching experience	◯	◯	◯	◯	◯	◯	◯	◯
Professional development	◯	◯	◯	◯	◯	◯	◯	◯
Other (please specify)	◯	◯	◯	◯	◯	◯	◯	◯

Please specify an important skill in achieving your career goals. ______________________________

If you do not achieve your career goals within your desired time-frame, what is your next plan?

◯ Academia (primarily research-based)
◯ Academia (primarily teaching-based)
◯ Industrial Research
◯ Patent law / Tech transfer
◯ Science policy
◯ Government / Non-profit
◯ Consulting
◯ Science writing / Publishing
◯ Research Administration
◯ Other (please specify)

Please specify your next career plan. ______________________________

Please feel free to make any final comments regarding any of the topics covered or not covered in this survey: ______________________________

Almost Finished!

Thank you for participating in the 2016 National Postdoctoral Survey!

By clicking on "Done" you will be given a link to the PRIZE ENTRY website where you will be prompted for your contact information. If you would like to be included in a drawing with a chance to win a $200 travel award to the scientific meeting of your choice, please provide your contact information. This information is not linked in any way to the answers and comments that you have made in the survey.

Again, thank you and good luck!
