# Supplementary material for: United States National Postdoc Survey results and the interaction of gender, career choice and mentor impact
Source: eLife. 2018 Dec 18;7:e40189. doi: 10.7554/eLife.40189 (PMC6298783; doi:10.7554/eLife.40189)
Supplement: Source data 3. [file elife-40189-data3.pdf]

National Postdoc Survey

Review Drafted Changes

The project fields that have been be added, deleted, or modified while in Draft Mode are displayed in the table below so that you may view all drafted changes before they are committed permanently by a REDCap administrator.

RETURN TO PREVIOUS PAGE

Details regarding all changes made in Draft Mode:

- Records in project: **272**
- Fields to be added: **4**
- Fields to be modified: **8**
- Total potentially critical issues: **3**
  - Deleted fields that contain data: **0**
  - Potentially critical issues in modified fields that contain data: **3**
- Total field count BEFORE the changes below are committed: **147**
- Total field count AFTER the changes below are committed: **151**
- **Will these changes be automatically approved? No, an admin will have to review these changes.**

Fields to be ADDED:

- author "How many total publications do you have where you are listed as either first author, co-first author, last author or corresponding author (published o…
- author_specify "Please specify the number of publications."
- demo "Do you identify with one or more of these categories (please select all that apply)? "
- demo_specify "Please specify the category(s)."

Forms to be ADDED: *none*

Fields to be DELETED: *none*

Forms to be DELETED: *none*

KEY for Comparison Table below
White cell = no change
Yellow cell = field changed (Black text = new
Green cell = new project field

Below is a listing of the changes to be committed to this project.

Variable Name	Section Header	Field Type	Field Label	Choices or Calculations	Field Note	Text Validation Type	Text Validation Min	Text Validation Max	Identifier?	Branching Logic	Required Field?	Image/File Attachment	Custom Alignment	Stop Actio
				1, Other - not listed<br>2, A. T. Still University Of Health Sciences<br>3, Aaron Diamond Aids Research Center<br>4, Abilene Christian University<br>5, Abraham Baldwin Agricultural College<br>6, Adams State College<br>7, Adelphi University<br>8, Adrian College<br>9, Aerospace Federally Funded Research And Development Center<br>10, Agnes Scott College<br>11, Aib College Of Business<br>12, Aiken Technical College<br>13, Aims Community College<br>14, Air Force Institute Of Technology<br>15, Alabama A&M University<br>16, Alabama Southern Community College<br>17, Alabama State University<br>18, Alamo Community College<br>19, Alaska Pacific University<br>20, Albany College Of Pharmacy And Health Sciences<br>21, Albany Medical College<br>22, Albany Molecular Research<br>23, Albany State University<br>24, Albert Einstein Healthcare Network<br>25, Albion College<br>26, Albright College<br>27, Alcorn State University<br>28, Alderson-Broaddus College<br>29, Alfred I. Du Pont Hospital For Children<br>30, Alfred University<br>31, Allan Hancock College<br>32, Allegany College Of Maryland<br>33, Allegheny College<br>34, Allegheny University Of The Health Sciences<br>35, Allegheny-Singer Research Institute<br>36, Allen College- Waterloo<br>37, Allen Institute<br>38, Allen University<br>39, Alliant International University<br>40, Alma College<br>41, Alvernia University<br>42, Alverno College<br>43, Amarillo College<br>44, American Academy of Child and Adolescent Psychiatry<br>45, American Association For Cancer Research<br>46, American College Of Medical Genetics<br>47, American College Of Radiology<br>48, American College - Fayetteville<br>49, American International College<br>50, American Samoa										

Community College
51, American Type Culture Collection
52, American University Of Puerto Rico - Bayamon
53, American University
54, Ames Laboratory
55, Amherst College
56, Amridge University
57, Ana G. Mendez University
58, Andrew College
59, Andrew Jackson University
60, Andrews University
61, Angelo State University
62, Anna Maria College
63, Anne Arundel Community College
64, Anoka-Ramsey Community College
65, Antaya Science And Technology
66, Antelope Valley College
67, Antioch University
68, Appalachian State University
69, Arapahoe Community College
70, Arbor Research Collaborative For Health
71, Arcadia University
72, Argonne National Laboratory
73, Argosy University
74, Arizona State University
75, Arizona Western College
76, Arkansas Baptist College
77, Arkansas Children's Hospital Research Institute
78, Arkansas State University
79, Arkansas Tech University
80, Armstrong Atlantic State University
81, Arrowhead Community Colleges
82, Arroyo Center
83, Art Center College Of Design
84, Art Institute Of Seattle
85, Asbury Theological Seminary
86, Asheville-Buncombe Technical Community College
87, Ashland University
88, Assumption College
89, Atenas College
90, Atlanta University Center
91, Atlantic Cape Community College
92, Auburn University - Auburn
93, Auburn University - Montgomery
94, Augsburg College
95, Augusta State University
96, Augustana College - Rock Island
97, Augustana College - Sioux Falls
98, Aurora University
99, Austin College
100, Austin Community College - Northridge Campus
101, Austin Peay State University
102, Avila University
103, Azusa Pacific University
104, Babson College
105, Baker College - Flint
106, Baker University
107, Baldwin-Wallace College
108, Ball State University
109, Baltimore City Community College
110, Bank Street College Of Education
111, Banner Alzheimer's Institute
112, Banner Health
113, Baptist Memorial College Of Health Sciences
114, Bard College
115, Bard College At Simon's Rock
116, Barnard College
117, Barnes-Jewish College Goldfarb School Of Nursing
118, Barry University
119, Barton County Community College
120, Baruch S. Blumberg Institute
121, Bastyr University
122, Bates College
123, Baton Rouge Community College
124, Battelle Centers for Public Health Research and Evaluation
125, Battelle Pacific Northwest Laboratories
126, Bay Area Tumor Institute
127, Bay De Noc Community College
128, Bay Mills Community College
129, Baylor College Of Medicine
130, Baylor Research Institute
131, Baylor University
132, Baystate Medical Center
133, Beaufort County Community College
134, Bellarmine University
135, Bellevue College
136, Bellin College
137, Belmont University
138, Beloit College
139, Bemidji State University

140, Benaroya Research Institute At Virginia Mason
141, Benedict College
142, Benedictine College
143, Benedictine University
144, Benjamin Franklin Institute Of Technology
145, Bennett College For Women
146, Bennington College
147, Bentley University
148, Berea College
149, Bergen Community College
150, Berkeley College - New York City
151, Berry College
152, Bessemer State Technical College
153, Beth Israel Deaconess Medical Center
154, Beth Israel Medical Center (New York)
155, Bethany College - Bethany
156, Bethel College - Mishawaka - IN
157, Bethel College - North Newton
158, Bethel University
159, Bethune-Cookman University
160, Bevill State Community College Walker Campus
161, Biola University
162, Biomedical Research Institute
163, Birmingham Southern College
164, Bishop State Community College
165, Bismarck State College
166, Black Hawk College
167, Black Hills State University
168, Blackfeet Community College
169, Blackhawk Technical College
170, Blood Systems Research Institute
171, Bloodcenter Of Wisconsin
172, Bloomfield College
173, Bloomsburg University Of Pennsylvania
174, Bluefield State College
175, Boise State University
176, Boston Architectural Center
177, Boston College
178, Boston Medical Center
179, Boston University
180, Boston University Medical Campus
181, Bowdoin College
182, Bowie State University
183, Bowling Green State University
184, Bradley University
185, Brandeis University
186, Brazosport College
187, Brenau University
188, Brentwood Biomedical Research Institute
189, Brescia University
190, Brevard Community College
191, Bridgewater College
192, Bridgewater State College
193, Brigham And Women's Hospital
194, Brigham Young University
195, Brigham Young University - Provo
196, Bristol Community College
197, Brite Divinity School
198, Broad Institute
199, Brookdale Community College
200, Brookhaven National Laboratory
201, Brookhaven Science Association - Brookhaven Lab
202, Brooklyn Law School
203, Broward College
204, Brown Mackie College
205, Brown University
206, Brunswick Community College
207, Bryant University
208, Bryn Athyn College
209, Bryn Mawr College
210, Buck Institute For Research On Aging
211, Bucknell University
212, Bucks County Community College
213, Buena Vista University
214, Bunker Hill Community College
215, Burlington County College
216, Butler County Community College (Butler PA)
217, Butler Hospital (Providence RI)
218, Butler University
219, Butte College
220, Cabrillo College
221, Cabrini College
222, Calhoun Community College
223, California Baptist University
224, California College Of Arts And Crafts
225, California Community

College
226, California Institute Of Integral Studies
227, California Institute Of Technology
228, California Institute Of The Arts
229, California Lutheran University
230, California Maritime Academy
231, California Pacific Medical Center Research Institute
232, California Polytechnic State University - San Luis Obispo
233, California School Of Professional Psychology - Berkeley-Alameda
234, California School Of Professional Psychology - Fresno
235, California School Of Professional Psychology - Los Angeles
236, California State Polytechnic University - Pomona
237, California State University Unallocated
238, California State University - Bakersfield
239, California State University - Channel Islands
240, California State University - Chico
241, California State University - Dominguez Hills
242, California State University - East Bay
243, California State University - Fresno
244, California State University - Fullerton
245, California State University - Long Beach
246, California State University - Los Angeles
247, California State University - Monterey Bay
248, California State University - Northridge
249, California State University - Sacramento
250, California State University - San Bernardino
251, California State University - San Marcos
252, California State University - Stanislaus
253, California University Of Pennsylvania
254, Calvin College
255, Calvin Theological Seminary
256, Cambridge Health Alliance
257, Camden County College
258, Cameron University
259, Campbell University
260, Cancer Prevention Institute Of California
261, Canisius College
262, Cankdeska Cikana Community College
263, Cape Cod Community College
264, Cape Fear Community College
265, Capella University
266, Capital University
267, Capitol College
268, Cardinal Stritch University
269, Caribbean University
270, Carl Albert State College
271, Carl Sandburg College
272, Carleton College
273, Carlos Albizu University
274, Carlow University
275, Carnegie Mellon University
276, Carolinas Medical Center
277, Carroll College
278, Carroll University
279, Carson-Newman College
280, Carthage College
281, Case Western Reserve University
282, Casper College
283, Castleton State College
284, Catawba College
285, Catawba Valley Community College
286, Catholic University Of America
287, Cayuga Community College
288, Cedar Crest College
289, Cedars-Sinai Medical Center
290, Centenary College
291, Centenary College Of Louisiana
292, Center For Advanced Aviation System Development
293, Center For Communications And Computing
294, Center For Construction Research And Training
295, Center For Enterprise Modernization
296, Center For Expermental Software Engr Md
297, Center For Innovative Public Health Res
298, Center For Naval Analyses
299, Center For Nuclear Waste Regulatory Analyses

				300, Center For Psychological Consultation<br>301, Center For Social Innovation<br>302, Central Alabama Community College<br>303, Central Arizona College<br>304, Central College<br>305, Central Community College<br>306, Central Connecticut State University<br>307, Central Florida Community College<br>308, Central Georgia Technical College<br>309, Central Lakes College - Brainerd<br>310, Central Louisiana Technical College<br>311, Central Maine Community College<br>312, Central Michigan University<br>313, Central New Mexico Community College<br>314, Central Oregon Community College<br>315, Central Piedmont Community College<br>316, Central State University<br>317, Central Washington University<br>318, Central Wyoming College<br>319, Centralia College<br>320, Centre College<br>321, Centro De Estudios Multidisciplinarios<br>322, Century College<br>323, Cerritos College<br>324, Chadron State College<br>325, Chaminade University Of Honolulu<br>326, Chancellor University<br>327, Chapman University<br>328, Charles R. Drew University Of Medical & Sci<br>329, Charles R. Drew University Of Medicine And Science<br>330, Charles River Laboratories<br>331, Charles Stark Draper Laboratory<br>332, Charleston Southern University<br>333, Chatham University - Pittsburgh<br>334, Chattanooga State Community College<br>335, Chemeketa Community College<br>336, Chesapeake College<br>337, Chestnut Health Systems<br>338, Chestnut Hill College<br>339, Cheyenne River Community College<br>340, Cheyney University Of Pennsylvania<br>341, CHI Institute For Research & Innovation<br>342, Chicago School Of Professional Psychology<br>343, Chicago State University<br>344, Chicago Theological Seminary<br>345, Chief Dull Knife College<br>346, Children's Hospital Of Philadelphia<br>347, Children's Hospital & Research Center At Oakland<br>348, Children's Hospital Corporation<br>349, Children's Hospital Of Los Angeles<br>350, Children's Mercy Hospital (Kansas City MO)<br>351, Children's Research Institute<br>352, Chippewa Valley Technical College<br>353, Chowan University<br>354, Christian Brothers University<br>355, Christopher Newport University<br>356, Cincinnati Childrens Hospital Medical Center<br>357, Cincinnati State Technical And Community College<br>358, Citadel Military College Of South Carolina<br>359, City College Of San Francisco<br>360, City Colleges Of Chicago<br>361, City Of Hope<br>362, City Of Hope / Beckman Research Institute<br>363, City University Of New York Unallocated<br>364, City University Of New York - School of Law<br>365, City University Of New York - Graduate Center<br>366, City University Of New York - Baruch College<br>367, City University Of New York - Borough Of Manhattan Community College<br>368, City University Of New York - Bronx Community College<br>369, City University Of New York - Brooklyn College<br>370, City University Of New York - College Of Staten Island										

371, City University Of New York - Graduate Center
372, City University Of New York - Hostos Community College
373, City University Of New York - Hunter College
374, City University Of New York - John Jay College Criminal Justice
375, City University Of New York - Kingsborough Community College
376, City University Of New York - Laguardia Community College
377, City University Of New York - Lehman College
378, City University Of New York - Medgar Evers College
379, City University Of New York - New York City College Of Technology
380, City University Of New York - Queens College
381, City University Of New York - Queensborough Community College
382, City University Of New York
383, City University Of New York - The City University
384, City University Of New York - York College
385, Clackamas Community College
386, Claflin University
387, Claremont Graduate University
388, Claremont Mckenna College
389, Claremont School Of Theology
390, Clarion University Of Pennsylvania
391, Clark Atlanta University
392, Clark College
393, Clark State Community College
394, Clark University
395, Clarke University
396, Clarkson College
397, Clarkson University
398, Clatsop Community College
399, Clayton State University
400, Cleary University
401, Clemson University
402, Cleveland Clinic Lerner College of Medicine of CWRU
403, Cleveland Community College
404, Cleveland Institute Of Music
405, Cleveland State Community College
406, Cleveland State University
407, Clinton Junior College
408, Cloud County Community College
409, Cms Alliance To Modernize Healthcare
410, Coastal Bend College
411, Coastal Carolina Community College
412, Coastal Carolina University
413, Coastline Community College
414, Cochise College
415, Coe College
416, Coker College
417, Colby College
418, Colby Community College
419, Colby-Sawyer College
420, Cold Spring Harbor Laboratory
421, Colgate University
422, College Of Charleston
423, College Of Dupage
424, College Of Idaho
425, College Of Lake County
426, College Of Menominee Nation
427, College Of Micronesia - FSM
428, College Of Mount Saint Vincent
429, College Of Mount St. Joseph
430, College Of Nanoscale Science And Engineering Of The University At Albany Suny
431, College Of New Jersey
432, College Of New Rochelle
433, College Of Saint Benedict
434, College Of Saint Rose
435, College Of Southern Idaho
436, College Of Southern Maryland
437, College Of Southern Nevada
438, College Of St. Elizabeth
439, College Of St. Mary
440, College Of St. Scholastica - Duluth
441, College Of The Atlantic
442, College Of The Canyons
443, College Of The Holy Cross
444, College Of The Mainland
445, College Of The Sequoias
446, College Of William And Mary
447, College Of Wooster
448, Collin County Community

College
449, Colorado Cancer Research Program
450, Colorado College
451, Colorado Mountain College
452, Colorado School Of Mines
453, Colorado State University
454, Colorado State University - Pueblo
455, Colorado State University
456, Colorado Theological Seminary
457, Columbia Basin College
458, Columbia College Chicago
459, Columbia College - Columbia MO
460, Columbia College - Columbia SC
461, Columbia University New York Morningside
462, Columbia University Health Sciences
463, Columbia University In The City Of New York
464, Columbia University Teachers College
465, Columbus Community Clinical Oncology Program
466, Columbus State Community College
467, Columbus State University
468, Comanche Nation College
469, Commonwealth Medical College
470, Community College Of Allegheny County
471, Community College Of Aurora
472, Community College Of Baltimore County Catonsville
473, Community College Of Philadelphia
474, Community College Of Rhode Island
475, Community Colleges Of Spokane
476, Concord University
477, Concordia College
478, Concordia Seminary
479, Concordia Theological Seminary
480, Concordia University Chicago
481, Concordia University Wisconsin
482, Connecticut Children's Medical Center
483, Connecticut College
484, Connecticut State Dept Of Public Health
485, Connecticut State University
486, Connors State College
487, Contra Costa College
488, Contra Costa Community College
489, Contra Costa Community College - San Ramon - Diablo Valley College
490, Converse College
491, Cooper Institute
492, Cooper Union For The Advancement Of Science And Art
493, Coppin State University
494, Coriell Institute For Medical Research
495, Cornell College
496, Cornell University
497, Corning Community College
498, County College Of Morris
499, Covenant College
500, Cox College Of Nursing And Health Sciences
501, Craven Community College
502, Creighton University
503, Crowder College
504, Crown College
505, Cuyahoga Community College
506, Daemen College
507, Dakota County Technical College
508, Dakota State University
509, Dakota Wesleyan University
510, Dallas Community College - Eastfield College
511, Dallas Community College - Richland College
512, Dallas Theological Seminary
513, Dana-Farber Cancer Institute
514, Dartmouth College
515, Darton College
516, Data Numerica Institute
517, Davidson College
518, Davidson County Community College
519, Davis & Elkins College
520, Dayton Clinical Oncology Program
521, Daytona State College
522, De Anza College
523, Decatur Memorial Hospital
524, Defense Acquisition University
525, Del Mar College
526, Delaware State University
527, Delaware Technical

Community College
528, Delaware Valley College
529, Delgado Community College
530, Delta State University
531, Denison University
532, Denmark Technical College
533, Denver Health And Hospital Authority
534, Depaul University
535, Depauw University
536, Des Moines Area Community College
537, Des Moines University
538, Desales University
539, Desert Research Institute
540, Dickinson College
541, Dickinson State University
542, Dillard University
543, Dine College
544, Dixie State College Of Utah
545, Doane College
546, Doheny Eye Institute
547, Dominican College Of Blauvelt
548, Dominican University
549, Dominican University Of California
550, Donnelly College
551, Dordt College
552, Dowling College
553, D-Q University
554, Drake University
555, Drew University
556, Drexel University
557, Drury University
558, Duke University
559, Duquesne University
560, Durham Technical Community College
561, Dyersburg State Community College
562, D'Youville College
563, Earlham College
564, East Arkansas Community College
565, East Carolina University
566, East Central University
567, East Los Angeles College
568, East Mississippi Community College
569, East Stroudsburg University Of Pennsylvania
570, East Tennessee State University
571, Eastern Arizona College
572, Eastern Connecticut State University
573, Eastern Idaho Technical College
574, Eastern Illinois University
575, Eastern Iowa Community College
576, Eastern Kentucky University
577, Eastern Mennonite University
578, Eastern Michigan University
579, Eastern New Mexico University
580, Eastern Oklahoma State College
581, Eastern Oregon University
582, Eastern Virginia Medical School
583, Eastern Washington University
584, East-West University
585, Eckerd College
586, ECOG-ACRIN Medical Research Foundation
587, Ecpi College Of Technology
588, Edinboro University Of Pennsylvania
589, Edison State College
590, Edison State Community College
591, Edmonds Community College
592, Edward Via College Of Osteopathic Medicine
593, Edward Waters College
594, El Camino College
595, El Camino College Compton Center
596, El Paso Community College
597, Elgin Community College
598, Elizabeth City State University
599, Elizabethtown College
600, Elmhurst College
601, Elms College
602, Elon University
603, Emanuel Hospital And Health Center
604, Embry-Riddle Aeronautical University
605, Emergent Product Development Gaithersbur
606, Emerson College
607, Emma Pendleton Bradley Hospital
608, Emmanuel College
609, Emory & Henry College
610, Emory University
611, Emporia State University
612, Endicott College
613, Erie Community College North Campus
614, Erikson Institute
615, Erskine College

616, Essentia Institute Of Rural Health
617, Essex County College
618, Everett Community College
619, Evergreen State College - Olympia
620, Evergreen Valley College
621, Experimental Pathology Laboratories
622, Fairfield University
623, Fairleigh Dickinson University
624, Fairmont State University
625, Family Health International
626, Fayetteville State University
627, Fayetteville Technical Community College
628, Feinstein Institute For Medical Research
629, Felician College
630, Fermi National Accelerator Laboratory
631, Ferris State University
632, Ferrum College
633, Fielding Graduate University
634, Finger Lakes Community College
635, Finlandia University
636, Fisk University
637, Fitchburg State University
638, Flathead Valley Community College
639, Florence-Darlington Technical College
640, Florida Agricultural And Mechanical University
641, Florida Atlantic University
642, Florida Gulf Coast University
643, Florida Hospital College Of Health Sciences
644, Florida Institute Of Technology
645, Florida International University
646, Florida Keys Community College
647, Florida Memorial University
648, Florida Metropolitan University Tampa Campus
649, Florida Southern College
650, Florida State College At Jacksonville
651, Florida State University
652, Fond Du Lac Tribal And Community College
653, Foothill-De Anza Community College
654, Fordham University
655, Forsyth Institute
656, Forsyth Technical Community College
657, Fort Belknap College
658, Fort Berthold Community College
659, Fort Hays State University
660, Fort Lewis College
661, Fort Peck Community College
662, Fort Valley State University
663, Foundation For Aids Research
664, Fox Chase Chemical Diversity Center
665, Fox Valley Technical College
666, Framingham State College
667, Francis Marion University
668, Franciscan University Of Steubenville
669, Frank Phillips College
670, Franklin & Marshall College
671, Franklin Pierce Law Center
672, Franklin Pierce University - Rindge
673, Franklin W. Olin College Of Engineering
674, Fred Hutchinson Cancer Research Center
675, Frederick National Laboratory For Cancer Research
676, Fresno City College
677, Friends Research Institute
678, Frontier School Of Midwifery And Family Nursing
679, Frostburg State University
680, Fuller Theological Seminary - Pasadena
681, Fulton-Montgomery Community College
682, Furman University
683, Future Generations Graduate School
684, Gadsden State Community College
685, Gallaudet University
686, Gannon University
687, Garrett College
688, Garrett-Evangelical Theological Seminary
689, Gaston College
690, Gateway Community And Technical College
691, Gateway Technical College
692, Geisinger Clinic
693, General Electric Global Research Center
694, Genesee Community College
695, Geophysical Institute -

University of Alaska Fairbanks
696, George Fox University
697, George Mason University
698, George Washington University
699, Georgetown College
700, Georgetown University
701, Georgia College And State University
702, Georgia Health Sciences University
703, Georgia Institute Of Technology
704, Georgia Perimeter College
705, Georgia Regents University
706, Georgia Southern University
707, Georgia Southwestern State University
708, Georgia State University
709, Georgian Court University
710, Gettysburg College
711, Glen Oaks Community College
712, Glendale Community College - Glendale
713, Glenville State College
714, Globe University - Minnesota School Of Business
715, Goddard College
716, Gogebic Community College
717, Golden Gate University
718, Gonzaga University
719, Goodwin College
720, Gordon College
721, Goshen College
722, Goucher College
723, Governors State University
724, Graceland University
725, Graduate School Usa
726, Graduate Theological Union
727, Grambling State University
728, Grand Rapids Community College
729, Grand Valley State University
730, Grand View University
731, Grayson County College
732, Green Mountain College
733, Green River Community College
734, Greenfield Community College
735, Greenville College
736, Greenville Health System
737, Greenville Technical College
738, Grinnell College
739, Grossmont College
740, Grossmont-Cuyamaca Community College
741, Group Health Cooperative
742, Guam Community College
743, Guilford College
744, Gulf Coast Community College
745, Gustavus Adolphus College
746, Gwynedd-Mercy College
747, H. Lee Moffitt Cancer Center & Research Institute
748, Hackensack University Medical Center
749, Hagerstown Community College
750, Hamilton College
751, Hamline University
752, Hampden-Sydney College
753, Hampshire College
754, Hampton University
755, Harcum College
756, Harding University - Searcy
757, Harford Community College
758, Harper College
759, Harrisburg Area Community College
760, Harrisburg University Of Science And Technology
761, Harris-Stowe State University
762, Hartford Hospital
763, Hartnell College
764, Hartwick College
765, Harvard Medical School
766, Harvard Pilgrim Health Care
767, Harvard School Of Public Health
768, Harvard University
769, Harvey Mudd College
770, Haskell Indian Nations University
771, Hauptman-Woodward Medical Research Institute
772, Haverford College
773, Hawaii Pacific University
774, Haywood Community College
775, Healthpartners Institute
776, Heartland Community College
777, Hebrew Union College-Jewish Institute Of Religion
778, Heidelberg University
779, Hektoen Institute For Medical Research
780, Helene Fuld College Of Nursing
781, Henderson State University
782, Hendrix College
783, Henry Ford Community

College
784, Henry Ford Health System
785, Henry M. Jackson Foundation for the Advancement of Military Medicine
786, Heritage University
787, High Point University
788, Highland Community College - Freeport - Il
789, Highland Community College - Highland
790, Highline Community College
791, Hillsborough Community College
792, Hinds Community College
793, Hiram College
794, Hobart And William Smith Colleges
795, Hocking College - Nelsonville
796, Hofstra University
797, Hollins University
798, Holy Family University
799, Holyoke Community College
800, Homeland Security Studies And Analysis Institute
801, Homeland Security Systems Engineering And Development Institute
802, Hood College
803, Hope College
804, Horry-Georgetown Technical College
805, Hospital For Special Surgery
806, Houston Baptist University
807, Houston Community College
808, Howard Community College
809, Howard University
810, Hudson Valley Community College
811, Hudson-Alpha Institute For Biotechnology
812, Hugo W. Moser Research Institute Kennedy Krieger
813, Humboldt State University
814, Huntington Medical Research Institutes
815, Husson University
816, Huston-Tillotson University
817, Hutchinson Community College And Area Vocational School
818, Icahn School Of Medicine At Mount Sinai
819, Idaho National Laboratory
820, Idaho State University
821, IIT Research Institute
822, Ilisagvik College
823, Illinois College Of Optometry
824, Illinois Institute Of Technology
825, Illinois State University
826, Illinois Valley Community College
827, Illinois Wesleyan University
828, Immaculata University
829, Imperial Valley College
830, Indian Hills Community College
831, Indian River State College
832, Indiana Institute Of Technology
833, Indiana State University
834, Indiana University
835, Indiana University Of Pennsylvania
836, Indiana University - Bloomington
837, Indiana University - Gary
838, Indiana University - New Albany
839, Indiana University - Richmond
840, Indiana University - South Bend
841, Indiana University - Purdue University - Fort Wayne
842, Indiana University -Purdue University - Indianapolis- IUPUI
843, Indiana Wesleyan University
844, Infectious Disease Research Institute
845, Inland Northwest Research Alliance
846, Innovation Research And Training
847, Institute For Clinical Social Work - Chicago
848, Institute For Community Research
849, Institute For Molecular Medicine
850, Institute For Systems Biology
851, Institute Of American Indian Arts
852, Institute Of Transpersonal Psychology
853, Inter American University Of Puerto Rico
854, Inver Hills Community College
855, Iona College
856, Iowa Central Community College

				857, Iowa Lakes Community College<br>858, Iowa State University<br>859, Iowa Valley Community College<br>860, Iowa Western Community College<br>861, Irvine Valley College<br>862, Itawamba Community College<br>863, Ithaca College<br>864, ITT Technical Institute - Austin TX<br>865, ITT Technical Institute - Evansville IN<br>866, Ivy Tech Community College<br>867, J. Craig Venter Institute<br>868, J. David Gladstone Institutes<br>869, J.F. Drake State Technical College<br>870, Jackson State Community College<br>871, Jackson State University<br>872, Jacksonville State University<br>873, Jacksonville University<br>874, Jaeb Center For Health Research<br>875, James Madison University<br>876, Jamestown Community College<br>877, Jarvis Christian College<br>878, Jefferson College Of Health Sciences<br>879, Jefferson Davis Community College<br>880, Jefferson State Community College<br>881, Jet Propulsion Laboratory<br>882, Jewish Theological Seminary Of America<br>883, John A. Logan College<br>884, John B. Pierce Laboratory<br>885, John Bastyr College Of Naturopathic Medicine<br>886, John Brown University<br>887, John Carroll University<br>888, John F. Kennedy University- Pleasant Hill<br>889, John Wayne Cancer Institute<br>890, Johns Hopkins University<br>891, Johnson C. Smith University<br>892, Johnson County Community College<br>893, Johnson State College<br>894, Joliet Junior College<br>895, Jones County Junior College<br>896, Joslin Diabetes Center<br>897, Judiciary Engineering And Modernization Center<br>898, Judson University- Elgin<br>899, Juilliard School<br>900, Juniata College<br>901, Kalamazoo College<br>902, Kankakee Community College<br>903, Kansas City Kansas Community College<br>904, Kansas City University Of Medicine And Biosciences<br>905, Kansas State University<br>906, Kaplan College - Las Vegas<br>907, Kaskaskia College<br>908, Kean University<br>909, Keck Graduate Institute<br>910, Keene State College<br>911, Kennebec Valley Community College<br>912, Kennesaw State University<br>913, Kent State University<br>914, Kentucky Community And Technical College<br>915, Kentucky State University<br>916, Kenyon College<br>917, Kettering University<br>918, Keuka College<br>919, Keweenaw Bay Ojibwa Community College<br>920, Keystone College<br>921, King College<br>922, King's College - Wilkes Barre<br>923, Kirkwood Community College<br>924, Kishwaukee College<br>925, Knox College<br>926, Kutztown University Of Pennsylvania<br>927, La BioMedical Research Institute / Harbor UCLA Medical Center<br>928, La Jolla Bioengineering Institute<br>929, La Jolla Infectious Disease Institute<br>930, La Jolla Institute For Allergy & Immunolgy<br>931, La Roche College<br>932, La Salle University<br>933, La Sierra University<br>934, Lac Courte Oreilles Ojibwa Community College<br>935, Lafayette College<br>936, Lake City Community College<br>937, Lake Erie College Of Osteopathic Medicine											

938, Lake Forest College
939, Lake Land College
940, Lake Michigan College
941, Lake Superior State University
942, Lakeshore Technical College
943, Lamar State College - Orange
944, Lamar University
945, Lancaster General College Of Nursing And Health Sciences
946, Lander University
947, Landmark College
948, Lane College
949, Lane Community College
950, Langston University
951, Lankenau Institute For Medical Research
952, Lansing Community College
953, Laramie County Community College
954, Laredo Community College
955, Lasell College
956, Laureate Institute For Brain Research
957, Lawrence Berkeley National Laboratory
958, Lawrence Livermore National Laboratory
959, Lawrence Technological University
960, Lawrence University
961, Lawson State Community College
962, Le Moyne College
963, Lebanon Valley College
964, Lee College
965, Lee University
966, Leech Lake Tribal College
967, Lehigh Carbon Community College
968, Lehigh University
969, Leidos Biomedical Research
970, Lemoyne-Owen College
971, Lenoir Community College
972, Lesley University
973, Letourneau University
974, Lewis & Clark College
975, Lewis And Clark Community College
976, Lewis University
977, Lewis-Clark State College
978, Liberty University
979, Lieber Institute
980, Lincoln Laboratory
981, Lincoln Memorial University
982, Lincoln University Of The Commonwealth Of Pennsylvania
983, Lincoln University - Jefferson City
984, Linfield College
985, Linn Benton Community College
986, Lipscomb University
987, Little Big Horn College
988, Little Priest Tribal College
989, Livingstone College
990, Lock Haven University - Lock Haven
991, Logan College Of Chiropractic
992, Loma Linda University
993, Loma Linda Veterans Association Research & Education
994, Long Beach City College
995, Long Island University - Brooklyn
996, Long Island University - Brookville
997, Longwood University
998, Lorain County Community College
999, Loras College
1000, Los Alamos National Lab - National Security Science
1001, Los Alamos National Laboratory
1002, Los Angeles City College
1003, Los Angeles College Of Chiropractic
1004, Los Angeles Community College
1005, Los Angeles County College Of Nursing And Allied Health
1006, Los Angeles Southwest College
1007, Los Angeles Valley College
1008, Los Rios Community College
1009, Louis V. Gerstner Jr. Graduate School Of Biomedical Sciences At Memorial
1010, Louisburg College
1011, Louisiana State University Health Science Center - Shreveport
1012, Louisiana State University
1013, Louisiana State University Medical Center Shreveport
1014, Louisiana State University - Baton Rouge
1015, Louisiana State University - New Orleans - Health Sciences Center
1016, Louisiana State University - Shreveport
1017, Louisiana Tech University

1018, Lourdes College
1019, Lovelace Biomedical & Environmental Res
1020, Loyola Marymount University
1021, Loyola University Chicago
1022, Loyola University Maryland
1023, Loyola University New Orleans
1024, Lsu Health Sciences Center
1025, Lsu Pennington Biomedical Research Center
1026, Ludwig Institute For Cancer Research Ltd
1027, Lurleen B. Wallace Community College
1028, Luther College
1029, Luther Seminary
1030, Lutheran School Of Theology At Chicago
1031, Lutheran Theological Seminary At Philadelphia
1032, Lycoming College
1033, Lynchburg College
1034, Lyndon State College
1035, Lynn University
1036, Lyon College
1037, Macalester College
1038, Macomb Community College
1039, Madison Area Technical College
1040, Madonna University
1041, Magee-Women's Research Institute And Foundation
1042, Maharishi University Of Management
1043, Maine Maritime Academy
1044, Maine Medical Center
1045, Malone University
1046, Manchester Community College
1047, Manhattan Area Technical College
1048, Manhattan College
1049, Manhattan School Of Music
1050, Mansfield University Of Pennsylvania
1051, Marian University - Fond Du Lac
1052, Marian University - Indianapolis
1053, Maricopa Community College
1054, Marietta College
1055, Marine Biological Laboratory
1056, Marine Corps University
1057, Marion Military Institute
1058, Marist College
1059, Marlboro College
1060, Marquette University
1061, Marshall University
1062, Martin University
1063, Mary Baldwin College
1064, Mary Holmes College
1065, Marygrove College
1066, Maryland Institute College Of Art
1067, Marymount University
1068, Maryville College
1069, Maryville University Of St. Louis
1070, Marywood University
1071, Massachusetts Bay Community College
1072, Massachusetts Board Of Higher Education
1073, Massachusetts College Of Liberal Arts
1074, Massachusetts College Of Pharmacy And Health Sciences
1075, Massachusetts Eye And Ear Infirmary
1076, Massachusetts General Hospital
1077, Massachusetts Institute Of Technology
1078, Massachusetts Maritime Academy
1079, Massachusetts State College
1080, Massasoit Community College
1081, Maui Community College
1082, Max Planck Florida Corporation
1083, Mayland Community College
1084, Mayo Clinic Arizona
1085, Mayo Clinic Jacksonville
1086, Mayo Clinic Rochester
1087, Mayo Graduate School
1088, Mayville State University
1089, Mccormick Theological Seminary
1090, Mcdaniel College
1091, Mckendree University
1092, Mclean Hospital
1093, Mcneese State University
1094, Medaille College
1095, Medical College Of Wisconsin
1096, Medical University Of South Carolina
1097, Medstar Health Research Institute
1098, Meharry Medical College
1099, Merced Community

College
1100, Mercer University
1101, Mercy College Of Ohio
1102, Mercy College
1103, Mercyhurst College
1104, Meredith College
1105, Merrimack College
1106, Merritt College
1107, Mesa State College
1108, Messiah College
1109, Methodist Hospital Research Institute
1110, Methodist University
1111, Metropolitan College Of New York
1112, Metropolitan Community College - Fort Omaha Campus
1113, Metropolitan Community College - Kansas City
1114, Metropolitan Community College - Penn Valley
1115, Metropolitan State College Of Denver
1116, Metropolitan State University
1117, Mgh Institute Of Health Professions
1118, Miami Dade College
1119, Miami University
1120, Michigan Public Health Institute
1121, Michigan State University
1122, Michigan Technological University
1123, Mid Michigan Community College
1124, Mid-America Baptist Theological Seminary
1125, Middle Tennessee School Of Anesthesia
1126, Middle Tennessee State University
1127, Middlebury College
1128, Middlesex Community College
1129, Middlesex County College
1130, Midland College
1131, Midlands Technical College
1132, Mid-South Community College
1133, Midwest Research Institute
1134, Midwestern Baptist Theological Seminary
1135, Midwestern State University
1136, Midwestern University
1137, Miles College
1138, Millersville University Of Pennsylvania
1139, Millikin University
1140, Mills College
1141, Millsaps College
1142, Milwaukee Area Technical College
1143, Milwaukee Institute Of Art & Design
1144, Milwaukee School Of Engineering
1145, Mineral Area College
1146, Minneapolis Community And Technical College
1147, Minneapolis Medical Research Fdn
1148, Minnesota State Colleges And Universities
1149, Minnesota State Community And Technical College
1150, Minnesota State University - Mankato
1151, Minnesota State University - Moorhead
1152, Minot State University
1153, Miriam Hospital
1154, Misericordia University
1155, Mississippi College
1156, Mississippi Delta Community College
1157, Mississippi Gulf Coast Community College
1158, Mississippi State University
1159, Mississippi University For Women
1160, Mississippi Valley State University
1161, Missouri Southern State University
1162, Missouri State University
1163, Missouri University Of Science And Technology
1164, Missouri Western State University
1165, Moberly Area Community College
1166, Mohave Community College
1167, Mohawk Valley Community College
1168, Molloy College
1169, Monell Chemical Senses Center
1170, Monmouth College
1171, Monmouth University
1172, Monroe Community College
1173, Montana State University
1174, Montana State University - Billings
1175, Montana State University - Bozeman

1176, Montana State University - Havre
1177, Montana Tech Of University Of Montana
1178, Montana University
1179, Montclair State University
1180, Montefiore Medical Center
1181, Monterey College Of Law
1182, Monterey Institute Of International Studies
1183, Monterey Peninsula College
1184, Montgomery College
1185, Montgomery Community College
1186, Montgomery County Community College
1187, Moore College Of Art And Design
1188, Moorpark College
1189, Moraine Valley Community College
1190, Moravian College
1191, Morehead State University
1192, Morehouse College
1193, Morehouse School Of Medicine
1194, Morgan State University
1195, Morgridge Institute For Research
1196, Morris Brown College
1197, Morris College
1198, Mott Community College
1199, Mount Aloysius College
1200, Mount Carmel College Of Nursing
1201, Mount Desert Island Biological Lab
1202, Mount Holyoke College
1203, Mount Hood Community College
1204, Mount Ida College
1205, Mount Mercy University
1206, Mount Sacred Heart College
1207, Mount Saint Mary College - Newburgh
1208, Mount San Jacinto College
1209, Mount Sinai School Of Medicine
1210, Mount St. Mary's College
1211, Mount St. Mary's University
1212, Mountain State University
1213, Mt. San Antonio College
1214, Mt. Wachusett Community College
1215, Muhlenberg College
1216, Murray State College
1217, Murray State University
1218, Muskegon Community College
1219, Muskingum University
1220, Naes College Chicago
1221, Naropa University
1222, Nash Community College
1223, Nashville State Community College
1224, Nassau Community College
1225, Nathan S. Kline Institute For Psych Res
1226, National Biodefense Analysis And Countermeasures Center
1227, National Bureau Of Economic Research
1228, National Center For Atmospheric Research
1229, The National Center on Addiction and Substance Abuse
1230, National College (Bayomon PR)
1231, National College Of Natural Medicine
1232, National Cybersecurity Center Of Excellence
1233, National Defense Research Institute
1234, National Defense University
1235, National Development & Research Institutes
1236, National Disease Research Interchange
1237, National Flight Test Institute
1238, National Jewish Health
1239, National Optical Astronomy Observatory
1240, National Partnership for Environmental Technology Education
1241, National Radio Astronomy Observatory
1242, National Renewable Energy Laboratory
1243, National Security Engineering Center
1244, National Solar Observatory
1245, National Technological University
1246, National University
1247, National University Of Health Sciences
1248, National-Louis University
1249, Navajo Technical College
1250, Naval Postgraduate School
1251, Naval War College
1252, Nazareth College
1253, Nebraska Indian

Community College
1254, Nebraska Methodist College
1255, Nebraska Wesleyan University
1256, Nemours Children's Clinic
1257, Neumann University
1258, Neuropsychiatric Research Institute
1259, New College Of Florida
1260, New England College Of Optometry
1261, New England Conservatory
1262, New England Research Institutes
1263, New England School Of Acupuncture
1264, New Jersey City University
1265, New Jersey Institute Of Technology
1266, New Jersey Institute Of Technology
1267, New Mexico Highlands University
1268, New Mexico Institute Of Mining And Technology
1269, New Mexico Junior College
1270, New Mexico Military Institute
1271, New Mexico State University
1272, New Orleans Baptist Theological Seminary
1273, The New School
1274, New York Blood Center
1275, New York Chiropractic College
1276, New York College Of Podiatric Medicine
1277, New York Genome Center
1278, New York Institute Of Technology
1279, New York Institute Of Technology
1280, New York Law School
1281, New York Medical College
1282, New York Structural Biology Center
1283, New York University
1284, New York University School Of Medicine
1285, Newberry College
1286, Newman University
1287, NHTI - Concord's Community College
1288, Niagara County Community College
1289, Niagara University
1290, Nicholls State University
1291, Norfolk State University
1292, Normandale Community College
1293, North American Association of Central Cancer Registries
1294, North Carolina Agricultural and Technical State University
1295, North Carolina Agricultural And Technical State University
1296, North Carolina Central University
1297, North Carolina Community College
1298, North Carolina State University
1299, North Central College
1300, North Central Institute
1301, North Dakota State College Of Science
1302, North Dakota State University
1303, North Florida Community College
1304, North Georgia College & State University
1305, North Harris Montgomery Community College
1306, North Hennepin Community College
1307, North Iowa Area Community College
1308, North Orange County Community College
1309, North Park University
1310, North Shore Community College
1311, Northampton Community College
1312, Northeast Community College
1313, Northeast Iowa Community College
1314, Northeast Louisiana Technical College
1315, Northeast Ohio Medical University
1316, Northeast State Technical Community College
1317, Northeast Texas Community College
1318, Northeast Wisconsin Technical College
1319, Northeastern Illinois University
1320, Northeastern Oklahoma Agricultural & Mechanical College
1321, Northeastern State University
1322, Northeastern University
1323, Northern Arizona

				University<br>1324, Northern California Institute for Research and Education<br>1325, Northern Essex Community College<br>1326, Northern Illinois University<br>1327, Northern Kentucky University<br>1328, Northern Marianas College<br>1329, Northern Michigan University<br>1330, Northern New Mexico College<br>1331, Northern State University<br>1332, Northern Wyoming Community College<br>1333, Northland College<br>1334, Northshore Technical Community College - Greensburg<br>1335, Northshore University Health System<br>1336, Northside Hospital Atlanta<br>1337, Northwest Indian College<br>1338, Northwest Missouri State University<br>1339, Northwest Nazarene University<br>1340, Northwestern College - Orange City<br>1341, Northwestern Health Sciences University<br>1342, Northwestern Michigan College<br>1343, Northwestern State University Of Louisiana<br>1344, Northwestern University<br>1345, Norwich University<br>1346, Notre Dame De Namur University<br>1347, Notre Dame Of Maryland University<br>1348, Nova Southeastern University<br>1349, Novelmed Therapeutics<br>1350, NYSDOH - New York State Department of Health and Health Research<br>1351, Oak Crest Institute Of Science<br>1352, Oak Ridge National Laboratory<br>1353, Oakland University<br>1354, Oakton Community College<br>1355, Oakwood University<br>1356, Oberlin College<br>1357, Occidental College<br>1358, Ocean County College<br>1359, Ocean State Research Institute<br>1360, Oglala Lakota College<br>1361, Ohio Christian University<br>1362, Ohio College Of Podiatric Medicine<br>1363, Ohio Dominican University<br>1364, Ohio Northern University<br>1365, Ohio State University<br>1366, Ohio University<br>1367, Ohio Valley University<br>1368, Ohio Wesleyan University<br>1369, Ohlone College<br>1370, Oklahoma City Community College<br>1371, Oklahoma City University<br>1372, Oklahoma Medical Research Foundation<br>1373, Oklahoma Panhandle State University<br>1374, Oklahoma State University<br>1375, Old Dominion University<br>1376, Olivet Nazarene University<br>1377, Olivet University<br>1378, Oral Roberts University<br>1379, Orange Coast College<br>1380, Orangeburg-Calhoun Technical College<br>1381, Oregon Center For Applied Science<br>1382, Oregon College Of Oriental Medicine<br>1383, Oregon Graduate Institute Of Science And Engineering<br>1384, Oregon Health & Science University<br>1385, Oregon Institute Of Technology<br>1386, Oregon Research Institute<br>1387, Oregon State University<br>1388, Oregon University<br>1389, Otero Junior College<br>1390, Otterbein University<br>1391, Ouachita Baptist University<br>1392, Ouachita Technical College<br>1393, Our Lady Of Holy Cross College<br>1394, Our Lady Of The Lake College<br>1395, Our Lady Of The Lake University<br>1396, Pace University<br>1397, Pacific Institute For Research And Evaluation<br>1398, Pacific Lutheran University<br>1399, Pacific Northwest National Laboratory<br>1400, Pacific Northwest Research Institute										

1401, Pacific Northwest University Of Health Sciences
1402, Pacific States University
1403, Pacific Union College
1404, Pacific University
1405, Paine College
1406, Palau Community College
1407, Palm Beach Community College
1408, Palmer College Of Chiropractic - Davenport
1409, Palmer College Of Chiropractic - Florida Campus
1410, Palo Alto University
1411, Palo Alto Veterans Instit For Research
1412, Palomar College
1413, Pamlico Community College
1414, Park Nicollet Institute
1415, Park University
1416, Parkland College
1417, Pasadena City College
1418, Passaic County Community College
1419, Paul Smith's College
1420, Pearl River Community College
1421, Peirce College
1422, Pellissippi State Community College
1423, Peninsula College
1424, Pennsylvania College Of Technology
1425, Pennsylvania Institute Of Technology
1426, Pennsylvania State University
1427, Pennsylvania State University - Altoona
1428, Pennsylvania State University - Beaver
1429, Pennsylvania State University - Berks
1430, Pennsylvania State University - Dunmore
1431, Pennsylvania State University - Erie
1432, Pennsylvania State University - Harrisburg
1433, Pennsylvania State University - Malvern - Great Valley School Of Graduate Professional Studies
1434, Pennsylvania State University - Mckeesport
1435, Pennsylvania State University - University Park And Hershey Medical Center
1436, Pepperdine University
1437, Peralta Community College
1438, Peru State College
1439, Pfeiffer University
1440, Philadelphia College Of Osteopathic Medicine
1441, Philadelphia University
1442, Philander Smith College
1443, Phillips Community College Of The University Of Arkansas
1444, Phoenix College
1445, Piedmont Community College
1446, Piedmont Technical College
1447, Pikes Peak Community College
1448, Pikeville College
1449, Pima Community College
1450, Pine Manor College
1451, Pine Technical College
1452, Pitt Community College
1453, Pittsburg State University
1454, Pitzer College
1455, Plymouth State University
1456, Point Loma Nazarene University
1457, Polk State College
1458, Polytechnic Institute Of New York University
1459, Polytechnic University Of Puerto Rico
1460, Pomona College
1461, Ponce School Of Medicine
1462, Pontifical Catholic University Of Puerto Rico
1463, Portland Community College
1464, Portland State University
1465, Prairie View A&M University
1466, Pratt Institute
1467, Presbyterian College
1468, Prescott College
1469, Presentation College
1470, Prince George's Community College
1471, Princeton Plasma Physics Laboratory
1472, Princeton Theological Seminary
1473, Princeton University
1474, Project Air Force
1475, Proteogenomics Research Institute for Systems Medicine
1476, Providence College
1477, Providence Portland Medical Center
1478, Public Health Institute
1479, Public Health Solutions
1480, Puget Sound Blood Center

1481, Pulaski Technical College
1482, Purdue University
1483, Purdue University - Calumet Campus
1484, Purdue University - North Central
1485, Purdue University - West Lafayette
1486, Queens University Of Charlotte
1487, Quinnipiac University
1488, Quinsigamond Community College
1489, Radford University
1490, Ramapo College Of New Jersey
1491, Rancho Santiago Community College
1492, Rand Corporation
1493, Randolph-Macon College
1494, Raritan Valley Community College
1495, Reading Area Community College
1496, Red Rocks Community College
1497, Reed College
1498, Regenerative Medical Solutions
1499, Regent University
1500, Regis College
1501, Regis University
1502, Rehabilitation Institute Of Chicago
1503, Reid State Technical College
1504, Rend Lake College
1505, Rensselaer Polytechnic Institute
1506, Rensselaer Polytechnic Institute - Troy
1507, Rensselaer Polytechnic University - Hartford
1508, Renton Technical College
1509, Research Institute Nationwide Children's Hosp
1510, Research Institute Of Fox Chase Cancer Center
1511, Rhode Island College
1512, Rhode Island Hospital
1513, Rhode Island School Of Design
1514, Rhodes College
1515, Rice University
1516, Rider University - Lawrenceville
1517, Ridgewater College
1518, Rio Hondo College
1519, Ripon College
1520, River Valley Community College
1521, Riverside Community College - Riverside
1522, Riverside Research Institute
1523, Rivier College
1524, Roane State Community College - Harrisman
1525, Roanoke College
1526, Robert Morris University
1527, Roberts Wesleyan College
1528, Robeson Community College
1529, Rochester College
1530, Rochester General Hospital
1531, Rochester Institute Of Technology
1532, Rock Valley College
1533, Rockefeller University
1534, Rockhurst University
1535, Rockland Community College
1536, Rocky Mountain College
1537, Roger Williams Medical Center
1538, Roger Williams University
1539, Roger Williams University
1540, Rogers State University
1541, Rogue Community College
1542, Rollins College
1543, Roosevelt University
1544, Rosalind Franklin University Of Medicine And Science
1545, Rose State College
1546, Rose-Hulman Institute Of Technology
1547, Roseman University Of Health Sciences
1548, Rosemont College
1549, Roskamp Institute
1550, Roswell Park Cancer Institute Corp
1551, Rowan University
1552, Roxbury Community College
1553, Rush University
1554, Russell Sage College
1555, Rust College
1556, Rutgers Biomedical Health Sciences - Robert Wood Johnson Medical School
1557, Rutgers Biomedical Health Sciences - School Of Public Health
1558, Rutgers Biomedical Health Sciences - Cancer Institute of New Jersey
1559, Rutgers Biomedical Health Sciences - New Jersey Medical School

1560, Rutgers The State University Of New Jersey - Camden
1561, Rutgers The State University Of New Jersey - New Brunswick
1562, Rutgers The State University Of New Jersey - Newark
1563, Sacred Heart University
1564, Saddleback College
1565, Saginaw Chippewa Tribal College
1566, Saginaw Valley State University
1567, Saint Anselm College
1568, Saint Augustine's College
1569, Saint Francis University
1570, Saint John's University - Collegeville
1571, Saint Joseph College - West Hartford
1572, Saint Joseph Mercy Health System
1573, Saint Joseph's College Of Maine
1574, Saint Joseph's University
1575, Saint Leo University
1576, Saint Louis University - Saint Louis
1577, Saint Luke's Hospital
1578, Saint Martin's University
1579, Saint Mary's College Of California
1580, Saint Mary's College - Notre Dame
1581, Saint Mary's University Of Minnesota
1582, Saint Michael's College
1583, Saint Paul's College
1584, Saint Peter's College - Jersey City
1585, Saint Vincent College
1586, Saint Xavier University - Chicago
1587, Salem International University
1588, Salem State University
1589, Salisbury University
1590, Salish Kootenai College
1591, Salk Institute For Biological Studies
1592, Salt Lake Community College
1593, Salus University
1594, Salve Regina University
1595, Sam Houston State University
1596, Samford University
1597, Samuel Merritt University - Oakland
1598, San Bernardino Valley College
1599, San Diego Biomedical Research Institute
1600, San Diego City College
1601, San Diego Mesa College
1602, San Diego State University
1603, San Francisco State University
1604, San Jacinto College
1605, San Joaquin Valley College Inc.
1606, San Jose State University
1607, San Jose - Evergreen Community College
1608, San Juan Bautista School Of Medicine
1609, San Juan College
1610, San Mateo County Community College
1611, Sandia Corp - Sandia National Laboratories
1612, Sandia National Laboratories
1613, Sanford Research / USD
1614, Santa Barbara City College
1615, Santa Clara University
1616, Santa Fe Community College - Gainesville
1617, Santa Fe Community College - Santa Fe
1618, Santa Monica College
1619, Santa Rosa Junior College
1620, Sarah Lawrence College
1621, Savannah River National Laboratory
1622, Savannah State University
1623, Savannah Technical College
1624, Saybrook University
1625, Schepens Eye Research Institute
1626, School Of Professional Psychology At Forest Institute
1627, The School Of The Art Institute Of Chicago
1628, Schoolcraft College
1629, Schreiner University
1630, Science And Technology Policy Institute
1631, Scintillon Institute For Photobiology
1632, Scripps College
1633, Scripps Health
1634, Scripps Research Institute - California
1635, Scripps Research Institute - Florida
1636, Seattle Biomedical

Research Institute
1637, Seattle Central Community College
1638, Seattle Children's Hospital
1639, Seattle Institute For Biomedical and Clinical Research
1640, Seattle Pacific University
1641, Seattle University
1642, Seminole State College
1643, Seton Hall University
1644, Seton Hill University
1645, Sewanee The University Of The South
1646, Shasta College
1647, Shaw University
1648, Shawnee Community College
1649, Shawnee State University
1650, Shelby State Community College
1651, Sheldon Jackson College
1652, Shelton State Community College
1653, Shenandoah University
1654, Shepherd University
1655, Shippensburg University Of Pennsylvania
1656, Shoreline Community College
1657, Siena College
1658, Siena Heights University
1659, Sierra College
1660, Sierra Nevada College
1661, Simmons College
1662, Sinclair Community College
1663, Sinte Gleska University
1664, Sisseton Wahpeton College
1665, Sisters Of Charity Medical Center School Of Nursing
1666, Sitting Bull College
1667, Skagit Valley College
1668, Skidaway Institute Of Oceanography
1669, Skidmore College
1670, Slac National Accelerator Laboratory
1671, Slippery Rock University Of Pennsylvania
1672, Sloan-Kettering Institute Cancer Research
1673, Smith College
1674, Smith-Kettlewell Eye Research Institute
1675, Smithsonian Institution
1676, Snead State Community College
1677, Snow College
1678, Software Engineering Institute
1679, Sojourner-Douglass College
1680, Sonoma State University
1681, South Carolina State University
1682, South Central College
1683, South Dakota School Of Mines & Technology
1684, South Dakota State University
1685, South Florida Community College
1686, South Mountain Community College
1687, South Orange County Community College
1688, South Plains College
1689, South Puget Sound Community College
1690, South Suburban College Of Cook County
1691, South Texas College
1692, South Texas College Of Law
1693, Southeast Community College Area
1694, Southeast Missouri State University
1695, Southeastern Baptist Theological Seminary
1696, Southeastern Louisiana University
1697, Southeastern Oklahoma State University
1698, Southeastern University
1699, Southern Arkansas University
1700, Southern Baptist Theological Seminary
1701, Southern California College Of Optometry
1702, Southern California Institute For Research and Education
1703, Southern College Of Optometry
1704, Southern Connecticut State University
1705, Southern Illinois University Edwardsville
1706, Southern Illinois University Sch Of Med
1707, Southern Illinois University
1708, Southern Illinois University - Carbondale
1709, Southern Methodist University
1710, Southern Nazarene University
1711, Southern Nevada Cancer

				Research Fdn<br>1712, Southern New Hampshire University<br>1713, Southern Oregon University<br>1714, Southern Polytechnic State University<br>1715, Southern Research Institute<br>1716, Southern State Community College<br>1717, Southern University Agricultural Research And Extension Center<br>1718, Southern University And A&M College<br>1719, Southern University And A&M College - Baton Rouge<br>1720, Southern University And A&M College - New Orleans<br>1721, Southern Utah University<br>1722, Southern Vermont College<br>1723, Southwest Florida College<br>1724, Southwest Research Institute<br>1725, Southwest Texas Junior College<br>1726, Southwest Wisconsin Technical College<br>1727, Southwestern Baptist Theological Seminary<br>1728, Southwestern College - Chula Vista<br>1729, Southwestern Community College - Creston LA<br>1730, Southwestern Indian Polytechnic Institute<br>1731, Southwestern Michigan College<br>1732, Southwestern Oklahoma State University<br>1733, Southwestern Oregon Community College<br>1734, Southwestern University<br>1735, Spalding University<br>1736, Spartanburg Technical College<br>1737, Spaulding Rehabilitation Hospital<br>1738, Spectrum Health Hospitals<br>1739, Spelman College<br>1740, Spring Hill College<br>1741, Springfield College<br>1742, Springfield Technical Community College<br>1743, Sra International<br>1744, St. Alphonsus College<br>1745, St. Ambrose University<br>1746, St. Augustine Community College<br>1747, St. Barnabas Medical Center<br>1748, St. Bonaventure University<br>1749, St. Catharine College<br>1750, St. Catherine University<br>1751, St. Charles County Community College<br>1752, St. Cloud State University<br>1753, St. Edward's University<br>1754, St. Elizabeth College Of Nursing<br>1755, St. Francis College<br>1756, St. John Fisher College<br>1757, St. John's College<br>1758, St. John's University - New York City<br>1759, St. Joseph's College<br>1760, St. Joseph's Hospital And Medical Center<br>1761, St. Lawrence University<br>1762, St. Louis Community College<br>1763, St. Louis University<br>1764, St. Luke's - Roosevelt Institute For Health Sciences<br>1765, St. Mary's College Of Maryland<br>1766, St. Mary's University - San Antonio<br>1767, St. Norbert College<br>1768, St. Olaf College<br>1769, St. Petersburg College<br>1770, St. Thomas University - Miami Gardens<br>1771, St. Vincent Catholic Medical Centers Of New York<br>1772, St. Vladimir's Orthodox Theological Seminary<br>1773, Stanford University<br>1774, Stark State College<br>1775, State Center Community College<br>1776, State College Of Optometry<br>1777, State Fair Community College<br>1778, State University Of New York At Buffalo<br>1779, State University Of New York At New Paltz<br>1780, State University Of New York At Stony Brook - Stony Brook<br>1781, State University Of New York College At Brockport<br>1782, State University Of New York College At Buffalo<br>1783, State University Of New York College At Cortland<br>1784, State University Of New									

York College At Fredonia
1785, State University Of New York College At Geneseo
1786, State University Of New York College At Old Westbury
1787, State University Of New York College At Oneonta
1788, State University Of New York College At Oswego
1789, State University Of New York College At Plattsburgh
1790, State University Of New York College At Potsdam
1791, State University Of New York College Of Agriculture And Technology At Cobleskill
1792, State University Of New York College Of Agriculture And Technology At Morrisville
1793, State University Of New York College Of Environmental Science And Forestry
1794, State University Of New York College Of Optometry
1795, State University Of New York College Of Technology - Alfred
1796, State University Of New York Empire State College
1797, State University Of New York Farmingdale State College
1798, State University Of New York Fashion Institute Of Technology
1799, State University Of New York Health Science Center At Brooklyn
1800, State University Of New York Maritime College
1801, State University Of New York Purchase College
1802, State University Of New York University
1803, State University Of New York University At Albany
1804, State University Of New York Upstate Medical University
1805, State University Of New York - Binghamton University
1806, State University Of New York - Polytechnic Institute
1807, Stephen F. Austin State University
1808, Stephens College
1809, Sterling College (Sterling KS)
1810, Stetson University
1811, Stevens Institute Of Technology
1812, Stevenson University
1813, Stillman College
1814, Stone Child College
1815, Stonehill College
1816, Stowers Institute For Medical Research
1817, Strayer University
1818, Suffolk County Community College Ammerman Campus
1819, Suffolk University
1820, Sul Ross State University
1821, Sullivan Alliance to Transform the Health Professions
1822, Suny Downstate Medical Center
1823, Susquehanna University
1824, Swarthmore College
1825, Swedish Medical Center - First Hill
1826, Sweet Briar College
1827, Syracuse University
1828, Systems And Analyses Center
1829, Tacoma Community College
1830, Tacoma General Hospital
1831, Talladega College
1832, Tallahassee Community College
1833, Tarleton State University
1834, Tarleton University - Central Texas
1835, Tarrant County College
1836, Taylor University
1837, Teachers College - Columbia University
1838, Technical College Of The Lowcountry
1839, Temple College
1840, Temple University
1841, Tennessee State University
1842, Tennessee Technological University
1843, Texas A&M Agrilife Research
1844, Texas A&M Health Science Center
1845, Texas A&M Health Science Center - Baylor College Of Dentistry
1846, Texas A&M International University
1847, Texas A&M University Health Science Center
1848, Texas A&M University
1849, Texas A&M University - College Station
1850, Texas A&M University - Galveston
1851, Texas A&M University -

				Commerce<br>1852, Texas A&M University - Corpus Christi<br>1853, Texas A&M University - Kingsville<br>1854, Texas A&M University - Texarkana<br>1855, Texas Biomedical Research Institute<br>1856, Texas Christian University<br>1857, Texas College<br>1858, Texas Engineering Experiment Station<br>1859, Texas Heart Institute<br>1860, Texas Medical Center Library<br>1861, Texas Southern University<br>1862, Texas State Technical College<br>1863, Texas State University Unallocated<br>1864, Texas State University - San Marcos<br>1865, Texas Tech University Health Sciences Center - El Paso<br>1866, Texas Tech University Health Sciences Center - Lubbock<br>1867, Texas Tech University<br>1868, Texas Wesleyan University<br>1869, Texas Woman's University<br>1870, Thaddeus Stevens College Of Technology<br>1871, The Frederick S. Pardee Rand Graduate School<br>1872, The Mind Research Network<br>1873, The Richard Stockton College Of New Jersey<br>1874, Thiel College<br>1875, Thomas Edison State College<br>1876, Thomas Jefferson National Accelerator Facility<br>1877, Thomas Jefferson University<br>1878, Thomas More College<br>1879, Three Rivers Community College<br>1880, Thunderbird School Of Global Management<br>1881, Tohono O'Odham Community College<br>1882, Tompkins Cortland Community College<br>1883, Torrey Pines Institute For Molecular Studies<br>1884, Tougaloo College<br>1885, Touro College<br>1886, Touro University - Vallejo<br>1887, Towson University<br>1888, Toyota Technological Institute At Chicago<br>1889, Translational Genomics Research Inst<br>1890, Transylvania University<br>1891, Treasure Valley Community College<br>1892, Treatment Research Institute<br>1893, Tri-College University<br>1894, Tri-County Community College<br>1895, Tri-County Technical College<br>1896, Trident Technical College<br>1897, Trine University<br>1898, Trinidad State Junior College<br>1899, Trinity College - Hartford<br>1900, Trinity University<br>1901, Trinity Washington University<br>1902, Troy University<br>1903, Truckee Meadows Community College<br>1904, Truman State University<br>1905, Tufts Medical Center<br>1906, Tufts University<br>1907, Tufts University Medford<br>1908, Tulane University<br>1909, Tulsa Community College<br>1910, Turtle Mountain Community College<br>1911, Tusculum College<br>1912, Tuskegee University<br>1913, Tyler Junior College<br>1914, U.S. Walter Reed Army Institute Of Research<br>1915, Umpqua Community College<br>1916, Uniformed Services University Of The Health Sciences<br>1917, Union College - Lincoln<br>1918, Union County College<br>1919, Union Graduate College<br>1920, Union Institute & University<br>1921, Union Presbyterian Seminary - Richmond<br>1922, Union Theological Seminary<br>1923, Union University - Jackson<br>1924, United States Air Force Academy<br>1925, United States Coast Guard Academy<br>1926, United States Merchant Marine Academy<br>1927, United States Military											

Academy
1928, United States Naval Academy
1929, United States University
1930, United Tribes Technical College
1931, Unity College
1932, Universidad Adventista De Las Antillas
1933, Universidad Central Del Caribe
1934, Universidad Del Este
1935, Universidad Del Turabo
1936, Universidad Metropolitana
1937, Universities And State Colleges Of Arizona
1938, University College Of San Juan
1939, University Corporation For Atmospheric Research
1940, University Of Akron
1941, University Of Alabama At Birmingham
1942, University Of Alabama In Huntsville
1943, University Of Alabama - Tuscaloosa
1944, University Of Alabama
1945, University Of Alaska Anchorage
1946, University Of Alaska Fairbanks
1947, University Of Alaska Southeast
1948, University Of Alaska
1949, University Of Arizona
1950, University Of Arkansas At Little Rock
1951, University Of Arkansas At Monticello
1952, University Of Arkansas For Medical Sciences
1953, University Of Arkansas Pine Bluff
1954, University Of Arkansas
1955, University Of Arkansas - Fayetteville
1956, University Of Arkansas - Ft. Smith
1957, University Of Baltimore
1958, University Of Bridgeport
1959, University Of California Hastings College Of Law
1960, University Of California - Berkeley
1961, University Of California - Cooperative Extension
1962, University Of California - Davis
1963, University Of California - Irvine
1964, University Of California - Los Angeles
1965, University Of California - Merced
1966, University Of California - Riverside
1967, University Of California - San Diego
1968, University Of California - San Francisco
1969, University Of California - Santa Barbara
1970, University Of California - Santa Cruz
1971, University Of Central Arkansas
1972, University Of Central Florida
1973, University Of Central Missouri
1974, University Of Central Oklahoma
1975, University Of Charleston
1976, University Of Chicago
1977, University Of Cincinnati
1978, University Of Colorado
1979, University Of Colorado Boulder
1980, University Of Colorado Colorado Springs
1981, University Of Colorado Denver And Anschutz Medical Campus
1982, University Of Connecticut
1983, University Of Dallas
1984, University Of Dayton
1985, University Of Delaware
1986, University Of Denver
1987, University Of Detroit Mercy
1988, University Of Dubuque
1989, University Of Evansville
1990, University Of Findlay
1991, University Of Florida
1992, University Of Georgia
1993, University Of Great Falls
1994, University Of Guam
1995, University Of Hartford
1996, University Of Hawaii At Hilo
1997, University Of Hawaii At Manoa
1998, University Of Hawaii At West Oahu
1999, University Of Hawaii
2000, University Of Houston
2001, University Of Houston - Clear Lake
2002, University Of Houston - Downtown
2003, University Of Houston -

Victoria
2004, University Of Idaho
2005, University Of Illinois At Chicago
2006, University Of Illinois At Springfield
2007, University Of Illinois At Urbana-Champaign
2008, University Of Illinois
2009, University Of Indianapolis
2010, University Of Iowa
2011, University Of Kansas
2012, University Of Kentucky
2013, University Of La Verne
2014, University Of Louisiana At Lafayette
2015, University Of Louisiana At Monroe
2016, University Of Louisiana
2017, University Of Louisville
2018, University Of Maine
2019, University Of Maine At Augusta
2020, University Of Maine At Machias
2021, University Of Maine At Presque Isle
2022, University Of Mary
2023, University Of Mary Washington
2024, University Of Maryland Baltimore
2025, University Of Maryland Biotechnology Institute
2026, University Of Maryland Center For Environmental Science
2027, University Of Maryland Eastern Shore
2028, University Of Maryland
2029, University Of Maryland University College
2030, University Of Maryland - Baltimore County
2031, University Of Maryland - College Park
2032, University Of Massachusetts Amherst
2033, University Of Massachusetts Boston
2034, University Of Massachusetts Dartmouth
2035, University Of Massachusetts Lowell
2036, University Of Massachusetts Medical School
2037, University Of Massachusetts
2038, University Of Memphis
2039, University Of Miami
2040, University Of Miami School Of Medicine
2041, University Of Michigan
2042, University Of Michigan - Ann Arbor
2043, University Of Michigan - Dearborn
2044, University Of Michigan - Flint
2045, University Of Minnesota
2046, University Of Minnesota - Crookston
2047, University Of Minnesota - Duluth
2048, University Of Minnesota - Minneapolis
2049, University Of Minnesota - Morris
2050, University Of Minnesota - Rochester
2051, University Of Mississippi Medical Center
2052, University Of Mississippi
2053, University Of Mississippi - Jackson - Medical Center
2054, University Of Missouri
2055, University Of Missouri - Columbia
2056, University Of Missouri - Kansas City
2057, University Of Missouri - Saint Louis
2058, University Of Montana Western
2059, University Of Montana - Missoula College
2060, University Of Montana
2061, University Of Montevallo
2062, University Of Nebraska At Kearney
2063, University Of Nebraska At Omaha
2064, University Of Nebraska Medical Center
2065, University Of Nebraska
2066, University Of Nebraska - Lincoln
2067, University Of Nevada - Las Vegas
2068, University Of Nevada - Reno
2069, University Of New England
2070, University Of New Hampshire
2071, University Of New Haven
2072, University Of New Mexico Health Sciences Center
2073, University Of New Mexico
2074, University Of New Orleans
2075, University Of North Alabama
2076, University Of North

Carolina At Asheville
2077, University Of North Carolina At Chapel Hill
2078, University Of North Carolina At Charlotte
2079, University Of North Carolina At Greensboro
2080, University Of North Carolina At Pembroke
2081, University Of North Carolina At Wilmington
2082, University Of North Carolina
2083, University Of North Dakota
2084, University Of North Florida
2085, University Of North Texas - Denton
2086, University Of North Texas - Health Science Center
2087, University Of Northern Colorado
2088, University Of Northern Iowa
2089, University Of Notre Dame
2090, University Of Oklahoma Health Sciences Center
2091, University Of Oklahoma
2092, University Of Oregon
2093, University Of Pennsylvania
2094, University Of Phoenix
2095, University Of Pittsburgh
2096, University Of Pittsburgh - Bradford
2097, University Of Pittsburgh - Pittsburgh
2098, University Of Portland
2099, University Of Puerto Rico
2100, University Of Puerto Rico At Aguadilla
2101, University Of Puerto Rico At Arecibo
2102, University Of Puerto Rico At Bayamon
2103, University Of Puerto Rico At Carolina
2104, University Of Puerto Rico At Cayey
2105, University Of Puerto Rico At Humacao
2106, University Of Puerto Rico At Mayaguez
2107, University Of Puerto Rico At Ponce
2108, University Of Puerto Rico At Rio Piedras
2109, University Of Puerto Rico La Montana Regional Colleges
2110, University Of Puerto Rico Rio Piedras
2111, University Of Puerto Rico - Medical Sciences Campus
2112, University Of Puget Sound
2113, University Of Redlands
2114, University Of Rhode Island
2115, University Of Richmond
2116, University Of Rio Grande
2117, University Of Rochester
2118, University Of Sacred Heart
2119, University Of Saint Francis
2120, University Of Saint Mary - Leavenworth
2121, University Of San Diego
2122, University Of San Francisco
2123, University Of Scranton
2124, University Of Sioux Falls
2125, University Of South Alabama
2126, University Of South Carolina
2127, University Of South Carolina - Aiken
2128, University Of South Carolina - Beaufort
2129, University Of South Carolina - Columbia
2130, University Of South Carolina - Spartanburg
2131, University Of South Dakota
2132, University Of South Florida Polytechnic
2133, University Of South Florida Sarasota-Manatee
2134, University Of South Florida St. Petersburg
2135, University Of South Florida - Tampa
2136, University Of Southern California
2137, University Of Southern Indiana
2138, University Of Southern Maine
2139, University Of Southern Mississippi
2140, University Of St. Francis
2141, University Of St. Thomas - Minnesota - Saint Paul
2142, University Of St. Thomas - Houston
2143, University Of Tampa
2144, University Of Tennessee Health Sci Center
2145, University Of Tennessee
2146, University Of Tennessee -

				Agricultural Institute<br>2147, University Of Tennessee - Chattanooga<br>2148, University Of Tennessee - Health Science Center<br>2149, University Of Tennessee - Knoxville<br>2150, University Of Tennessee - Martin<br>2151, University Of Tennessee - Tullahoma - Space Institute<br>2152, University Of Texas At Arlington<br>2153, University Of Texas At Austin<br>2154, University Of Texas At Brownsville<br>2155, University Of Texas At Dallas<br>2156, University Of Texas At El Paso<br>2157, University Of Texas At Permian Basin<br>2158, University Of Texas At San Antonio<br>2159, University Of Texas At Tyler<br>2160, University Of Texas Health Science Center At Houston<br>2161, University Of Texas Health Science Center At San Antonio<br>2162, University Of Texas M.D. Anderson Cancer Center<br>2163, University Of Texas Medical Branch<br>2164, University Of Texas Southwestern Medical Center<br>2165, University Of Texas - Pan American<br>2166, University Of The Cumberlands<br>2167, University Of The District Of Columbia<br>2168, University Of The Incarnate Word<br>2169, University Of The Pacific<br>2170, University Of The Sciences Philadelphia<br>2171, University Of The Virgin Islands<br>2172, University Of The West<br>2173, University Of Toledo<br>2174, University Of Toledo - Health Science Campus<br>2175, University Of Tulsa<br>2176, University Of Utah<br>2177, University Of Vermont<br>2178, University Of Virginia<br>2179, University Of Virginia College At Wise<br>2180, University Of Washington - Bothell<br>2181, University Of Washington - Seattle<br>2182, University Of Washington - Tacoma<br>2183, University Of West Alabama<br>2184, University Of West Florida<br>2185, University Of West Georgia<br>2186, University Of Western States<br>2187, University Of Wisconsin Colleges<br>2188, University Of Wisconsin<br>2189, University Of Wisconsin - Eau Claire<br>2190, University Of Wisconsin - Green Bay<br>2191, University Of Wisconsin - La Crosse<br>2192, University Of Wisconsin - Madison<br>2193, University Of Wisconsin - Milwaukee<br>2194, University Of Wisconsin - Oshkosh<br>2195, University Of Wisconsin - Parkside<br>2196, University Of Wisconsin - Platteville<br>2197, University Of Wisconsin - River Falls<br>2198, University Of Wisconsin - Stevens Point<br>2199, University Of Wisconsin - Stout<br>2200, University Of Wisconsin - Superior<br>2201, University Of Wisconsin - Whitewater<br>2202, University Of Wyoming<br>2203, Urban College Of Boston<br>2204, Ursinus College<br>2205, Ursuline College<br>2206, Utah State University<br>2207, Utah State University - Price - College Of Eastern Utah<br>2208, Utah Valley University<br>2209, Ut-Battelle - Oak Ridge National Lab<br>2210, Utica College<br>2211, Valdosta State University<br>2212, Valencia Community College<br>2213, Valley City State University<br>2214, Valparaiso University<br>2215, Van Andel Research											

Institute
2216, Vanderbilt University
2217, Vanguard University
2218, Vassar College
2219, Ventura County Community College
2220, Vermilion Community College
2221, Vermont Technical College
2222, Via Christi Regional Medical Center
2223, Villanova University
2224, Vincennes University
2225, Virginia College - Lynchburg
2226, Virginia Commonwealth University
2227, Virginia Community College
2228, Virginia Institute Of Marine Science
2229, Virginia Military Institute
2230, Virginia Polytechnic Institute And State University
2231, Virginia State University
2232, Virginia Union University
2233, Virginia Wesleyan College
2234, Viterbo University
2235, Voorhees College
2236, Wabash College
2237, Wadsworth Center
2238, Wagner College
2239, Wake Forest University
2240, Wake Forest University Health Sciences
2241, Wake Technical Community College
2242, Walden University
2243, Waldorf College
2244, Walla Walla Community College
2245, Walla Walla University
2246, Wallace Community College - Dothan
2247, Wallace State Community College - Selma
2248, Wallace State Community College -Hanceville
2249, Walsh College Of Accountancy And Business Administration
2250, Walsh University
2251, Warren Wilson College
2252, Washburn University
2253, Washington & Jefferson College
2254, Washington And Lee University
2255, Washington College
2256, Washington State Community College
2257, Washington State University
2258, Washington University In St. Louis
2259, Waubonsee Community College
2260, Waukesha County Technical College
2261, Wayland Baptist University
2262, Wayne Community College
2263, Wayne State College
2264, Wayne State University
2265, Waynesburg University
2266, Weatherford College
2267, Webb Institute
2268, Weber State University
2269, Webster University - Saint Louis
2270, Weill Medical Coll Of Cornell Univ
2271, Wellesley College
2272, Wenatchee Valley College
2273, Wentworth Institute Of Technology
2274, Wesley College
2275, Wesleyan College
2276, Wesleyan University
2277, West Chester University Of Pennsylvania
2278, West Georgia Technical College
2279, West Hills Community College
2280, West Liberty University
2281, West Los Angeles College
2282, West Shore Community College
2283, West Suburban College Of Nursing
2284, West Texas A&M University
2285, West Virginia School Of Osteopathic Medicine
2286, West Virginia State University
2287, West Virginia University Institute Of Technology
2288, West Virginia University
2289, West Virginia Wesleyan College
2290, Western Carolina University
2291, Western Connecticut State University
2292, Western Illinois University
2293, Western Iowa Tech Community College
2294, Western Kentucky University

2295, Western Michigan University
2296, Western Nebraska Community College
2297, Western New England College
2298, Western New Mexico University
2299, Western Oklahoma State College
2300, Western Oregon University
2301, Western State College Of Colorado
2302, Western Texas College
2303, Western University Of Health Sciences
2304, Western Washington University
2305, Westfield State University
2306, Westminster College - Fulton
2307, Westminster College - New Wilmington
2308, Westminster College - Salt Lake City
2309, Westminster Theological Seminary
2310, Westmont College
2311, Wharton County Junior College
2312, Whatcom Community College
2313, Wheaton College - Norton
2314, Wheaton College - Wheaton
2315, Wheeling Jesuit University
2316, Wheelock College
2317, White Earth Tribal & Community College
2318, Whitehead Institute For Biomedical Res
2319, Whitman College
2320, Whittier College
2321, Whitworth University
2322, Wichita State University
2323, Widener University
2324, Wilberforce University
2325, Wiley College
2326, Wilkes Community College
2327, Wilkes University
2328, Willamette University
2329, William Beaumont Hospital Research Institute
2330, William Carey University - Hattiesburg
2331, William Jewell College
2332, William Mitchell College Of Law
2333, William Paterson University
2334, Williams College
2335, Wilmington College Of Ohio
2336, Wilmington University
2337, Wilson College
2338, Winifred Masterson Burke Med Research Institute
2339, Winona State University
2340, Winston-Salem State University
2341, Winthrop University
2342, Wisconsin Lutheran College
2343, Wisconsin Technical College
2344, Wistar Institute
2345, Wittenberg University
2346, Wofford College
2347, Wolford College
2348, Women And Infants Hospital - Rhode Island
2349, Woods Hole Oceanographic Institution
2350, Worcester Polytechnic Institute
2351, Worcester State College
2352, Wright Institute
2353, Wright State University
2354, Wyotech
2355, Xavier University
2356, Xavier University Of Louisiana
2357, Yakima Valley Community College
2358, Yale University
2359, Yavapai College
2360, Yeshiva University
2361, York College - Nebraska
2362, York College of Pennsylvania
2363, York Technical College
2364, Yosemite Community College
2365, Youngstown State University
1, Other - not listed
2, A. T. Still University Of Health Sciences
3, Aaron Diamond Aids Research Center
4, Abilene Christian University
5, Abraham Baldwin Agricultural College
6, Adams State College
7, Adelphi University
8, Adrian College
9, Aerospace Federally Funded Research And Development Center
10, Agnes Scott College

institution		dropdown	Institution or University	11, Aib College Of Business<br>12, Aiken Technical College<br>13, Aims Community College<br>14, Air Force Institute Of Technology<br>15, Alabama A&M University<br>16, Alabama Southern Community College<br>17, Alabama State University<br>18, Alamo Community College District<br>19, Alaska Pacific University<br>20, Albany College Of Pharmacy And Health Sciences<br>21, Albany Medical College<br>22, Albany Molecular Research<br>23, Albany State University<br>24, Albert Einstein Healthcare Network<br>25, Albion College<br>26, Albright College<br>27, Alcorn State University<br>28, Alderson-Broaddus College<br>29, Alfred I. Du Pont Hosp For Children<br>30, Alfred University<br>31, Allan Hancock College<br>32, Allegany College Of Maryland<br>33, Allegheny College<br>34, Allegheny University Of The Health Sciences<br>35, Allegheny-Singer Research Institute<br>36, Allen College- Waterloo<br>37, Allen Institute<br>38, Allen University<br>39, Alliant International University<br>40, Alma College<br>41, Alvernia University<br>42, Alverno College<br>43, Amarillo College<br>44, American Academy-Child/Adolescent Psych<br>45, American Association For Cancer Research<br>46, American College Of Medical Genetics<br>47, American College Of Radiology<br>48, American College-Fayetteville<br>49, American Indian Higher Education Consortium<br>50, American International College<br>51, American Samoa Community College<br>52, American Type Culture Collection<br>53, American University Of Puerto Rico- Bayamon<br>54, American University<br>55, Ames Laboratory<br>56, Amherst College<br>57, Amridge University<br>58, Ana G. Mendez University System<br>59, Andrew College<br>60, Andrew Jackson University<br>61, Andrews University<br>62, Angelo State University<br>63, Anna Maria College<br>64, Anne Arundel Community College<br>65, Anoka-Ramsey Community College<br>66, Antaya Science And Technology<br>67, Antelope Valley College<br>68, Antioch University<br>69, Apogee Biotechnology Corporation<br>70, Appalachian State University<br>71, Arapahoe Community College<br>72, Arbor Research Collaborative For Health<br>73, Arcadia University<br>74, Argonne National Laboratory<br>75, Argosy University<br>76, Arizona State University<br>77, Arizona Western College<br>78, Arkansas Baptist College<br>79, Arkansas Children'S Hospital Res Inst<br>80, Arkansas State University<br>81, Arkansas Tech University<br>82, Armstrong Atlantic State University<br>83, Arrowhead Community Colleges<br>84, Arroyo Center<br>85, Art Center College Of Design<br>86, Art Institute Of Seattle<br>87, Asbury Theological Seminary<br>88, Asheville-Buncombe Technical Community College<br>89, Ashland University<br>90, Assumption College<br>91, Atenas College<br>92, Atlanta University Center<br>93, Atlantic Cape Community College<br>94, Auburn University- Auburn<br>95, Auburn University-Montgomery<br>96, Augsburg College<br>97, Augusta State University<br>98, Augustana College- Rock	If your institution or university is not listed, please select "Other" and you will be able to write in your choice.	autocomplete							

Island
99, Augustana College- Sioux Falls
100, Aurora University
101, Austin College
102, Austin Community College District- Northridge Campus
103, Austin Peay State University
104, Avila University
105, Azusa Pacific University
106, Babson College
107, Baker College- Flint
108, Baker University
109, Baldwin-Wallace College
110, Ball State University
111, Baltimore City Community College
112, Bank Street College Of Education
113, Banner Alzheimer'S Institute
114, Banner Health
115, Baptist Memorial College Of Health Sciences
116, Bard College
117, Bard College At Simon'S Rock
118, Barnard College
119, Barnes-Jewish College Goldfarb School Of Nursing
120, Barry University
121, Barton County Community College
122, Baruch S. Blumberg Institute
123, Bastyr University
124, Bates College
125, Baton Rouge Community College
126, Battelle Centers/Pub Hlth Res & Evaluatn
127, Battelle Pacific Northwest Laboratories
128, Bay Area Tumor Institute
129, Bay De Noc Community College
130, Bay Mills Community College
131, Baylor College Of Medicine
132, Baylor Research Institute
133, Baylor University
134, Baystate Medical Center
135, Beaufort County Community College
136, Bellarmine University
137, Bellevue College
138, Bellin College
139, Belmont University
140, Beloit College
141, Bemidji State University
142, Benaroya Research Inst At Virginia Mason
143, Benedict College
144, Benedictine College
145, Benedictine University
146, Benjamin Franklin Institute Of Technology
147, Bennett College For Women
148, Bennington College
149, Bentley University
150, Berea College
151, Bergen Community College
152, Berkeley College- New York City
153, Berry College
154, Bessemer State Technical College
155, Beth Israel Deaconess Medical Center
156, Beth Israel Medical Ctr (New York)
157, Bethany College- Bethany
158, Bethel College (Mishawaka- In)
159, Bethel College- North Newton
160, Bethel University
161, Bethune-Cookman University
162, Bevill State Community College Walker Campus
163, Biola University
164, Biomedical Research Institute
165, Birmingham Southern College
166, Bishop State Community College
167, Bismarck State College
168, Black Hawk College
169, Black Hills State University
170, Blackfeet Community College
171, Blackhawk Technical College
172, Blood Systems Research Institute
173, Bloodcenter Of Wisconsin
174, Bloomfield College
175, Bloomsburg University Of Pennsylvania
176, Bluefield State College
177, Boise State University
178, Boston Architectural Center
179, Boston College
180, Boston Medical Center
181, Boston University
182, Boston University Medical Campus
183, Bowdoin College

184, Bowie State University
185, Bowling Green State University
186, Bradley University
187, Brandeis University
188, Brazosport College
189, Brenau University
190, Brentwood Biomedical Research Institute
191, Brescia University
192, Brevard Community College
193, Bridgewater College
194, Bridgewater State College
195, Brigham And Women'S Hospital
196, Brigham Young University
197, Brigham Young University-Provo
198, Bristol Community College
199, Brite Divinity School
200, Broad Institute
201, Brookdale Community College
202, Brookhaven National Laboratory
203, Brookhaven Science Assoc-Brookhaven Lab
204, Brooklyn Law School
205, Broward College
206, Brown Mackie College
207, Brown University
208, Brunswick Community College
209, Bryant University
210, Bryn Athyn College
211, Bryn Mawr College
212, Buck Institute For Research On Aging
213, Bucknell University
214, Bucks County Community College
215, Buena Vista University
216, Bunker Hill Community College
217, Burlington County College
218, Butler County Community College (Butler PA)
219, Butler Hospital (Providence RI)
220, Butler University
221, Butte College
222, Cabrillo College
223, Cabrini College
224, Calhoun Community College
225, California Baptist University
226, California College Of Arts And Crafts
227, California Community College
228, California Institute Of Integral Studies
229, California Institute Of Technology
230, California Institute Of The Arts
231, California Lutheran University
232, California Maritime Academy
233, California Pacific Med Ctr Res Institute
234, California Poly State U San Luis Obispo
235, California Polytechnic State University- San Luis Obispo
236, California School Of Professional Psychology-Berkeley-Alameda
237, California School Of Professional Psychology- Fresno
238, California School Of Professional Psychology- Los Angeles
239, California State Polytechnic University- Pomona
240, California State University Unallocated
241, California State University-Bakersfield
242, California State University-Channel Islands
243, California State University-Chico
244, California State University-Dominguez Hills
245, California State University-East Bay
246, California State University-Fresno
247, California State University-Fullerton
248, California State University-Long Beach
249, California State University-Los Angeles
250, California State University-Monterey Bay
251, California State University-Northridge
252, California State University-Sacramento
253, California State University-San Bernardino
254, California State University-San Marcos
255, California State University-Stanislaus
256, California University Of Pennsylvania

257, Calvin College
258, Calvin Theological Seminary
259, Cambridge Health Alliance
260, Camden County College
261, Cameron University
262, Campbell University
263, Cancer Prevention Instit Of California
264, Cancer Targeted Technology- Llc
265, Canisius College
266, Cankdeska Cikana Community College
267, Cape Cod Community College
268, Cape Fear Community College
269, Capella University
270, Capital University
271, Capitol College
272, Cardinal Stritch University
273, Caribbean University
274, Carl Albert State College
275, Carl Sandburg College
276, Carleton College
277, Carlos Albizu University
278, Carlow University
279, Carnegie Mellon University
280, Carolinas Medical Center
281, Carroll College
282, Carroll University
283, Carson-Newman College
284, Carthage College
285, Case Western Reserve University
286, Casper College
287, Castleton State College
288, Catawba College
289, Catawba Valley Community College
290, Catholic University Of America
291, Cayuga Community College
292, Cedar Crest College
293, Cedars-Sinai Medical Center
294, Centenary College
295, Centenary College Of Louisiana
296, Center For Advanced Aviation System Development
297, Center For Communications And Computing
298, Center For Construction Res And Training
299, Center For Enterprise Modernization
300, Center For Expermental Software Engr Md
301, Center For Innovative Public Health Res
302, Center For Naval Analyses
303, Center For Nuclear Waste Regulatory Analyses
304, Center For Psychological Consultation
305, Center For Social Innovation
306, Central Alabama Community College
307, Central Arizona College
308, Central College
309, Central Community College
310, Central Connecticut State University
311, Central Florida Community College
312, Central Georgia Technical College
313, Central Lakes College-Brainerd
314, Central Louisiana Technical College
315, Central Maine Community College
316, Central Michigan University
317, Central New Mexico Community College
318, Central Oregon Community College
319, Central Piedmont Community College
320, Central State University
321, Central Washington University
322, Central Wyoming College
323, Centralia College
324, Centre College
325, Centro De Estudios Multidisciplinarios
326, Century College
327, Cerritos College
328, Chadron State College
329, Chaminade University Of Honolulu
330, Chancellor University
331, Chapman University
332, Charles R. Drew University Of Med & Sci
333, Charles R. Drew University Of Medicine And Science
334, Charles River Laboratories
335, Charles Stark Draper Laboratory
336, Charleston Southern University
337, Chatham University-Pittsburgh
338, Chattanooga State Community College
339, Chemeketa Community

College
340, Chesapeake College
341, Chestnut Health Systems
342, Chestnut Hill College
343, Cheyenne River Community College
344, Cheyney University Of Pennsylvania
345, Chi Institute For Research & Innovation
346, Chicago School Of Professional Psychology
347, Chicago State University
348, Chicago Theological Seminary
349, Chief Dull Knife College
350, Children'S Hosp Of Philadelphia
351, Children'S Hospital & Res Ctr At Oakland
352, Children'S Hospital Corporation
353, Children'S Hospital Of Los Angeles
354, Children'S Mercy Hosp (Kansas City MO)
355, Children'S Research Institute
356, Chippewa Valley Technical College
357, Chowan University
358, Christian Brothers University
359, Christopher Newport University
360, Cincinnati Childrens Hosp Med Ctr
361, Cincinnati State Technical And Community College
362, Citadel Military College Of South Carolina
363, City College Of San Francisco
364, City Colleges Of Chicago
365, City Of Hope
366, City Of Hope/Beckman Research Institute
367, City University Of New York Unallocated
368, City University Of New York- School of Law
369, City University Of New York- Graduate Center
370, City University Of New York- Baruch College
371, City University Of New York - Borough Of Manhattan Community College
372, City University Of New York - Bronx Community College
373, City University Of New York - Brooklyn College
374, City University Of New York - College Of Staten Island
375, City University Of New York -Graduate Center
376, City University Of New York -Hostos Community College
377, City University Of New York - Hunter College
378, City University Of New York - John Jay College Criminal Justice
379, City University Of New York -Kingsborough Community College
380, City University Of New York - Laguardia Community College
381, City University Of New York - Lehman College
382, City University Of New York - Medgar Evers College
383, City University Of New York -New York City College Of Technology
384, City University Of New York -Queens College
385, City University Of New York - Queensborough Community College
386, City University Of New York - System Office
387, City University Of New York - The City University
388, City University Of New York - York College
389, Clackamas Community College
390, Claflin University
391, Claremont Graduate University
392, Claremont Mckenna College
393, Claremont School Of Theology
394, Clarion University Of Pennsylvania
395, Clark Atlanta University
396, Clark College
397, Clark State Community College
398, Clark University
399, Clarke University
400, Clarkson College
401, Clarkson University
402, Clatsop Community College
403, Clayton State University
404, Cleary University
405, Clemson University
406, Cleveland Clinic Lerner

Com-Cwru
407, Cleveland Community College
408, Cleveland Institute Of Music
409, Cleveland State Community College
410, Cleveland State University
411, Clinton Junior College
412, Cloud County Community College
413, Cms Alliance To Modernize Healthcare
414, Coastal Bend College
415, Coastal Carolina Community College
416, Coastal Carolina University
417, Coastline Community College
418, Cochise College
419, Coe College
420, Coker College
421, Colby College
422, Colby Community College
423, Colby-Sawyer College
424, Cold Spring Harbor Laboratory
425, Colgate University
426, College Of Charleston
427, College Of Dupage
428, College Of Idaho
429, College Of Lake County
430, College Of Menominee Nation
431, College Of Micronesia-Fsm
432, College Of Mount Saint Vincent
433, College Of Mount St. Joseph
434, College Of Nanoscale Science And Engineering Of The University At Albany Suny
435, The College Of New Jersey
436, The College Of New Rochelle
437, College Of Saint Benedict
438, College Of Saint Rose
439, College Of Southern Idaho
440, College Of Southern Maryland
441, College Of Southern Nevada
442, College Of St. Elizabeth
443, College Of St. Mary
444, College Of St. Scholastica - Duluth
445, College Of The Atlantic
446, College Of The Canyons
447, College Of The Holy Cross
448, College Of The Mainland
449, College Of The Sequoias
450, College Of William And Mary
451, College Of Wooster
452, Collin County Community College District
453, Colorado Cancer Research Program
454, Colorado College
455, Colorado Mountain College
456, Colorado School Of Mines
457, Colorado State University
458, Colorado State University-Pueblo
459, Colorado State University-System Office
460, Colorado Theological Seminary
461, Columbia Basin College
462, Columbia College Chicago
463, Columbia College-Columbia MO
464, Columbia College-Columbia SC
465, Columbia Univ New York Morningside
466, Columbia University Health Sciences
467, Columbia University In The City Of New York
468, Columbia University Teachers College
469, Columbus Community Clinical Oncology Prg
470, Columbus State Community College
471, Columbus State University
472, Comanche Nation College
473, Commonwealth Medical College
474, Community College Of Allegheny County
475, Community College Of Aurora
476, Community College Of Baltimore County Catonsville
477, Community College Of Philadelphia
478, Community College Of Rhode Island
479, Community Colleges Of Spokane District 17
480, Concord University
481, Concordia College
482, Concordia Seminary
483, Concordia Theological Seminary
484, Concordia University Chicago
485, Concordia University Wisconsin
486, Connecticut Children'S

Medical Center
487, Connecticut College
488, Connecticut State Dept Of Public Health
489, Connecticut State University
490, Connors State College
491, Consortium For Ocean Leadership
492, Contra Costa College
493, Contra Costa Community College District
494, Contra Costa Community College District - San Ramon - Diablo Valley College
495, Converse College
496, The Cooper Institute
497, Cooper Union For The Advancement Of Science And Art
498, Coppin State University
499, Coriell Institute For Medical Research
500, Cornell College
501, Cornell University
502, Corning Community College
503, County College Of Morris
504, Covenant College
505, Cox College Of Nursing And Health Sciences
506, Craven Community College
507, Creighton University
508, Crowder College
509, Crown College
510, Cuyahoga Community College
511, Daemen College
512, Dakota County Technical College
513, Dakota State University
514, Dakota Wesleyan University
515, Dallas Community College District- Eastfield College
516, Dallas Community College District- Richland College
517, Dallas Theological Seminary
518, Dana-Farber Cancer Inst
519, Dartmouth College
520, Darton College
521, Data Numerica Institute
522, Davidson College
523, Davidson County Community College
524, Davis & Elkins College
525, Dayton Clinical Oncology Program
526, Daytona State College
527, De Anza College
528, Decatur Memorial Hospital
529, Defense Acquisition University
530, Del Mar College
531, Delaware State University
532, Delaware Technical Community College
533, Delaware Valley College
534, Delgado Community College
535, Delta State University
536, Denison University
537, Denmark Technical College
538, Denver Health And Hospital Authority
539, Depaul University
540, Depauw University
541, Des Moines Area Community College
542, Des Moines University
543, Desales University
544, Desert Research Institute
545, Dickinson College
546, Dickinson State University
547, Dillard University
548, Dine College
549, Dixie State College Of Utah
550, Doane College
551, Doheny Eye Institute
552, Dominican College Of Blauvelt
553, Dominican University
554, Dominican University Of California
555, Donnelly College
556, Dordt College
557, Dowling College
558, D-Q University
559, Drake University
560, Drew University
561, Drexel University
562, Drury University
563, Duke University
564, Duquesne University
565, Durham Technical Community College
566, Dyersburg State Community College
567, D'Youville College
568, Earlham College
569, East Arkansas Community College
570, East Carolina University
571, East Central University
572, East Los Angeles College
573, East Mississippi Community College
574, East Stroudsburg University Of Pennsylvania
575, East Tennessee State

University
576, Eastern Arizona College
577, Eastern Connecticut State University
578, Eastern Idaho Technical College
579, Eastern Illinois University
580, Eastern Iowa Community College District
581, Eastern Kentucky University
582, Eastern Mennonite University
583, Eastern Michigan University
584, Eastern New Mexico University
585, Eastern Oklahoma State College
586, Eastern Oregon University
587, Eastern Virginia Medical School
588, Eastern Washington University
589, East-West University
590, Eckerd College
591, Ecog-Acrin Medical Research Foundation
592, Ecpi College Of Technology
593, Edinboro University Of Pennsylvania
594, Edison State College
595, Edison State Community College
596, Edmonds Community College
597, Edward Via College Of Osteopathic Medicine
598, Edward Waters College
599, El Camino College
600, El Camino College Compton Center
601, El Paso Community College
602, Elgin Community College
603, Elizabeth City State University
604, Elizabethtown College
605, Elmhurst College
606, Elms College
607, Elon University
608, Emanuel Hospital And Health Center
609, Embry-Riddle Aeronautical University
610, Emergent Product Development Gaithersbur
611, Emerson College
612, Emma Pendleton Bradley Hospital
613, Emmanuel College
614, Emory & Henry College
615, Emory University
616, Emporia State University
617, Endicott College
618, Erie Community College North Campus
619, Erikson Institute
620, Erskine College
621, Essentia Institute Of Rural Health
622, Essex County College
623, Everett Community College
624, Evergreen State College - Olympia
625, Evergreen Valley College
626, Experimental Pathology Laboratories
627, Fairfield University
628, Fairleigh Dickinson University
629, Fairmont State University
630, Family Health International
631, Fayetteville State University
632, Fayetteville Technical Community College
633, Feinstein Institute For Medical Research
634, Felician College
635, Fermi National Accelerator Laboratory
636, Ferris State University
637, Ferrum College
638, Fielding Graduate University
639, Finger Lakes Community College
640, Finlandia University
641, Fisk University
642, Fitchburg State University
643, Flathead Valley Community College
644, Florence-Darlington Technical College
645, Florida Agricultural And Mechanical University
646, Florida Atlantic University
647, Florida Gulf Coast University
648, Florida Hospital College Of Health Sciences
649, Florida Institute Of Technology
650, Florida International University
651, Florida Keys Community College
652, Florida Memorial University
653, Florida Metropolitan University Tampa Campus
654, Florida Southern College
655, Florida State College At Jacksonville

656, Florida State University
657, Fond Du Lac Tribal And Community College
658, Foothill-De Anza Community College District
659, Fordham University
660, Forsyth Institute
661, Forsyth Technical Community College
662, Fort Belknap College
663, Fort Berthold Community College
664, Fort Hays State University
665, Fort Lewis College
666, Fort Peck Community College
667, Fort Valley State University
668, Foundation For Aids Research
669, Fox Chase Chemical Diversity Center
670, Fox Valley Technical College
671, Framingham State College
672, Francis Marion University
673, Franciscan University Of Steubenville
674, Frank Phillips College
675, Franklin & Marshall College
676, Franklin Pierce Law Center
677, Franklin Pierce University-Rindge
678, Franklin W. Olin College Of Engineering
679, Fred Hutchinson Cancer Research Center
680, Frederick National Laboratory For Cancer Research
681, Fresno City College
682, Friends Research Institute
683, Frontier School Of Midwifery And Family Nursing
684, Frostburg State University
685, Fuller Theological Seminary- Pasadena
686, Fulton-Montgomery Community College
687, Furman University
688, Future Generations Graduate School
689, Gadsden State Community College
690, Gallaudet University
691, Gannon University
692, Garrett College
693, Garrett-Evangelical Theological Seminary
694, Gaston College
695, Gateway Community And Technical College
696, Gateway Technical College
697, Geisinger Clinic
698, Gem National Consortium For Graduate Degrees For Minorities In Engineering
699, General Electric Global Research Ctr
700, Genesee Community College
701, Geophysical Institute- Uaf
702, George Fox University
703, George Mason University
704, George Washington University
705, Georgetown College
706, Georgetown University
707, Georgia College And State University
708, Georgia Health Sciences University
709, Georgia Institute Of Technology
710, Georgia Perimeter College
711, Georgia Regents University
712, Georgia Southern University
713, Georgia Southwestern State University
714, Georgia State University
715, Georgian Court University
716, Gettysburg College
717, Glen Oaks Community College
718, Glendale Community College- Glendale
719, Glenville State College
720, Globe University - Minnesota School Of Business
721, Goddard College
722, Gogebic Community College
723, Golden Gate University
724, Gonzaga University
725, Goodwin College
726, Gordon College
727, Gordon Research Conferences
728, Goshen College
729, Goucher College
730, Governors State University
731, Graceland University
732, Graduate School Usa
733, Graduate Theological Union
734, Grambling State University
735, Grand Rapids Community College
736, Grand Valley State University
737, Grand View University
738, Grayson County College
739, Green Mountain College
740, Green River Community

College
741, Greenfield Community College
742, Greenville College
743, Greenville Health System
744, Greenville Technical College
745, Grinnell College
746, Grossmont College
747, Grossmont-Cuyamaca Community College District
748, Group Health Cooperative
749, Guam Community College
750, Guilford College
751, Gulf Coast Community College
752, Gustavus Adolphus College
753, Gwynedd-Mercy College
754, H. Lee Moffitt Cancer Ctr & Res Inst
755, Hackensack University Medical Center
756, Hagerstown Community College
757, Hamilton College
758, Hamline University
759, Hampden-Sydney College
760, Hampshire College
761, Hampton University
762, Harcum College
763, Harding University- Searcy
764, Harford Community College
765, Harper College
766, Harrisburg Area Community College
767, Harrisburg University Of Science And Technology
768, Harris-Stowe State University
769, Hartford Hospital
770, Hartnell College
771, Hartwick College
772, Harvard Medical School
773, Harvard Pilgrim Health Care
774, Harvard School Of Public Health
775, Harvard University
776, Harvey Mudd College
777, Haskell Indian Nations University
778, Hauptman-Woodward Medical Research Inst
779, Haverford College
780, Hawaii Pacific University
781, Haywood Community College
782, Healthpartners Institute
783, Heartland Community College
784, Hebrew Union College- Jewish Institute Of Religion
785, Heidelberg University
786, Hektoen Institute For Medical Research
787, Helene Fuld College Of Nursing
788, Henderson State University
789, Hendrix College
790, Henry Ford Community College
791, Henry Ford Health System
792, Henry M. Jackson Fdn For The Adv Mil/Med
793, Heritage University
794, High Point University
795, Highland Community College- Freeport- Il
796, Highland Community College- Highland
797, Highline Community College
798, Hillsborough Community College
799, Hinds Community College
800, Hiram College
801, Hobart And William Smith Colleges
802, Hocking College- Nelsonville
803, Hofstra University
804, Hollins University
805, Holy Family University
806, Holyoke Community College
807, Homeland Security Studies And Analysis Institute
808, Homeland Security Systems Engineering And Development Institute
809, Hood College
810, Hope College
811, Horry-Georgetown Technical College
812, Hospital For Special Surgery
813, Houston Baptist University
814, Houston Community College
815, Howard Community College
816, Howard University
817, Hudson Valley Community College
818, Hudson-Alpha Institute For Biotechnology
819, Hugo W. Moser Res Inst Kennedy Krieger
820, Humboldt State University
821, Huntington Medical Research Institutes

822, Husson University
823, Huston-Tillotson University
824, Hutchinson Community College And Area Vocational School
825, Icahn School Of Medicine At Mount Sinai
826, Idaho National Laboratory
827, Idaho State University
828, Iit Research Institute
829, Ilisagvik College
830, Illinois College Of Optometry
831, Illinois Institute Of Technology
832, Illinois State University
833, Illinois Valley Community College
834, Illinois Wesleyan University
835, Immaculata University
836, Imperial Valley College
837, Indian Hills Community College
838, Indian River State College
839, Indiana Institute Of Technology
840, Indiana State University
841, Indiana University
842, Indiana University Of Pennsylvania
843, Indiana University- Bloomington
844, Indiana University- Gary
845, Indiana University- New Albany
846, Indiana University- Richmond
847, Indiana University- South Bend
848, Indiana University-Purdue University- Fort Wayne
849, Indiana University-Purdue University- Indianapolis- Iupui
850, Indiana Wesleyan University
851, Infectious Disease Research Institute
852, Inland Northwest Research Alliance
853, Innovation Research And Training
854, Institute For Clinical Social Work - Chicago
855, Institute For Community Research
856, Institute For Molecular Medicine
857, Institute For Systems Biology
858, Institute Of American Indian Arts
859, Institute Of Transpersonal Psychology
860, Integral Molecular
861, Integrated Laboratory Systems
862, Inter American University Of Puerto Rico
863, Inver Hills Community College
864, Iona College
865, Iowa Central Community College
866, Iowa Lakes Community College
867, Iowa State University
868, Iowa Valley Community College District
869, Iowa Western Community College
870, Irvine Valley College
871, Itawamba Community College
872, Ithaca College
873, Itt Technical Institute- Austin TX
874, Itt Technical Institute - Evansville IN
875, Ivy Tech Community College
876, J. Craig Venter Institute
877, J. David Gladstone Institutes
878, J.F. Drake State Technical College
879, Jackson State Community College
880, Jackson State University
881, Jacksonville State University
882, Jacksonville University
883, Jaeb Center For Health Research
884, James Madison University
885, Jamestown Community College
886, Jarvis Christian College
887, Jefferson College Of Health Sciences
888, Jefferson Davis Community College
889, Jefferson State Community College
890, Jet Propulsion Laboratory
891, Jewish Theological Seminary Of America
892, John A. Logan College
893, John B. Pierce Laboratory
894, John Bastyr College Of Naturopathic Medicine
895, John Brown University
896, John Carroll University

897, John F. Kennedy University- Pleasant Hill
898, John Wayne Cancer Institute
899, Johns Hopkins University
900, Johnson C. Smith University
901, Johnson County Community College
902, Johnson State College
903, Joliet Junior College
904, Jones County Junior College
905, Joslin Diabetes Center
906, Judiciary Engineering And Modernization Center
907, Judson University- Elgin
908, Juilliard School
909, Juniata College
910, Kalamazoo College
911, Kankakee Community College
912, Kansas City Kansas Community College
913, Kansas City University Of Medicine And Biosciences
914, Kansas State University
915, Kaplan College- Las Vegas
916, Kaskaskia College
917, Kean University
918, Keck Graduate Institute
919, Keene State College
920, Kennebec Valley Community College
921, Kennesaw State University
922, Kent State University
923, Kentucky Community And Technical College System
924, Kentucky State University
925, Kenyon College
926, Kettering University
927, Keuka College
928, Keweenaw Bay Ojibwa Community College
929, Keystone College
930, King College
931, King'S College- Wilkes Barre
932, Kirkwood Community College
933, Kishwaukee College
934, Knox College
935, Kutztown University Of Pennsylvania
936, La Biomed Res Inst/ Harbor Ucla Med Ctr
937, La Jolla Bioengineering Institute
938, La Jolla Infectious Disease Institute
939, La Jolla Inst For Allergy & Immunolgy
940, La Roche College
941, La Salle University
942, La Sierra University
943, Lac Courte Oreilles Ojibwa Community College
944, Lafayette College
945, Lake City Community College
946, Lake Erie College Of Osteopathic Medicine
947, Lake Forest College
948, Lake Land College
949, Lake Michigan College
950, Lake Superior State University
951, Lakeshore Technical College
952, Lamar State College- Orange
953, Lamar University
954, Lancaster General College Of Nursing And Health Sciences
955, Lander University
956, Landmark College
957, Lane College
958, Lane Community College
959, Langston University
960, Lankenau Institute For Medical Research
961, Lansing Community College
962, Laramie County Community College
963, Laredo Community College
964, Lasell College
965, Laureate Institute For Brain Research
966, Lawrence Berkeley National Laboratory
967, Lawrence Livermore National Laboratory
968, Lawrence Technological University
969, Lawrence University
970, Lawson State Community College
971, Le Moyne College
972, Lebanon Valley College
973, Lee College
974, Lee University
975, Leech Lake Tribal College
976, Lehigh Carbon Community College
977, Lehigh University
978, Leidos Biomedical Research
979, Lemoyne-Owen College
980, Lenoir Community College
981, Lesley University
982, Letourneau University
983, Lewis & Clark College

984, Lewis And Clark Community College
985, Lewis University
986, Lewis-Clark State College
987, Liberty University
988, Lieber Institute
989, Lincoln Laboratory
990, Lincoln Memorial University
991, Lincoln University Of The Commonwealth Of Pennsylvania
992, Lincoln University-Jefferson City
993, Linfield College
994, Linn Benton Community College
995, Lipscomb University
996, Little Big Horn College
997, Little Priest Tribal College
998, Livingstone College
999, Lock Haven University-Lock Haven
1000, Logan College Of Chiropractic
1001, Loma Linda University
1002, Loma Linda Veterans Assn Research & Educ
1003, Long Beach City College
1004, Long Island University-Brooklyn
1005, Long Island University-Brookville
1006, Longwood University
1007, Lorain County Community College
1008, Loras College
1009, Los Alamos Nat Secty-Los Alamos Nat Lab
1010, Los Alamos National Laboratory
1011, Los Angeles City College
1012, Los Angeles College Of Chiropractic
1013, Los Angeles Community College District
1014, Los Angeles County College Of Nursing And Allied Health
1015, Los Angeles Southwest College
1016, Los Angeles Valley College
1017, Los Rios Community College District
1018, Louis V. Gerstner Jr. Graduate School Of Biomedical Sciences At Memorial
1019, Louisburg College
1020, Louisiana State Univ Hsc Shreveport
1021, Louisiana State University
1022, Louisiana State University Medical Center Shreveport
1023, Louisiana State University- Baton Rouge
1024, Louisiana State University- New Orleans- Health Sciences Center
1025, Louisiana State University- Shreveport
1026, Louisiana Tech University
1027, Louisiana Universities Marine Consortium
1028, Lourdes College
1029, Lovelace Biomedical & Environmental Res
1030, Loyola Marymount University
1031, Loyola University Chicago
1032, Loyola University Maryland
1033, Loyola University New Orleans
1034, Lsu Health Sciences Center
1035, Lsu Pennington Biomedical Research Ctr
1036, Ludwig Institute For Cancer Res Ltd
1037, Lurleen B. Wallace Community College
1038, Luther College
1039, Luther Seminary
1040, Lutheran School Of Theology At Chicago
1041, Lutheran Theological Seminary At Philadelphia
1042, Lycoming College
1043, Lynchburg College
1044, Lyndon State College
1045, Lynn University
1046, Lyon College
1047, Macalester College
1048, Macomb Community College
1049, Madison Area Technical College
1050, Madonna University
1051, Magee-Women'S Res Inst And Foundation
1052, Maharishi University Of Management
1053, Maine Maritime Academy
1054, Maine Medical Center
1055, Malone University
1056, Manchester Community College
1057, Manhattan Area Technical College
1058, Manhattan College
1059, Manhattan School Of Music
1060, Mansfield University Of

Pennsylvania
1061, Marian University- Fond Du Lac
1062, Marian University- Indianapolis
1063, Maricopa Community College
1064, Marietta College
1065, Marine Biological Laboratory
1066, Marine Corps University
1067, Marion Military Institute
1068, Marist College
1069, Marlboro College
1070, Marquette University
1071, Marshall University
1072, Martin University
1073, Mary Baldwin College
1074, Mary Holmes College
1075, Marygrove College
1076, Maryland Institute College Of Art
1077, Marymount University
1078, Maryville College
1079, Maryville University Of St. Louis
1080, Marywood University
1081, Massachusetts Bay Community College
1082, Massachusetts Board Of Higher Education
1083, Massachusetts College Of Liberal Arts
1084, Massachusetts College Of Pharmacy And Health Sciences
1085, Massachusetts Eye And Ear Infirmary
1086, Massachusetts General Hospital
1087, Massachusetts Institute Of Technology
1088, Massachusetts Maritime Academy
1089, Massachusetts State College- System Office
1090, Massasoit Community College
1091, Maui Community College
1092, Max Planck Florida Corporation
1093, Mayland Community College
1094, Mayo Clinic Arizona
1095, Mayo Clinic Jacksonville
1096, Mayo Clinic Rochester
1097, Mayo Graduate School
1098, Mayville State University
1099, Mccormick Theological Seminary
1100, Mcdaniel College
1101, Mckendree University
1102, Mclean Hospital
1103, Mcneese State University
1104, Medaille College
1105, Medical College Of Wisconsin
1106, Medical University Of South Carolina
1107, Medstar Health Research Institute
1108, Meharry Medical College
1109, Merced Community College District
1110, Mercer University
1111, Mercy College Of Ohio
1112, Mercy College
1113, Mercyhurst College
1114, Meredith College
1115, Merrimack College
1116, Merritt College
1117, Mesa State College
1118, Messiah College
1119, Methodist Hospital Research Institute
1120, Methodist University
1121, Metropolitan College Of New York
1122, Metropolitan Community College- Fort Omaha Campus
1123, Metropolitan Community College- Kansas City
1124, Metropolitan Community College- Penn Valley
1125, Metropolitan State College Of Denver
1126, Metropolitan State University
1127, Mgh Institute Of Health Professions
1128, Miami Dade College
1129, Miami University
1130, Michigan Public Health Institute
1131, Michigan State University
1132, Michigan Technological University
1133, Mid Michigan Community College
1134, Mid-America Baptist Theological Seminary
1135, Middle Tennessee School Of Anesthesia
1136, Middle Tennessee State University
1137, Middlebury College
1138, Middlesex Community College
1139, Middlesex County College
1140, Midland College
1141, Midlands Technical College
1142, Mid-South Community College

1143, Midwest Research Institute
1144, Midwestern Baptist Theological Seminary
1145, Midwestern State University
1146, Midwestern University
1147, Miles College
1148, Millersville University Of Pennsylvania
1149, Millikin University
1150, Mills College
1151, Millsaps College
1152, Milwaukee Area Technical College
1153, Milwaukee Institute Of Art & Design
1154, Milwaukee School Of Engineering
1155, Mineral Area College
1156, Minneapolis Community And Technical College
1157, Minneapolis Medical Research Fdn
1158, Minnesota State Colleges And Universities
1159, Minnesota State Community And Technical College
1160, Minnesota State University- Mankato
1161, Minnesota State University- Moorhead
1162, Minot State University
1163, Miriam Hospital
1164, Misericordia University
1165, Mississippi College
1166, Mississippi Delta Community College
1167, Mississippi Gulf Coast Community College
1168, Mississippi Research Consortium
1169, Mississippi State University
1170, Mississippi University For Women
1171, Mississippi Valley State University
1172, Missouri Southern State University
1173, Missouri State University
1174, Missouri University Of Science And Technology
1175, Missouri Western State University
1176, Moberly Area Community College
1177, Mohave Community College
1178, Mohawk Valley Community College
1179, Molloy College
1180, Monell Chemical Senses Center
1181, Monmouth College
1182, Monmouth University
1183, Monroe Community College
1184, Montana State University
1185, Montana State University- Billings
1186, Montana State University- Bozeman
1187, Montana State University- Havre
1188, Montana Tech Of University Of Montana
1189, Montana University System- System Office
1190, Montclair State University
1191, Montefiore Medical Center
1192, Monterey College Of Law
1193, Monterey Institute Of International Studies
1194, Monterey Peninsula College
1195, Montgomery College
1196, Montgomery Community College
1197, Montgomery County Community College
1198, Moore College Of Art And Design
1199, Moorpark College
1200, Moraine Valley Community College
1201, Moravian College
1202, Morehead State University
1203, Morehouse College
1204, Morehouse School Of Medicine
1205, Morgan State University
1206, Morgridge Institute For Research
1207, Morris Brown College
1208, Morris College
1209, Mott Community College
1210, Mount Aloysius College
1211, Mount Carmel College Of Nursing
1212, Mount Desert Island Biological Lab
1213, Mount Holyoke College
1214, Mount Hood Community College
1215, Mount Ida College
1216, Mount Mercy University
1217, Mount Sacred Heart College
1218, Mount Saint Mary College- Newburgh

1219, Mount San Jacinto College
1220, Mount Sinai School Of Medicine
1221, Mount St. Mary'S College
1222, Mount St. Mary'S University
1223, Mountain State University
1224, Mt. San Antonio College
1225, Mt. Wachusett Community College
1226, Muhlenberg College
1227, Murray State College
1228, Murray State University
1229, Muskegon Community College
1230, Muskingum University
1231, Naes College Chicago
1232, Naropa University
1233, Nash Community College
1234, Nashville State Community College
1235, Nassau Community College
1236, Nathan S. Kline Institute For Psych Res
1237, National Biodefense Analysis And Countermeasures Center
1238, National Bureau Of Economic Research
1239, National Center For Atmospheric Research
1240, National Center On Addiction/Sub Abuse
1241, National College (Bayomon PR)
1242, National College Of Natural Medicine
1243, National Cybersecurity Center Of Excellence
1244, National Defense Research Institute
1245, National Defense University
1246, National Development & Res Institutes
1247, National Disease Research Interchange
1248, National Flight Test Institute
1249, National Jewish Health
1250, National Optical Astronomy Observatory
1251, National Partnership/Environmntl/Tech/Ed
1252, National Physical Science Consortium
1253, National Radio Astronomy Observatory
1254, National Renewable Energy Laboratory
1255, National Security Engineering Center
1256, National Solar Observatory
1257, National Technological University
1258, National University
1259, National University Of Health Sciences
1260, National-Louis University
1261, Navajo Technical College
1262, Naval Postgraduate School
1263, Naval War College
1264, Nazareth College
1265, Nebraska Indian Community College
1266, Nebraska Methodist College
1267, Nebraska Wesleyan University
1268, Nemours Children'S Clinic
1269, Neumann University
1270, Neuropsychiatric Research Institute
1271, Nevada System Of Higher Education
1272, New College Of Florida
1273, New England College Of Optometry
1274, New England Conservatory
1275, New England Research Institutes
1276, New England School Of Acupuncture
1277, New Jersey City University
1278, New Jersey Institute Of Technology
1279, New Jersey Institute Of Technology
1280, New Mexico Highlands University
1281, New Mexico Institute Of Mining And Technology
1282, New Mexico Junior College
1283, New Mexico Military Institute
1284, New Mexico State University
1285, New Orleans Baptist Theological Seminary
1286, The New School
1287, New York Blood Center
1288, New York Chiropractic College
1289, New York College Of Podiatric Medicine
1290, New York Genome Center
1291, New York Inst Of Technology
1292, New York Institute Of

Technology
1293, New York Law School
1294, New York Medical College
1295, New York Structural Biology Center
1296, New York University
1297, New York University School Of Medicine
1298, Newberry College
1299, Newman University
1300, Concord'S Community College
1301, Niagara County Community College
1302, Niagara University
1303, Nicholls State University
1304, Nonagen Bioscience Corporation
1305, Norfolk State University
1306, Normandale Community College
1307, North American Assn/Central Cancer Reg
1308, North Carolina Agri & Tech St Univ
1309, North Carolina Agricultural And Technical State University
1310, North Carolina Central University
1311, North Carolina Community College System
1312, North Carolina State University
1313, North Central College
1314, North Central Institute
1315, North Dakota State College Of Science
1316, North Dakota State University
1317, North Florida Community College
1318, North Georgia College & State University
1319, North Harris Montgomery Community College District
1320, North Hennepin Community College
1321, North Iowa Area Community College
1322, North Orange County Community College District
1323, North Park University
1324, North Shore Community College
1325, Northampton Community College
1326, Northeast Community College
1327, Northeast Iowa Community College
1328, Northeast Louisiana Technical College
1329, Northeast Ohio Medical University
1330, Northeast State Technical Community College
1331, Northeast Texas Community College
1332, Northeast Wisconsin Technical College
1333, Northeastern Illinois University
1334, Northeastern Oklahoma Agricultural & Mechanical College
1335, Northeastern State University
1336, Northeastern University
1337, Northern Arizona University
1338, Northern California Institute/Res/Edu
1339, Northern Essex Community College
1340, Northern Illinois University
1341, Northern Kentucky University
1342, Northern Marianas College
1343, Northern Michigan University
1344, Northern New Mexico College
1345, Northern State University
1346, Northern Wyoming Community College District
1347, Northland College
1348, Northshore Technical Community College- Greensburg
1349, Northshore University Healthsystem
1350, Northside Hospital Atlanta
1351, Northwest Indian College
1352, Northwest Missouri State University
1353, Northwest Nazarene University
1354, Northwestern College- Orange City
1355, Northwestern Health Sciences University
1356, Northwestern Michigan College
1357, Northwestern State University Of Louisiana
1358, Northwestern University
1359, Norwich University
1360, Notre Dame De Namur University
1361, Notre Dame Of Maryland University
1362, Nova Southeastern University

1363, Novelmed Therapeutics
1364, Nysdoh/Health Researchs
1365, Oak Crest Institute Of Science
1366, Oak Ridge National Laboratory
1367, Oakland University
1368, Oakton Community College
1369, Oakwood University
1370, Oberlin College
1371, Occidental College
1372, Ocean County College
1373, Ocean State Research Institute
1374, Oglala Lakota College
1375, Ohio Christian University
1376, Ohio College Of Podiatric Medicine
1377, Ohio Dominican University
1378, Ohio Northern University
1379, Ohio State University
1380, Ohio University
1381, Ohio Valley University
1382, Ohio Wesleyan University
1383, Ohlone College
1384, Oklahoma City Community College
1385, Oklahoma City University
1386, Oklahoma Medical Research Foundation
1387, Oklahoma Panhandle State University
1388, Oklahoma State University
1389, Old Dominion University
1390, Olivet Nazarene University
1391, Olivet University
1392, Oral Roberts University
1393, Orange Coast College
1394, Orangeburg-Calhoun Technical College
1395, Oregon Center For Applied Science
1396, Oregon College Of Oriental Medicine
1397, Oregon Graduate Institute Of Science And Engineering
1398, Oregon Health & Science University
1399, Oregon Institute Of Technology
1400, Oregon Research Institute
1401, Oregon State University
1402, Oregon University System
1403, Organization
1404, Otero Junior College
1405, Otterbein University
1406, Ouachita Baptist University
1407, Ouachita Technical College
1408, Our Lady Of Holy Cross College
1409, Our Lady Of The Lake College
1410, Our Lady Of The Lake University
1411, Pace University
1412, Pacific Institute For Res And Evaluation
1413, Pacific Lutheran University
1414, Pacific Northwest National Laboratory
1415, Pacific Northwest Research Institute
1416, Pacific Northwest University Of Health Sciences
1417, Pacific States University
1418, Pacific Union College
1419, Pacific University
1420, Paine College
1421, Palau Community College
1422, Palm Beach Community College
1423, Palmer College Of Chiropractic- Davenport
1424, Palmer College Of Chiropractic- Florida Campus
1425, Palo Alto University
1426, Palo Alto Veterans Instit For Research
1427, Palomar College
1428, Pamlico Community College
1429, Park Nicollet Institute
1430, Park University
1431, Parkland College
1432, Pasadena City College
1433, Passaic County Community College
1434, Paul Smith'S College
1435, Pearl River Community College
1436, Peirce College
1437, Pellissippi State Community College
1438, Peninsula College
1439, Pennsylvania College Of Technology
1440, Pennsylvania Institute Of Technology
1441, Pennsylvania State System Higher Education- Office Of Chancellor
1442, Pennsylvania State University
1443, Pennsylvania State University - Altoona
1444, Pennsylvania State University - Beaver
1445, Pennsylvania State

University - Berks
1446, Pennsylvania State University - Dunmore
1447, Pennsylvania State University - Erie
1448, Pennsylvania State University - Harrisburg
1449, Pennsylvania State University - Malvern - Great Valley School Of Graduate Professional Studies
1450, Pennsylvania State University - Mckeesport
1451, Pennsylvania State University - University Park And Hershey Medical Center
1452, Pepperdine University
1453, Peralta Community College
1454, Peru State College
1455, Pfeiffer University
1456, Philadelphia College Of Osteopathic Medicine
1457, Philadelphia University
1458, Philander Smith College
1459, Phillips Community College Of The University Of Arkansas
1460, Phoenix College
1461, Piedmont Community College
1462, Piedmont Technical College
1463, Pikes Peak Community College
1464, Pikeville College
1465, Pima Community College
1466, Pine Manor College
1467, Pine Technical College
1468, Pitt Community College
1469, Pittsburg State University
1470, Pitzer College
1471, Plymouth State University
1472, Point Loma Nazarene University
1473, Polk State College
1474, Polytechnic Institute Of New York University
1475, Polytechnic University Of Puerto Rico
1476, Pomona College
1477, Ponce School Of Medicine
1478, Pontifical Catholic University Of Puerto Rico
1479, Population Council
1480, Portland Community College
1481, Portland State University
1482, Prairie View A&M University
1483, Pratt Institute
1484, Presbyterian College
1485, Prescott College
1486, Presentation College
1487, Prince George'S Community College
1488, Princeton Plasma Physics Laboratory
1489, Princeton Theological Seminary
1490, Princeton University
1491, Project Air Force
1492, Proteogenomics Research Instit/Sys/ Med
1493, Providence College
1494, Providence Portland Medical Center
1495, Public Health Institute
1496, Public Health Solutions
1497, Puget Sound Blood Center
1498, Pulaski Technical College
1499, Purdue University
1500, Purdue University- Calumet Campus
1501, Purdue University- North Central
1502, Purdue University- West Lafayette
1503, Quantum Research Corporation
1504, Queens University Of Charlotte
1505, Quinnipiac University
1506, Quinsigamond Community College
1507, Radford University
1508, Ramapo College Of New Jersey
1509, Rancho Santiago Community College District
1510, Rand Corporation
1511, Randolph-Macon College
1512, Raritan Valley Community College
1513, Reading Area Community College
1514, Red Rocks Community College
1515, Reed College
1516, Regenerative Medical Solutions
1517, Regent University
1518, Regis College
1519, Regis University
1520, Rehabilitation Institute Of Chicago
1521, Reid State Technical College
1522, Rend Lake College
1523, Rensselaer Polytechnic Institute
1524, Rensselaer Polytechnic

Institute- Troy
1525, Rensselaer Polytechnic University- Hartford
1526, Renton Technical College
1527, Research Inst Nationwide Children'S Hosp
1528, Research Inst Of Fox Chase Can Ctr
1529, Rhode Island College
1530, Rhode Island Hospital
1531, Rhode Island School Of Design
1532, Rhodes College
1533, Rice University
1534, Rider University- Lawrenceville
1535, Ridgewater College
1536, Rio Hondo College
1537, Ripon College
1538, River Valley Community College
1539, Riverside Community College District- Riverside
1540, Riverside Research Institute
1541, Rivier College
1542, Roane State Community College- Harrisman
1543, Roanoke College
1544, Robert Morris University
1545, Roberts Wesleyan College
1546, Robeson Community College
1547, Rochester College
1548, Rochester General Hospital
1549, Rochester Institute Of Technology
1550, Rock Valley College
1551, Rockefeller University
1552, Rockhurst University
1553, Rockland Community College
1554, Rocky Mountain College
1555, Roger Williams Medical Center
1556, Roger Williams University System Office
1557, Roger Williams University
1558, Rogers State University
1559, Rogue Community College
1560, Rollins College
1561, Roosevelt University
1562, Rosalind Franklin University Of Medicine And Science
1563, Rose State College
1564, Rose-Hulman Institute Of Technology
1565, Roseman University Of Health Sciences
1566, Rosemont College
1567, Roskamp Institute
1568, Roswell Park Cancer Institute Corp
1569, Rowan University
1570, Roxbury Community College
1571, Rush University
1572, Russell Sage College
1573, Rust College
1574, Rutgers Biomedical Health Sciences - Robert Wood Johnson Medical School
1575, Rutgers Biomedical Health Sciences - School Of Public Health
1576, Rutgers Biomedical Health Sciences - Cancer Institute of New Jersey
1577, Rutgers Biomedical Health Sciences - New Jersey Medical School
1578, Rutgers The State University Of New Jersey - Camden
1579, Rutgers The State University Of New Jersey - New Brunswick
1580, Rutgers The State University Of New Jersey - Newark
1581, Sacred Heart University
1582, Saddleback College
1583, Saginaw Chippewa Tribal College
1584, Saginaw Valley State University
1585, Saint Anselm College
1586, Saint Augustine'S College
1587, Saint Francis University
1588, Saint John'S University- Collegeville
1589, Saint Joseph College- West Hartford
1590, Saint Joseph Mercy Health System
1591, Saint Joseph'S College Of Maine
1592, Saint Joseph'S University
1593, Saint Leo University
1594, Saint Louis University- Saint Louis
1595, Saint Luke'S Hospital
1596, Saint Martin'S University
1597, Saint Mary'S College Of California
1598, Saint Mary'S College- Notre Dame
1599, Saint Mary'S University Of Minnesota
1600, Saint Michael'S College

1601, Saint Paul'S College
1602, Saint Peter'S College-Jersey City
1603, Saint Vincent College
1604, Saint Xavier University-Chicago
1605, Salem International University
1606, Salem State University
1607, Salisbury University
1608, Salish Kootenai College
1609, Salk Institute For Biological Studies
1610, Salt Lake Community College
1611, Salus University
1612, Salve Regina University
1613, Sam Houston State University
1614, Samford University
1615, Samuel Merritt University-Oakland
1616, San Bernardino Valley College
1617, San Diego Biomedical Research Institute
1618, San Diego City College
1619, San Diego Mesa College
1620, San Diego State University
1621, San Francisco State University
1622, San Jacinto College
1623, San Joaquin Valley College Inc.
1624, San Jose State University
1625, San Jose/Evergreen Community College District
1626, San Juan Bautista School Of Medicine
1627, San Juan College
1628, San Mateo County Community College District
1629, Sandia Corp-Sandia National Laboratories
1630, Sandia National Laboratories
1631, Sanford Research/Usd
1632, Santa Barbara City College
1633, Santa Clara University
1634, Santa Fe Community College- Gainesville
1635, Santa Fe Community College- Santa Fe
1636, Santa Monica College
1637, Santa Rosa Junior College
1638, Sarah Lawrence College
1639, Savannah River National Laboratory
1640, Savannah State University
1641, Savannah Technical College
1642, Saybrook University
1643, Schepens Eye Research Institute
1644, School Of Professional Psychology At Forest Institute
1645, The School Of The Art Institute Of Chicago
1646, Schoolcraft College
1647, Schreiner University
1648, Science And Technology Policy Institute
1649, Scintillon Institute For Photobiology
1650, Scripps College
1651, Scripps Health
1652, Scripps Research Institute - California
1653, Scripps Research Institute - Florida
1654, Seattle Biomedical Research Institute
1655, Seattle Central Community College
1656, Seattle Children'S Hospital
1657, Seattle Inst For Biomedical/Clinical Res
1658, Seattle Pacific University
1659, Seattle University
1660, Seminole State College
1661, Seton Hall University
1662, Seton Hill University
1663, Sewanee The University Of The South
1664, Shasta College
1665, Shaw University
1666, Shawnee Community College
1667, Shawnee State University
1668, Shelby State Community College
1669, Sheldon Jackson College
1670, Shelton State Community College
1671, Shenandoah University
1672, Shepherd University
1673, Shippensburg University Of Pennsylvania
1674, Shoreline Community College
1675, Siena College
1676, Siena Heights University
1677, Sierra College
1678, Sierra Nevada College
1679, Silver Lake Research Corporation
1680, Simmons College
1681, Sinclair Community College

1682, Sinte Gleska University
1683, Sisseton Wahpeton College
1684, Sisters Of Charity Medical Center School Of Nursing
1685, Sitting Bull College
1686, Skagit Valley College
1687, Skidaway Institute Of Oceanography
1688, Skidmore College
1689, Slac National Accelerator Laboratory
1690, Slippery Rock University Of Pennsylvania
1691, Sloan-Kettering Inst Can Research
1692, Smith College
1693, Smith-Kettlewell Eye Research Institute
1694, Smithsonian Institution
1695, Snead State Community College
1696, Snow College
1697, Software Engineering Institute
1698, Sojourner-Douglass College
1699, Sonoma State University
1700, South Carolina Sea Grant Consortium
1701, South Carolina State University
1702, South Central College
1703, South Dakota School Of Mines & Technology
1704, South Dakota State University
1705, South Florida Community College
1706, South Mountain Community College
1707, South Orange County Community College District
1708, South Plains College
1709, South Puget Sound Community College
1710, South Suburban College Of Cook County
1711, South Texas College
1712, South Texas College Of Law
1713, Southeast Clinical Oncol Res Consortium
1714, Southeast Community College Area
1715, Southeast Missouri State University
1716, Southeastern Baptist Theological Seminary
1717, Southeastern Louisiana University
1718, Southeastern Oklahoma State University
1719, Southeastern University
1720, Southern Arkansas University
1721, Southern Baptist Theological Seminary
1722, Southern California College Of Optometry
1723, Southern California Inst For Res/Educ
1724, Southern College Of Optometry
1725, Southern Connecticut State University
1726, Southern Illinois University Edwardsville
1727, Southern Illinois University Sch Of Med
1728, Southern Illinois University
1729, Southern Illinois University- Carbondale
1730, Southern Methodist University
1731, Southern Nazarene University
1732, Southern Nevada Cancer Research Fdn
1733, Southern New Hampshire University
1734, Southern Oregon University
1735, Southern Polytechnic State University
1736, Southern Research Institute
1737, Southern State Community College
1738, Southern University Agricultural Research And Extension Center
1739, Southern University And A&M College
1740, Southern University And A&M College- Baton Rouge
1741, Southern University And A&M College- New Orleans
1742, Southern Utah University
1743, Southern Vermont College
1744, Southwest Florida College
1745, Southwest Research Institute
1746, Southwest Texas Junior College
1747, Southwest Wisconsin Technical College
1748, Southwestern Baptist Theological Seminary
1749, Southwestern College- Chula Vista

1750, Southwestern Community College - Creston LA
1751, Southwestern Indian Polytechnic Institute
1752, Southwestern Michigan College
1753, Southwestern Oklahoma State University
1754, Southwestern Oregon Community College
1755, Southwestern University
1756, Spalding University
1757, Spartanburg Technical College
1758, Spaulding Rehabilitation Hospital
1759, Spectrum Health Hospitals
1760, Spelman College
1761, Spring Hill College
1762, Springfield College
1763, Springfield Technical Community College
1764, Sra International
1765, St. Alphonsus College
1766, St. Ambrose University
1767, St. Augustine Community College
1768, St. Barnabas Medical Center
1769, St. Bonaventure University
1770, St. Catharine College
1771, St. Catherine University
1772, St. Charles County Community College
1773, St. Cloud State University
1774, St. Edward'S University
1775, St. Elizabeth College Of Nursing
1776, St. Francis College
1777, St. John Fisher College
1778, St. John's College
1779, St. John's University- New York City
1780, St. Joseph'S College
1781, St. Joseph'S Hospital And Medical Center
1782, St. Lawrence University
1783, St. Louis Community College
1784, St. Louis University
1785, St. Luke's-Roosevelt Inst For Health Sciences
1786, St. Mary's College Of Maryland
1787, St. Mary's University- San Antonio
1788, St. Norbert College
1789, St. Olaf College
1790, St. Petersburg College
1791, St. Thomas University- Miami Gardens
1792, St. Vincent Catholic Medical Centers Of New York
1793, St. Vladimir's Orthodox Theological Seminary
1794, Stanford University
1795, Stark State College
1796, State Center Community College
1797, State College Of Optometry
1798, State Fair Community College
1799, State University Of New York At Buffalo
1800, State University Of New York At New Paltz
1801, State University Of New York At Stony Brook- Stony Brook
1802, State University Of New York College At Brockport
1803, State University Of New York College At Buffalo
1804, State University Of New York College At Cortland
1805, State University Of New York College At Fredonia
1806, State University Of New York College At Geneseo
1807, State University Of New York College At Old Westbury
1808, State University Of New York College At Oneonta
1809, State University Of New York College At Oswego
1810, State University Of New York College At Plattsburgh
1811, State University Of New York College At Potsdam
1812, State University Of New York College Of Agriculture And Technology At Cobleskill
1813, State University Of New York College Of Agriculture And Technology At Morrisville
1814, State University Of New York College Of Environmental Science And Forestry
1815, State University Of New York College Of Optometry
1816, State University Of New York College Of Technology- Alfred
1817, State University Of New York Empire State College
1818, State University Of New York Farmingdale State College
1819, State University Of New York Fashion Institute Of

				Technology<br>1820, State University Of New York Health Science Center At Brooklyn<br>1821, State University Of New York Maritime College<br>1822, State University Of New York Purchase College<br>1823, State University Of New York University<br>1824, State University Of New York University At Albany<br>1825, State University Of New York Upstate Medical University<br>1826, State University Of New York- Binghamton U.<br>1827, State University Of New York- Polytechnic Institute<br>1828, State University System Of Florida<br>1829, Stephen F. Austin State University<br>1830, Stephens College<br>1831, Sterling College (Sterling KS)<br>1832, Stetson University<br>1833, Stevens Institute Of Technology<br>1834, Stevenson University<br>1835, Stillman College<br>1836, Stone Child College<br>1837, Stonehill College<br>1838, Stowers Institute For Medical Research<br>1839, Strayer University<br>1840, Suffolk County Community College Ammerman Campus<br>1841, Suffolk University<br>1842, Sul Ross State University<br>1843, Sullivan Alliance/Transform/Hlth/Profess<br>1844, Suny Downstate Medical Center<br>1845, Susquehanna University<br>1846, Swarthmore College<br>1847, Swedish Medical Center- First Hill<br>1848, Sweet Briar College<br>1849, Syracuse University<br>1850, Systems And Analyses Center<br>1851, Tacoma Community College<br>1852, Tacoma General Hospital<br>1853, Talladega College<br>1854, Tallahassee Community College<br>1855, Tarleton State University<br>1856, Tarleton University System Center-Central Texas<br>1857, Tarrant County College District<br>1858, Taylor University<br>1859, Teachers College- Columbia University<br>1860, Technical College Of The Lowcountry<br>1861, Temple College<br>1862, Temple University<br>1863, Tennessee State University<br>1864, Tennessee Technological University<br>1865, Texas A&M Agrilife Research<br>1866, Texas A&M Health Science Center<br>1867, Texas A&M Health Science Center- Baylor College Of Dentistry<br>1868, Texas A&M International University<br>1869, Texas A&M University Health Science Ctr<br>1870, Texas A&M University<br>1871, Texas A&M University- College Station<br>1872, Texas A&M University- Galveston<br>1873, Texas A&M University- Commerce<br>1874, Texas A&M University- Corpus Christi<br>1875, Texas A&M University- Kingsville<br>1876, Texas A&M University- Texarkana<br>1877, Texas Biomedical Research Institute<br>1878, Texas Christian University<br>1879, Texas College<br>1880, Texas Engineering Experiment Station<br>1881, Texas Heart Institute<br>1882, Texas Medical Center Library<br>1883, Texas Southern University<br>1884, Texas State Technical College<br>1885, Texas State University Unallocated<br>1886, Texas State University- San Marcos<br>1887, Texas Tech University Health Sciences Center- El Paso<br>1888, Texas Tech University Health Sciences Center- Lubbock<br>1889, Texas Tech University<br>1890, Texas Wesleyan University										

1891, Texas Woman'S University
1892, Thaddeus Stevens College Of Technology
1893, The Frederick S. Pardee Rand Graduate School
1894, The Mind Research Network
1895, The Richard Stockton College Of New Jersey
1896, Thiel College
1897, Thomas Edison State College
1898, Thomas Jefferson National Accelerator Facility
1899, Thomas Jefferson University
1900, Thomas More College
1901, Three Rivers Community College
1902, Thunderbird School Of Global Management
1903, Tohono O'Odham Community College
1904, Tompkins Cortland Community College
1905, Torrey Pines Inst For Molecular Studies
1906, Tougaloo College
1907, Touro College
1908, Touro University- Vallejo
1909, Towson University
1910, Toyota Technological Institute At Chicago
1911, Translational Genomics Research Inst
1912, Transylvania University
1913, Treasure Valley Community College
1914, Treatment Research Institute
1915, Tri-College University
1916, Tri-County Community College
1917, Tri-County Technical College
1918, Trident Technical College
1919, Trine University
1920, Trinidad State Junior College
1921, Trinity College- Hartford
1922, Trinity University
1923, Trinity Washington University
1924, Troy University
1925, Truckee Meadows Community College
1926, Truman State University
1927, Tufts Medical Center
1928, Tufts University
1929, Tufts University Medford
1930, Tulane University
1931, Tulsa Community College
1932, Turtle Mountain Community College
1933, Tusculum College
1934, Tuskegee University
1935, Tyler Junior College
1936, U.S. Walter Reed Army Inst Of Research
1937, Umpqua Community College
1938, Uniformed Services University Of The Health Sciences
1939, Union College- Lincoln
1940, Union County College
1941, Union Graduate College
1942, Union Institute & University
1943, Union Presbyterian Seminary- Richmond
1944, Union Theological Seminary
1945, Union University- Jackson
1946, United States Air Force Academy
1947, United States Coast Guard Academy
1948, United States Merchant Marine Academy
1949, United States Military Academy
1950, United States Naval Academy
1951, United States University
1952, United Tribes Technical College
1953, Unity College
1954, Universidad Adventista De Las Antillas
1955, Universidad Central Del Caribe
1956, Universidad Del Este
1957, Universidad Del Turabo
1958, Universidad Metropolitana
1959, Universities And State Colleges Of Arizona
1960, University College Of San Juan
1961, University Consortium For Geographic Information Science
1962, University Corporation For Atmospheric Research
1963, University Of Akron
1964, University Of Alabama At Birmingham
1965, University Of Alabama In Huntsville
1966, University Of Alabama - Tuscaloosa
1967, University Of Alabama

1968, University Of Alaska Anchorage
1969, University Of Alaska Fairbanks
1970, University Of Alaska Southeast
1971, University Of Alaska System Of Higher Education
1972, University Of Arizona
1973, University Of Arkansas At Little Rock
1974, University Of Arkansas At Monticello
1975, University Of Arkansas For Medical Sciences
1976, University Of Arkansas Pine Bluff
1977, University Of Arkansas
1978, University Of Arkansas-Fayetteville
1979, University Of Arkansas-Ft. Smith
1980, University Of Baltimore
1981, University Of Bridgeport
1982, University Of California Hastings College Of Law
1983, University Of California-Berkeley
1984, University Of California-Cooperative Extension
1985, University Of California-Davis
1986, University Of California-Irvine
1987, University Of California-Los Angeles
1988, University Of California-Merced
1989, University Of California-Riverside
1990, University Of California-San Diego
1991, University Of California-San Francisco
1992, University Of California-Santa Barbara
1993, University Of California-Santa Cruz
1994, University Of Central Arkansas
1995, University Of Central Florida
1996, University Of Central Missouri
1997, University Of Central Oklahoma
1998, University Of Charleston
1999, University Of Chicago
2000, University Of Cincinnati
2001, University Of Colorado
2002, University Of Colorado Boulder
2003, University Of Colorado Colorado Springs
2004, University Of Colorado Denver And Anschutz Medical Campus
2005, University Of Connecticut
2006, University Of Dallas
2007, University Of Dayton
2008, University Of Delaware
2009, University Of Denver
2010, University Of Detroit Mercy
2011, University Of Dubuque
2012, University Of Evansville
2013, University Of Findlay
2014, University Of Florida
2015, University Of Georgia
2016, University Of Great Falls
2017, University Of Guam
2018, University Of Hartford
2019, University Of Hawaii At Hilo
2020, University Of Hawaii At Manoa
2021, University Of Hawaii At West Oahu
2022, University Of Hawaii
2023, University Of Houston
2024, University Of Houston-Clear Lake
2025, University Of Houston-Downtown
2026, University Of Houston-Victoria
2027, University Of Idaho
2028, University Of Illinois At Chicago
2029, University Of Illinois At Springfield
2030, University Of Illinois At Urbana-Champaign
2031, University Of Illinois
2032, University Of Indianapolis
2033, University Of Iowa
2034, University Of Kansas
2035, University Of Kentucky
2036, University Of La Verne
2037, University Of Louisiana At Lafayette
2038, University Of Louisiana At Monroe
2039, University Of Louisiana System Office
2040, University Of Louisville
2041, University Of Maine
2042, University Of Maine At Augusta
2043, University Of Maine At Machias
2044, University Of Maine At

Presque Isle
2045, University Of Mary
2046, University Of Mary Washington
2047, University Of Maryland Baltimore
2048, University Of Maryland Biotechnology Institute
2049, University Of Maryland Center For Environmental Science
2050, University Of Maryland Eastern Shore
2051, University Of Maryland
2052, University Of Maryland University College
2053, University Of Maryland - Baltimore County
2054, University Of Maryland - College Park
2055, University Of Massachusetts Amherst
2056, University Of Massachusetts Boston
2057, University Of Massachusetts Dartmouth
2058, University Of Massachusetts Lowell
2059, University Of Massachusetts Medical School
2060, University Of Massachusetts
2061, University Of Memphis
2062, University Of Miami
2063, University Of Miami School Of Medicine
2064, University Of Michigan
2065, University Of Michigan-Ann Arbor
2066, University Of Michigan-Dearborn
2067, University Of Michigan-Flint
2068, University Of Minnesota
2069, University Of Minnesota-Crookston
2070, University Of Minnesota-Duluth
2071, University Of Minnesota-Minneapolis
2072, University Of Minnesota-Morris
2073, University Of Minnesota-Rochester
2074, University Of Mississippi Med Ctr
2075, University Of Mississippi
2076, University Of Mississippi - Jackson - Medical Center
2077, University Of Missouri
2078, University Of Missouri-Columbia
2079, University Of Missouri-Kansas City
2080, University Of Missouri-Saint Louis
2081, University Of Montana Western
2082, University Of Montana-Missoula College
2083, University Of Montana
2084, University Of Montevallo
2085, University Of Nebraska At Kearney
2086, University Of Nebraska At Omaha
2087, University Of Nebraska Medical Center
2088, University Of Nebraska
2089, University Of Nebraska-Lincoln
2090, University Of Nevada- Las Vegas
2091, University Of Nevada-Reno
2092, University Of New England
2093, University Of New Hampshire
2094, University Of New Haven
2095, University Of New Mexico Health Scis Ctr
2096, University Of New Mexico
2097, University Of New Orleans
2098, University Of North Alabama
2099, University Of North Carolina At Asheville
2100, University Of North Carolina At Chapel Hill
2101, University Of North Carolina At Charlotte
2102, University Of North Carolina At Greensboro
2103, University Of North Carolina At Pembroke
2104, University Of North Carolina At Wilmington
2105, University Of North Carolina
2106, University Of North Dakota
2107, University Of North Florida
2108, University Of North Texas- Denton
2109, University Of North Texas- Health Science Center
2110, University Of Northern Colorado
2111, University Of Northern Iowa
2112, University Of Notre Dame

2113, University Of Oklahoma Hlth Sciences Ctr
2114, University Of Oklahoma
2115, University Of Oregon
2116, University Of Pennsylvania
2117, University Of Phoenix
2118, University Of Pittsburgh
2119, University Of Pittsburgh-Bradford
2120, University Of Pittsburgh-Pittsburgh
2121, University Of Portland
2122, University Of Puerto Rico
2123, University Of Puerto Rico At Aguadilla
2124, University Of Puerto Rico At Arecibo
2125, University Of Puerto Rico At Bayamon
2126, University Of Puerto Rico At Carolina
2127, University Of Puerto Rico At Cayey
2128, University Of Puerto Rico At Humacao
2129, University Of Puerto Rico At Mayaguez
2130, University Of Puerto Rico At Ponce
2131, University Of Puerto Rico At Rio Piedras
2132, University Of Puerto Rico La Montana Regional Colleges
2133, University Of Puerto Rico Rio Piedras
2134, University Of Puerto Rico-Medical Sciences Campus
2135, University Of Puget Sound
2136, University Of Redlands
2137, University Of Rhode Island
2138, University Of Richmond
2139, University Of Rio Grande
2140, University Of Rochester
2141, University Of Sacred Heart
2142, University Of Saint Francis
2143, University Of Saint Mary-Leavenworth
2144, University Of San Diego
2145, University Of San Francisco
2146, University Of Scranton
2147, University Of Sioux Falls
2148, University Of South Alabama
2149, University Of South Carolina
2150, University Of South Carolina- Aiken
2151, University Of South Carolina- Beaufort
2152, University Of South Carolina- Columbia
2153, University Of South Carolina- Spartanburg
2154, University Of South Dakota
2155, University Of South Florida Polytechnic
2156, University Of South Florida Sarasota-Manatee
2157, University Of South Florida St. Petersburg
2158, University Of South Florida- Tampa
2159, University Of Southern California
2160, University Of Southern Indiana
2161, University Of Southern Maine
2162, University Of Southern Mississippi
2163, University Of St. Francis
2164, University Of St. Thomas (Mn)- Saint Paul
2165, University Of St. Thomas-Houston
2166, University Of Tampa
2167, University Of Tennessee Health Sci Ctr
2168, University Of Tennessee
2169, University Of Tennessee - Agricultural Institute
2170, University Of Tennessee - Chattanooga
2171, University Of Tennessee - Health Science Center
2172, University Of Tennessee - Knoxville
2173, University Of Tennessee - Martin
2174, University Of Tennessee - Tullahoma - Space Institute
2175, University Of Texas At Arlington
2176, University Of Texas At Austin
2177, University Of Texas At Brownsville
2178, University Of Texas At Dallas
2179, University Of Texas At El Paso
2180, University Of Texas At Permian Basin
2181, University Of Texas At San Antonio
2182, University Of Texas At

Tyler
2183, University Of Texas Health Science Center At Houston
2184, University Of Texas Health Science Center At San Antonio
2185, University Of Texas M.D.Anderson Cancer Center
2186, University Of Texas Medical Branch
2187, University Of Texas Southwestern Medical Center
2189, University Of Texas-Pan American
2190, University Of The Cumberlands
2191, University Of The District Of Columbia
2192, University Of The Incarnate Word
2193, University Of The Pacific
2194, University Of The Sciences Philadelphia
2195, University Of The Virgin Islands
2196, University Of The West
2197, University Of Toledo
2198, University Of Toledo - Health Science Campus
2199, University Of Tulsa
2200, University Of Utah
2201, University Of Vermont
2202, University Of Virginia
2203, University Of Virginia College At Wise
2204, University Of Washington-Bothell
2205, University Of Washington-Seattle
2206, University Of Washington-Tacoma
2207, University Of West Alabama
2208, University Of West Florida
2209, University Of West Georgia
2210, University Of Western States
2211, University Of Wisconsin Colleges
2212, University Of Wisconsin
2213, University Of Wisconsin-Eau Claire
2214, University Of Wisconsin-Green Bay
2215, University Of Wisconsin-La Crosse
2216, University Of Wisconsin-Madison
2217, University Of Wisconsin-Milwaukee
2218, University Of Wisconsin-Oshkosh
2219, University Of Wisconsin-Parkside
2220, University Of Wisconsin-Platteville
2221, University Of Wisconsin-River Falls
2222, University Of Wisconsin-Stevens Point
2223, University Of Wisconsin-Stout
2224, University Of Wisconsin-Superior
2225, University Of Wisconsin-Whitewater
2226, University Of Wyoming
2227, University System Of Georgia
2228, Upper Midwest Aerospace Consortium
2229, Urban College Of Boston
2230, Ursinus College
2231, Ursuline College
2232, Utah State University
2233, Utah State University - Price - College Of Eastern Utah
2234, Utah Valley University
2235, Ut-Battelle- Llc-Oak Ridge National Lab
2236, Utica College
2237, Valdosta State University
2238, Valencia Community College
2239, Valley City State University
2240, Valparaiso University
2241, Van Andel Research Institute
2242, Vanderbilt University
2243, Vanguard University
2244, Vassar College
2245, Ventura County Community College District-System Office
2246, Vermilion Community College
2247, Vermont Technical College
2248, Via Christi Regional Medical Center
2249, Villanova University
2250, Vincennes University
2251, Virginia College-Lynchburg
2252, Virginia Commonwealth University
2253, Virginia Community College
2254, Virginia Institute Of Marine Science

				2255, Virginia Military Institute<br>2256, Virginia Polytechnic Institute And State University<br>2257, Virginia State University<br>2258, Virginia Union University<br>2259, Virginia Wesleyan College<br>2260, Viterbo University<br>2261, Voorhees College<br>2262, Wabash College<br>2263, Wadsworth Center<br>2264, Wagner College<br>2265, Wake Forest University<br>2266, Wake Forest University Health Sciences<br>2267, Wake Technical Community College<br>2268, Walden University<br>2269, Waldorf College<br>2270, Walla Walla Community College<br>2271, Walla Walla University<br>2272, Wallace Community College - Dothan<br>2273, Wallace State Community College- Selma<br>2274, Wallace State Community College-Hanceville<br>2275, Walsh College Of Accountancy And Business Administration<br>2276, Walsh University<br>2277, Warren Wilson College<br>2278, Washburn University<br>2279, Washington & Jefferson College<br>2280, Washington And Lee University<br>2281, Washington College<br>2282, Washington State Community College<br>2283, Washington State University<br>2284, Washington University In St. Louis<br>2285, Waubonsee Community College<br>2286, Waukesha County Technical College<br>2287, Wayland Baptist University<br>2288, Wayne Community College<br>2289, Wayne State College<br>2290, Wayne State University<br>2291, Waynesburg University<br>2292, Weatherford College<br>2293, Webb Institute<br>2294, Weber State University<br>2295, Webster University- Saint Louis<br>2296, Weill Medical Coll Of Cornell Univ<br>2297, Wellesley College<br>2298, Wenatchee Valley College<br>2299, Wentworth Institute Of Technology<br>2300, Wesley College<br>2301, Wesleyan College<br>2302, Wesleyan University<br>2303, West Chester University Of Pennsylvania<br>2304, West Georgia Technical College<br>2305, West Hills Community College District<br>2306, West Liberty University<br>2307, West Los Angeles College<br>2308, West Shore Community College<br>2309, West Suburban College Of Nursing<br>2310, West Texas A&M University<br>2311, West Virginia Council For Community And Technical Education<br>2312, West Virginia School Of Osteopathic Medicine<br>2313, West Virginia State University<br>2314, West Virginia University Institute Of Technology<br>2315, West Virginia University<br>2316, West Virginia Wesleyan College<br>2317, Western Carolina University<br>2318, Western Connecticut State University<br>2319, Western Illinois University<br>2320, Western Iowa Tech Community College<br>2321, Western Kentucky University<br>2322, Western Michigan University<br>2323, Western Nebraska Community College<br>2324, Western New England College<br>2325, Western New Mexico University<br>2326, Western Oklahoma State College<br>2327, Western Oregon University<br>2328, Western State College Of Colorado<br>2329, Western Texas College<br>2330, Western University Of Health Sciences<br>2331, Western Washington										

				University<br>2332, Westfield State University<br>2333, Westminster College-Fulton<br>2334, Westminster College- New Wilmington<br>2335, Westminster College- Salt Lake City<br>2336, Westminster Theological Seminary<br>2337, Westmont College<br>2338, Wharton County Junior College<br>2339, Whatcom Community College<br>2340, Wheaton College- Norton<br>2341, Wheaton College-Wheaton<br>2342, Wheeling Jesuit University<br>2343, Wheelock College<br>2344, White Earth Tribal & Community College<br>2345, Whitehead Institute For Biomedical Res<br>2346, Whitman College<br>2347, Whittier College<br>2348, Whitworth University<br>2349, Wichita State University<br>2350, Widener University<br>2351, Wilberforce University<br>2352, Wiley College<br>2353, Wilkes Community College<br>2354, Wilkes University<br>2355, Willamette University<br>2356, William Beaumont Hospital Research Inst<br>2357, William Carey University-Hattiesburg<br>2358, William Jewell College<br>2359, William Mitchell College Of Law<br>2360, William Paterson University<br>2361, Williams College<br>2362, Wilmington College Of Ohio<br>2363, Wilmington University<br>2364, Wilson College<br>2365, Winifred Masterson Burke Med Res Inst<br>2366, Winona State University<br>2367, Winston-Salem State University<br>2368, Winthrop University<br>2369, Wisconsin Lutheran College<br>2370, Wisconsin Technical College<br>2371, Wistar Institute<br>2372, Wittenberg University<br>2373, Wofford College<br>2374, Wolford College<br>2375, Women And Infants Hospital-Rhode Island<br>2376, Woods Hole Oceanographic Institution<br>2377, Worcester Polytechnic Institute<br>2378, Worcester State College<br>2379, Wright Institute<br>2380, Wright State University<br>2381, Wyotech<br>2382, Xavier University<br>2383, Xavier University Of Louisiana<br>2384, Yakima Valley Community College<br>2385, Yale University<br>2386, Yavapai College<br>2387, Yeshiva University<br>2388, York College, Nebraska<br>2389, York College of Pennsylvania<br>2390, York Technical College<br>2391, Yosemite Community College District<br>2392, Youngstown State University<br><br>***Possible label mismatch because of label changes. Check if okay.**<br><br>***Deleted options are okay since those options have no data.**										
author		radio	How many total publications do you have where you are listed as either first author, co-first author, last author or corresponding author (published or in press manuscripts only)?	1, 0<br>2, 1<br>3, 2<br>4, 3<br>5, 4 or more (please specify)										
author_specify		text	Please specify the number of publications.							[author] = '5'				
	Section 4. Finances,													

income	Cost of Living and Family Structure<br>Section 4. Finances, Cost of Living and Family Structure	dropdown	What is your current individual gross (pre tax) income?	1, $39001 - $40000<br>2, $40001 - $42500<br>3, $42501 - $45000<br>4, $45001 - $47500<br>5, $47501 - $50000<br>6, $50001 - $52500<br>7, $52501 - $55000<br>8, Other (please specify)		autocomplete								
plans_change		radio	Have your career plans changed since starting your postdoctoral position?	1, Yes<br>2, No<br>3, Somewhat<br>4, No definite plans<br>1, Yes<br>2, No<br>3, Somewhat<br>4, Not sure<br>***Possible label mismatch because of label changes. Check if okay.**	If yes or somewhat, please specify									
demo		checkbox	Do you identify with one or more of these categories (please select all that apply)?	1, Hispanic / Latino<br>2, White / Caucasian<br>3, Black / African American<br>4, Asian / Asian American<br>5, Native American / Alaska Native<br>6, Pacific Islander / Hawaii Native<br>7, Underrepresented<br>8, Disabled<br>9, LGBTQ<br>10, Veteran / Active Duty Military<br>11, Other (please specify)										
demo_specify		text	Please specify the category(s).							[demo] ='11'				
phd_year		dropdown	In what year did you earn your Ph.D. or other doctoral degree?<br>In what year did you earn your Ph.D. or other doctoral degree?	1, 2016<br>2, 2015<br>3, 2014<br>4, 2013<br>5, 2012<br>6, 2011<br>7, 2010<br>8, 2009 or earlier		autocomplete								
pd_number		radio	How many postdoctoral positions have you held prior to your current position (outside of changes to your postdoctoral status)?<br>Outside of changes to your postdoctoral status, how many different postdoctoral positions have you held?	1, 0<br>2, 1<br>3, 2<br>4, 3 or more please specify										
career_us		radio<br>yesno<br>***Possible data loss if field type changes. Check if okay.**	Do you plan to pursue a career in the U.S., if possible?	1, Yes<br>2, No<br>3, Not sure	If no, please specify country of choice									
				1, AD - Andorra<br>2, AE - United Arab Emirates<br>3, AF - Afghanistan<br>4, AG - Antigua and Barbuda<br>5, AI - Anguilla<br>6, AL - Albania<br>7, AM - Armenia<br>8, AO - Angola<br>9, AQ - Antarctica<br>10, AR - Argentina<br>11, AS - American Samoa<br>12, AT - Austria<br>13, AU - Australia<br>14, AW - Aruba<br>15, AZ - Azerbaijan<br>16, BA - Bosnia and Herzegovina<br>17, BB - Barbados<br>18, BD - Bangladesh<br>19, BE - Belgium<br>20, BF - Burkina Faso<br>21, BG - Bulgaria<br>22, BH - Bahrain<br>23, BI - Burundi<br>24, BJ - Benin<br>25, BL - Saint Barthelemy<br>26, BM - Bermuda<br>27, BN - Brunei<br>28, BO - Bolivia<br>29, BR - Brazil<br>30, BS - Bahamas, The										

career_notus		dropdown	Please specify the country where you plan to pursue your career.	31, BT - Bhutan<br>32, BV - Bouvet Island<br>33, BW - Botswana<br>34, BY - Belarus<br>35, BZ - Belize<br>36, CA - Canada<br>37, CC - Cocos (Keeling) Islands<br>38, CD - Congo, Democratic Republic of the<br>39, CF - Central African Republic<br>40, CG - Congo, Republic of the<br>41, CH - Switzerland<br>42, CI - Cote d'Ivoire<br>43, CK - Cook Islands<br>44, CL - Chile<br>45, CM - Cameroon<br>46, CN - China<br>47, CO - Colombia<br>48, CR - Costa Rica<br>49, CU - Cuba<br>50, CV - Cape Verde<br>51, CW - Curacao<br>52, CX - Christmas Island<br>53, CY - Cyprus<br>54, CZ - Czech Republic<br>55, DE - Germany<br>56, DJ - Djibouti<br>57, DK - Denmark<br>58, DM - Dominica<br>59, DO - Dominican Republic<br>60, DZ - Algeria<br>61, EC - Ecuador<br>62, EE - Estonia<br>63, EG - Egypt<br>64, EH - Western Sahara<br>65, ER - Eritrea<br>66, ES - Spain<br>67, ET - Ethiopia<br>68, FI - Finland<br>69, FJ - Fiji<br>70, FK - Falkland Islands (Islas Malvinas)<br>71, FM - Micronesia, Federated States of<br>72, FO - Faroe Islands<br>73, FR - France<br>74, FX - France, Metropolitan<br>75, GA - Gabon<br>76, GB - United Kingdom<br>77, GD - Grenada<br>78, GE - Georgia<br>79, GF - French Guiana<br>80, GG - Guernsey<br>81, GH - Ghana<br>82, GI - Gibraltar<br>83, GL - Greenland<br>84, GM - Gambia, The<br>85, GN - Guinea<br>86, GP - Guadeloupe<br>87, GQ - Equatorial Guinea<br>88, GR - Greece<br>89, GS - South Georgia and the Islands<br>90, GT - Guatemala<br>91, GU - Guam<br>92, GW - Guinea-Bissau<br>93, GY - Guyana<br>94, HK - Hong Kong<br>95, HM - Heard Island and McDonald Islands<br>96, HN - Honduras<br>97, HR - Croatia<br>98, HT - Haiti<br>99, HU - Hungary<br>100, ID - Indonesia<br>101, IE - Ireland<br>102, IL - Israel<br>103, IM - Isle of Man<br>104, IN - India<br>105, IO - British Indian Ocean Territory<br>106, IQ - Iraq<br>107, IR - Iran<br>108, IS - Iceland<br>109, IT - Italy<br>110, JE - Jersey<br>111, JM - Jamaica<br>112, JO - Jordan<br>113, JP - Japan<br>114, KE - Kenya<br>115, KG - Kyrgyzstan<br>116, KH - Cambodia<br>117, KI - Kiribati<br>118, KM - Comoros<br>119, KN - Saint Kitts and Nevis<br>120, KP - Korea, North<br>121, KR - Korea, South<br>122, KW - Kuwait<br>123, KY - Cayman Islands<br>124, KZ - Kazakhstan<br>125, LA - Laos<br>126, LB - Lebanon<br>127, LC - Saint Lucia<br>128, LI - Liechtenstein<br>129, LK - Sri Lanka<br>130, LR - Liberia<br>131, LS - Lesotho<br>132, LT - Lithuania<br>133, LU - Luxembourg<br>134, LV - Latvia<br>135, LY - Libya<br>136, MA - Morocco<br>137, MC - Monaco<br>138, MD - Moldova<br>139, ME - Montenegro<br>140, MF - Saint Martin<br>141, MG - Madagascar<br>142, MH - Marshall Islands<br>143, MK - Macedonia<br>144, ML - Mali<br>145, MM - Burma		autocomplete				[career_us] = '2'<br>[career_us] = '0'			

				146, MN - Mongolia<br>147, MO - Macau<br>148, MP - Northern Mariana Islands<br>149, MQ - Martinique<br>150, MR - Mauritania<br>151, MS - Montserrat<br>152, MT - Malta<br>153, MU - Mauritius<br>154, MV - Maldives<br>155, MW - Malawi<br>156, MX - Mexico<br>157, MY - Malaysia<br>158, MZ - Mozambique<br>159, NA - Namibia<br>160, NC - New Caledonia<br>161, NE - Niger<br>162, NF - Norfolk Island<br>163, NG - Nigeria<br>164, NI - Nicaragua<br>165, NL - Netherlands<br>166, NO - Norway<br>167, NP - Nepal<br>168, NR - Nauru<br>169, NU - Niue<br>170, NZ - New Zealand<br>171, OM - Oman<br>172, PA - Panama<br>173, PE - Peru<br>174, PF - French Polynesia<br>175, PG - Papua New Guinea<br>176, PH - Philippines<br>177, PK - Pakistan<br>178, PL - Poland<br>179, PM - Saint Pierre and Miquelon<br>180, PN - Pitcairn Islands<br>181, PR - Puerto Rico<br>182, PS - Gaza Strip<br>183, PS - West Bank<br>184, PT - Portugal<br>185, PW - Palau<br>186, PY - Paraguay<br>187, QA - Qatar<br>188, RE - Reunion<br>189, RO - Romania<br>190, RS - Serbia<br>191, RU - Russia<br>192, RW - Rwanda<br>193, SA - Saudi Arabia<br>194, SB - Solomon Islands<br>195, SC - Seychelles<br>196, SD - Sudan<br>197, SE - Sweden<br>198, SG - Singapore<br>199, SH - Saint Helena, Ascension, and Tristan da Cunha<br>200, SI - Slovenia<br>201, SJ - Svalbard<br>202, SK - Slovakia<br>203, SL - Sierra Leone<br>204, SM - San Marino<br>205, SN - Senegal<br>206, SO - Somalia<br>207, SR - Suriname<br>208, SS - South Sudan<br>209, ST - Sao Tome and Principe<br>210, SV - El Salvador<br>211, SX - Sint Maarten<br>212, SY - Syria<br>213, SZ - Swaziland<br>214, TC - Turks and Caicos Islands<br>215, TD - Chad<br>216, TF - French Southern and Antarctic Lands<br>217, TG - Togo<br>218, TH - Thailand<br>219, TJ - Tajikistan<br>220, TK - Tokelau<br>221, TL - Timor-Leste<br>222, TM - Turkmenistan<br>223, TN - Tunisia<br>224, TO - Tonga<br>225, TR - Turkey<br>226, TT - Trinidad and Tobago<br>227, TV - Tuvalu<br>228, TW - Taiwan<br>229, TZ - Tanzania<br>230, UA - Ukraine<br>231, UG - Uganda<br>232, UM - United States Minor Outlying Islands<br>233, UY - Uruguay<br>234, UZ - Uzbekistan<br>235, VA - Holy See (Vatican City)<br>236, VC - Saint Vincent and the Grenadines<br>237, VE - Venezuela<br>238, VG - British Virgin Islands<br>239, VI - Virgin Islands<br>240, VN - Vietnam<br>241, VU - Vanuatu<br>242, WF - Wallis and Futuna<br>243, WS - Samoa<br>244, XK - Kosovo<br>245, YE - Yemen<br>246, YT - Mayotte<br>247, ZA - South Africa<br>248, ZM - Zambia<br>249, ZW - Zimbabwe										
			Has funding outlook changed your willingness to pursue an academic research career in the	1, Yes										

us_funding		radio	U.S.? Has funding outlook changed your willingness to pursue an academic research career in the U.S.?	2, No<br>3, Not sure											

RETURN TO PREVIOUS PAGE
