# Supplementary material for: United States National Postdoc Survey results and the interaction of gender, career choice and mentor impact
Source: eLife. 2018 Dec 18;7:e40189. doi: 10.7554/eLife.40189 (PMC6298783; doi:10.7554/eLife.40189)
Supplement: Supplementary file 1. — Table S1. Population proportion analysis. Number of individual responses required from a total population of 100,000 for 95% and 99% confidence levels, at a 5% margin of error, assuming the true population proportion being measured is between 3% and 50% of the total population. Response values estimated using a population proportion analysis following equations and definitions described in Tintle et al. (Davis, 2009) and at Select Statistical Services Limited (Fleming et al., 2012). Table S2. National data summarized in main text and Figure 1. Table S3. Percentages of postdoc respondents in primary fields per US census region. There was a small, but significant, correlation between region and field (Pearson χ2, n = 7,585, χ2 = 134.145, p < 0.0001). Table S4. Nominal logistic model of gender disparity in pay. Gender remains a significant factor explaining postdoc salary, even when including year of terminal degree, age, partner status, parental status, type of institution, institution control, and satisfaction with mentor. Whole model n = 7,280, χ2 = 2589.077, p < 0.0001, AICc = 28458.9, BIC = 30056.2. Table S5. Gender salary disparity by field. Table S6. Respondent reported salaries adjusted to cost of living. Table S7. Factors included in nominal logistic regression models of satisfaction with mentor and primary career plan. [file elife-40189-supp1.docx]

Supplementary Materials for

**United States National Postdoc Survey Results and the Interaction of Gender, Career Choice, and Mentor Impact**

**Authors**

Sean C. McConnell^1^*, Erica L. Westerman^2^*+, Joseph F. Pierre^3^, Erin J. Heckler^4^, Nancy B. Schwartz^1^

**Affiliations**

University of Chicago^1^, University of Arkansas^2^, University of Tennessee Health Sciences Center,^3^ Washington University in St. Louis^4^

*Contributed equally

+Corresponding author: Erica L. Westerman, ewesterm@uark.edu

**This PDF file includes:**

Tables S1 to S7

Table S1

| Sample Proportion | 95% Confidence Level | 99% Confidence Level |
| --- | --- | --- |
| 50% | 383 | 660 |
| 40% | 368 | 633 |
| 30% | 322 | 555 |
| 20% | 246 | 423 |
| 10% | 139 | 239 |
| 5% | 73 | 126 |
| 3% | 45 | 78 |

**Table S1**: **Population proportion analysis**.

Number of individual responses required from a total population of 100,000 for 95% and 99% confidence levels, at a 5% margin of error, assuming the true population proportion being measured is between 3 and 50% of the total population*.* Response values estimated using a population proportion analysis following equations and definitions described in Tintle et al ^31^ and at Select Statistical Services Limited^32^.

Table S2

|  | |  |  |
| --- | --- | --- | --- |
| How satisfied are you with the mentoring that you receive in your laboratory? | |  |  |
| Very satisfied | 1888 | 25.0% |  |
| Satisfied | 2679 | 35.4% |  |
| Somewhat satisfied | 1555 | 20.5% |  |
| Not very satisfied | 842 | 11.1% |  |
| Not at all satisfied | 536 | 7.1% |  |
| No comment | 66 | 0.9% |  |
| Total answered | 7566 | 100% |  |
|  |  |  |  |
| Have you ever received a performance evaluation? | |  |  |
| Yes | 3137 | 41.4% |  |
| No | 4435 | 58.6% |  |
| Total answered | 7572 | 100% |  |
|  |  |  |  |
| What are your primary long term career plans? | |  |  |
| Academic (primarily research-based) | 4378 | 57.7% |  |
| Industrial Research | 1349 | 17.8% |  |
| Academic (primarily teaching-based) | 786 | 10.4% |  |
| Other | 396 | 5.2% |  |
| Government or Non-profit | 341 | 4.5% |  |
| Research Administration | 90 | 1.2% |  |
| Consulting | 87 | 1.1% |  |
| Science writing or publishing | 74 | 1.0% |  |
| Science policy | 47 | 0.6% |  |
| Patent law or tech transfer | 33 | 0.4% |  |
| Total answered | 7581 | 100% |  |
|  |  |  |  |
| How confident are you that you will attain your specified career plan? | |  |  |
| Very confident | 908 | 12.0% |  |
| Confident | 1982 | 26.2% |  |
| Somewhat confident | 2743 | 36.3% |  |
| Not very confident | 1361 | 18.0% |  |
| Not at all confident | 458 | 6.1% |  |
| Not sure | 108 | 1.4% |  |
| Total answered | 7560 | 100% |  |
|  |  |  |  |

| Have your career plans changed since starting your postdoctoral position? | |  |
| --- | --- | --- |
| Yes | 1565 | 20.6% |
| Somewhat | 1892 | 25.0% |
| No | 3777 | 49.8% |
| No definite plans | 350 | 4.6% |
| Total answered | 7584 | 100% |
|  |  |  |
| How supportive of your career plan is your advisor? | |  |
| Very supportive | 2830 | 37.4% |
| Supportive | 2039 | 27.0% |
| Somewhat supportive | 1273 | 16.8% |
| Not supportive | 421 | 5.6% |
| My advisor is not aware of my career goals | 994 | 13.1% |
| Total answered | 7557 | 100% |
|  |  |  |
| Are you satisfied with the professional development offerings from your institution? | |  |
| Yes | 6011 | 80.3% |
| No | 1471 | 19.7% |
| Total answered | 7482 | 100% |
|  |  |  |
| What is your current individual gross (pre-tax) income? | |  |
| $39001-40000 | 492 | 6.5% |
| $40001-42500 | 1126 | 14.9% |
| $42501-45000 | 1827 | 24.2% |
| $45001-47500 | 865 | 11.5% |
| $47501-50000 | 977 | 13.0% |
| $50001-52500 | 599 | 7.9% |
| $52501-55000 | 527 | 7.0% |
| Other (less) | 353 | 4.7% |
| Other (more) | 750 | 9.9% |
| Other (please specify) | 33 | 0.4% |
| Total answered | 7549 | 100% |
|  |  |  |
| What is your gender? | |  |
| Female | 4014 | 53.1% |
| Male | 3546 | 46.9% |
| Total answered | 7560 | 100% |
|  |  |  |

| What is the gender of your mentor? | |  |
| --- | --- | --- |
| Female | 2208 | 29.4% |
| Male | 5288 | 70.6% |
| Total answered | 7496 | 100% |
|  |  |  |
| What is your residency status in the U.S.? | |  |
| U.S. Citizen | 3718 | 49.1% |
| Permanent Resident | 453 | 6.0% |
| J1 | 2207 | 29.2% |
| H1b | 662 | 8.7% |
| F1 | 395 | 5.2% |
| Other | 134 | 1.8% |
| Total answered | 7569 | 100% |
|  |  |  |
| In what year did you earn your Ph.D. or other doctoral degree? | |  |
| 2016 | 481 | 6.5% |
| 2015 | 1855 | 24.9% |
| 2014 | 1637 | 22.0% |
| 2013 | 1209 | 16.2% |
| 2012 | 881 | 11.8% |
| 2011 | 524 | 7.0% |
| 2010 | 339 | 4.6% |
| 2009 or earlier | 519 | 7.0% |
| Total answered | 7445 | 100% |

Do you identify with one or more of these categories (please select all that apply)?*

| Hispanic / Latino | 513 | 6.6% |
| --- | --- | --- |
| White / Caucasian | 4674 | 60.3 % |
| Black / African American | 202 | 2.6% |
| Asian / Asian American | 1924 | 24.8% |
| Alaska Native / Hawaii Native /  Native American / Pacific Islander | 53 | 0.7% |
| Underrepresented | 218 | 2.8% |
| Other (underrepresented-may refer to factors other than race or ethnicity) | 168 | 2.2% |
| Total* | 7752 | 100% |
| LGBTQ | 240 | 3.2% |
| Veteran/ Active Duty Military | 10 | 0.1% |
| Disabled | 42 | 0.6% |
| Total Number of Respondents | 7603 |  |

*Due to multiple selections, the total may add up to more than 7,603.

**Table S2:** **National data summarized in main text and Figure 1**.

**Table S3**

| **Primary Field** | **Midwest** | **Northeast** | **South** | **West** |
| --- | --- | --- | --- | --- |
| Engineering | 145 (7.85%) | 201 (8.08%) | 143 (8.21%) | 134 (8.88%) |
| Environmental Sciences | 76 (4.12%) | 56 (2.25%) | 72 (4.14%) | 85 (5.63%) |
| Humanities | 14 (0.76%) | 19 (0.76%) | 18 (1.03%) | 14 (0.93%) |
| Life Sciences | 966 (52.33%) | 1464 (58.82%) | 967 (55.54%) | 793 (52.55%) |
| Medicine | 248 (13.43%) | 353 (14.18%) | 225 (12.92%) | 134 (8.88%) |
| Other Field | 9 (0.49%) | 9 (0.36%) | 13 (0.75%) | 5 (0.33%) |
| Physical Sciences | 238 (12.89%) | 195 (7.83%) | 186 (10.68%) | 233 (15.44%) |
| Psychology | 88 (4.77%) | 118 (4.74%) | 67 (3.85%) | 56 (3.71%) |
| Social Sciences | 62 (3.36%) | 74 (2.97%) | 50 (2.87%) | 55 (3.64%) |
| **Total** | **1846 (100%)** | **2489 (100%)** | **1741 (100%)** | **1509 (100%)** |

**Table S3**: **Percentages of postdoc respondents in primary fields per U.S. census region**.

There was a small, but significant, correlation between region and field (Pearson χ^2^, n=7,585, χ^2^=134.145, p<0.0001).

**Table S4**

| **Factor** | **χ^2^** | **p-value** |
| --- | --- | --- |
| Gender | 51.330 | <0.0001 |
| Year of PhD | 724.886 | <0.0001 |
| Carnegie Classification | 776.166 | <0.0001 |
| Control (public/private) | 415.953 | <0.0001 |
| Self-identify as white/Caucasian | 271.243 | <0.0001 |
| Age | 40.781 | 0.268 |
| Married/Partnered | 10.044 | 0.347 |
| Children | 8.865 | 0.268 |
| Mentor satisfaction | 61.857 | 0.048 |

**Table S4**: **Nominal logistic model of gender disparity in pay**.

Gender remains a significant factor explaining postdoc salary, even when including year of terminal degree, age, partner status, parental status, type of institution, institution control, and mentor satisfaction. Whole model n=7,280, χ^2^=2589.077, p<0.0001, AICc=28458.9, BIC=30056.2.

**Table S5**

| **Field** | **Female Average** | **Male Average** | **N** | **χ^2^** | **p-value** |
| --- | --- | --- | --- | --- | --- |
| Engineering | 48,320.60 | 49,611.89 | 616 | 12.377 | 0.1929 |
| Environmental Sciences | 47,650.37 | 49,111.34 | 285 | 8.952 | 0.4417 |
| Humanities | 46,290.79 | 47,000.00 | 66 | 4.119 | 0.846 |
| Life Sciences | 45,510.71 | 46,197.05 | 4158 | 33.775 | <0.0001 |
| Medicine | 46,766.47 | 47,060.58 | 943 | 16.153 | 0.0638 |
| Physical Sciences | 51,664.11 | 51,443.88 | 843 | 14.343 | 0.1106 |
| Psychology | 44,829.11 | 45,494.19 | 324 | 4.305 | 0.8902 |
| Social Sciences | 48,967.03 | 49,654.11 | 239 | 5.788 | 0.7609 |

**Table S5**: **Gender salary disparity by field.**

**Table S6**

| US State / County | Average Postdoc Income (2016 NPS) | Adjusted Postdoc Income (2016 COLI data from C2ER) | City with 50 or More Respondents |
| --- | --- | --- | --- |
| Alabama | $42,960 | $48,394 |  |
| Alaska | $48,750 | $37,305 |  |
| Arkansas | $42,030 | $45,127 |  |
| Arizona | $45,748 | $48,305 |  |
| Maricopa, AZ | $46,230 | $47,709 | Tempe |
| Pima, AZ | $45,237 | $48,642 | Tucson |
| California | $48,576 | $32,036 |  |
| Alameda, CA | $48,646 | $32,780 | Berkeley |
| Los Angeles, CA | $52,427 | $38,302 | Pasadena |
| Yolo, CA | $46,183 | $39,711 | Davis |
| San Diego, CA | $45,368 | $31,462 | La Jolla |
| San Francisco, CA | $47,490 | $26,800 | San Francisco |
| Santa Clara, CA | $52,723 | $23,653 | Palo Alto |
| Colorado | $47,184 | $42,778 |  |
| Denver, CO | $44,993 | $40,792 | Denver |
| Connecticut | $45,776 | $36,975 |  |
| New Haven, CT | $45,758 | $36,932 | New Haven |
| Delaware | $45,179 | $41,988 |  |
| District of Columbia | $49,947 | $33,521 | Washington |
| Florida | $44,394 | $45,283 |  |
| Alachua, FL | $43,460 | $44,392 | Gainesville |
| Georgia | $43,362 | $43,933 |  |
| Clarke, GA | $41,619 | $42,168 | Athens |
| DeKalb, GA | $45,104 | $45,699 | Atlanta |
| Hawaii | $54,375 | $28,603 |  |
| Idaho | $45,313 | $51,668 |  |
| Illinois | $45,889 | $39,032 |  |
| Cook, IL | $45,611 | $38,840 | Chicago |
| Indiana | $45,655 | $49,669 |  |
| Tippecanoe, IN | $42,806 | $49,487 | West Lafayette |
| Iowa | $44,938 | $46,935 |  |
| Johnson, IA | $45,083 | $46,718 | Iowa City |
| Kansas | $43,422 | $47,075 |  |
| Douglas, KS | $42,815 | $46,588 | Lawrence |
| Kentucky | $39,125 | $43,049 |  |
| Louisiana | $45,012 | $46,491 |  |
| Maine | $45,500 | $40,661 |  |
| Maryland | $49,901 | $39,862 |  |
| Baltimore City, MD | $46,718 | $40,448 | Baltimore |
| Montgomery, MD | $53,631 | $39,993 | Bethesda |
| Prince George’s, MD | $50,331 | $37,532 | College Park |
| Massachusetts | $47,908 | $33,058 |  |
| Middlesex, MA | $48,433 | $34,325 | Cambridge |
| Suffolk, MA | $47,454 | $32,085 | Boston |
| Michigan | $45,321 | $45,011 |  |
| Ingham, MI | $44,165 | $47,901 | East Lansing |
| Washtenaw, MI | $45,757 | $43,745 | Ann Arbor |
| Minnesota | $43,996 | $42,254 |  |
| Hennepin, MN | $43,955 | $41,663 | Minneapolis |
| Mississippi | $41,650 | $48,922 |  |
| Missouri | $44,388 | $48,509 |  |
| Boone, MO | $43,030 | $45,343 | Columbia |
| St. Louis, MO | $44,916 | $49,741 | St. Louis |
| Montana | $47,850 | $47,517 |  |
| Nebraska | $42,132 | $46,043 |  |
| Nevada | $42,313 | $40,520 |  |
| New Hampshire | $43,975 | $41,369 |  |
| New Jersey | $48,026 | $39,398 | Princeton |
| New Mexico | $56,736 | $59,848 |  |
| Los Alamos, NM | $60,000 | $63,291 | Los Alamos |
| New York | $48,356 | $26,027 |  |
| Monroe, NY | $47,311 | $46,797 | Rochester |
| New York, NY | $50,811 | $22,304 | New York |
| North Carolina | $44,986 | $47,325 |  |
| Orange, NC | $45,461 | $47,404 | Chapel Hill |
| North Dakota | $43,313 | $45,005 |  |
| Ohio | $44,869 | $49,493 |  |
| Franklin, OH | $43,978 | $48,919 | Columbus |
| Hamilton, OH | $46,443 | $50,981 | Cincinnati |
| Oklahoma | $44,333 | $52,660 |  |
| Oregon | $45,900 | $42,015 |  |
| Benton, OR | $46,996 | $46,030 | Corvallis |
| Jackson, OR | $45,777 | $44,836 | Medford |
| Multnomah, OR | $45,426 | $35,378 | Portland |
| Pennsylvania | $45,361 | $43,685 |  |
| Allegheny, PA | $44,561 | $47,480 | Pittsburgh |
| Philadelphia, PA | $46,293 | $39,066 | Philadelphia |
| Rhode Island | $47,266 | $38,743 | Providence |
| South Carolina | $44,494 | $44,244 |  |
| Charleston, SC | $45,634 | $43,921 | Charleston |
| South Dakota | $41,250 | $43,977 |  |
| Tennessee | $47,787 | $54,644 |  |
| Texas | $44,766 | $47,202 |  |
| Dallas, TX | $44,910 | $44,776 | Dallas |
| Harris, TX | $44,896 | $45,441 | Houston |
| McLennan, TX | $45,446 | $51,527 | Waco |
| Utah | $44,959 | $48,033 |  |
| Salt Lake, UT | $44,873 | $47,941 | Salt Lake City |
| Vermont | $40,667 | $48,183 |  |
| Virginia | $44,880 | $46,967 |  |
| Washington | $47,508 | $33,181 |  |
| King, WA | $47,374 | $32,694 | Seattle |
| West Virginia | $39,875 | $41,710 |  |
| Wisconsin | $44,424 | $41,791 | Madison |
| Wyoming | $53,750 | $58,679 |  |

**Table S6**: **Respondent reported salaries adjusted to cost of living.**

**Table S7**

| **Factor** | **Included in Mentor Satisfaction model** | **Significant factors in model of best fit** | **Included in Primary Career Plan model** | **Significant factors in model of best fit** |
| --- | --- | --- | --- | --- |
| **Institution Based** |  |  |  |  |
| Carnegie classification | X |  | X |  |
| Control (Private versus Public) | X |  | X |  |
| **Mentor Based** |  |  |  |  |
| Frequency of mentor meetings | X | **X** | X |  |
| Received training in grant writing | X |  | X |  |
| Received training in mentoring | X | **X** | X |  |
| Received training in pedagogy | X |  | X | **X** |
| Academic rank of mentor | X | **X** | X | **X** |
| Mentor gender | X |  | X |  |
| Postdoc satisfaction with mentor |  |  | X |  |
| Perceived mentor support of career plan | X | **X** | X | **X** |
| **Postdoc Productivity** |  |  |  |  |
| Hours worked / week | X |  | X | **X** |
| Total number of publications while a postdoc | X |  | X | **X** |
| Number of first, last, or corresponding author publications | X |  | X | **X** |
| Conferences attended | X |  | X | **X** |
| **Career Plans** |  |  |  |  |
| Primary career plan | X |  |  |  |
| Whether long term career plans have changed | X |  | X | **X** |
| Feeling of career preparedness | X | **X** | X | **X** |
| Perception of academic job market | X | **X** | X |  |
| Job search intensity | X | **X** | X | **X** |
| Plan to pursue career in U.S. | X |  | X | **X** |
| **Postdoc Demographics** |  |  |  |  |
| Primary field of study | X | **X** | X | **X** |
| Postdoc gender | X |  | X | **X** |
| Postdoc age | X |  | X |  |
| Marital/Partnered | X |  | X |  |
| Has children | X |  | X |  |
| Residency status in U.S. | X |  | X | **X** |
| PhD in U.S. | X |  | X |  |

**Table S7**: **Factors included in nominal logistic regression models of mentor satisfaction and primary career plan.**
